# Bioengineering a bacterial pathogen to assemble its own particulate vaccine capable of inducing cellular immunity

**DOI:** 10.1038/srep41607

**Published:** 2017-02-02

**Authors:** Jason W. Lee, Natalie A. Parlane, D. Neil Wedlock, Bernd H. A. Rehm

**Affiliations:** 1 Institute of Fundamental Sciences, Massey University, Palmerston North, New Zealand; 2 AgResearch, Hopkirk Research Institute, Palmerston North, New Zealand; 3 The MacDiarmid Institute for Advanced Materials and Nanotechnology, Wellington, New Zealand; 4 PolyBatics, Palmerston North, New Zealand

## Abstract

Many bacterial pathogens naturally form cellular inclusions. Here the immunogenicity of polyhydroxyalkanoate (PHA) inclusions and their use as particulate vaccines delivering a range of host derived antigens was assessed. Our study showed that PHA inclusions of pathogenic *Pseudomonas aeruginosa* are immunogenic mediating a specific cell-mediated immune response. Protein engineering of the PHA inclusion forming enzyme by translational fusion of epitopes from vaccine candidates outer membrane proteins OprI, OprF, and AlgE mediated self-assembly of PHA inclusions coated by these selected antigens. Mice vaccinated with isolated PHA inclusions produced a Th1 type immune response characterized by antigen-specific production of IFN-γ and IgG2c isotype antibodies. This cell-mediated immune response was found to be associated with the production of functional antibodies reacting with cells of various *P. aeruginosa* strains as well as facilitating opsonophagocytic killing. This study showed that cellular inclusions of pathogenic bacteria are immunogenic and can be engineered to display selected antigens suitable to serve as particulate subunit vaccines against infectious diseases.

Many bacteria including various human pathogens form polymeric intracellular inclusions such as e.g. polyhydroxyalkanoate (PHA) inclusions which serve as energy and carbon storage material^1^,^2^. While cell surface structures of pathogens had been the focus of studies towards identifying vaccine candidate antigens, the immunogenicity of intracellular structures had not been studied. However nano-/microsized intracellular structures such as polymer inclusions might serve as particulate vaccines suitable for efficient antigen delivery. Particulate antigen delivery systems are being increasingly considered for vaccine formulations evidenced by recent successful application and commercialization of particle-based vaccines^3^,^4^. PHA beads had been previously shown to enable delivery of antigens inducing protective immunity in animal models against tuberculosis^5^,^6^ and hepatitis C^7^,^8^. PHAs are deposited as spherical cytoplasmic inclusions surrounded by proteins^1^,^9^. Protein engineering of one of these coating proteins, the PHA synthase (PhaC_Re_), which catalyzes polyhydroxybutyrate (PHB) formation^10^,^11^,^12^,^13^ enabled antigen display on PHB beads inducing a specific and protective immune response^5^,^8^,^14^,^15^. Vaccine candidate antigens formulated as particles (<1 μm) showed enhanced immunogenicity due to an efficient cellular uptake by professional antigen presenting cells^16^.

Here we selected *Pseudomonas aeruginosa* as a model human pathogen because it naturally forms PHA inclusions and traditional vaccine development approaches were unsuccessful^17^. Its PHA is composed of medium chain length 3-hydroxy fatty acids (MCL) which polymerization is catalyzed by the MCL-PHA synthase (e.g. PhaC1_Pa_)^1^,^2^.

*P. aeruginosa* is one of the leading causes of nosocomial infections and causes serious life-threatening infections due to intrinsic and acquired antibiotic resistances^17^. Immuno-compromised individuals are most at risk, such as those with severe burns and wounds, infected by human immunodeficiency virus (HIV) as well as cystic fibrosis (CF) patients^18^. Vaccines provide a strategy for prevention of the disease caused by *P. aeruginosa*^19^.

Vaccine candidates include outer membrane proteins (OMPs), flagellin and pilin, toxins as well as killed or live attenuated whole cells^17^,^20^. The most promising immunogens are the major OMP F (OprF) and outer membrane lipoprotein I (OprI) which are highly conserved, serotype independent and well tolerated^21^,^22^. Vaccination studies in animals have shown long-lived antibody titers and broad protection against all *P. aeruginosa* serotypes^23^. However high levels of antibodies were associated with more severe lung disease^24^. It has been suggested that a CD4^+^ Th1 type cell mediated response maybe more protective^24^,^25^,^26^, and that OprI vaccination can modulate the immune response from a CD4^+^ Th2 towards a CD4^+^ Th1 cell mediated response^27^. OprI vaccination induced protection in mice^28^. OMP AlgE, the alginate pore, may provide an alternative target for vaccine development. AlgE is overproduced in the mucoid alginate overproducing variant found in the lung of CF patients and has been suggested to be immunogenic^29^,^30^. The crystal structure of AlgE revealed a 18-stranded β-barrel with extended extracellular loops representing possible cell surface exposed antigenic epitopes^31^,^32^. The use of immunogenic epitopes of OprF fused with OprI have been the main candidates for use in *P. aeruginosa* vaccine studies^21^,^22^,^33^, and have shown synergistic effects^34^.

In this study we investigated the immunogenicity of cellular inclusions formed by the human pathogen, *P. aeruginosa.* Immunological properties of PHA inclusions encouraged to engineer *P. aeruginosa* for the production of antigen-displaying PHA inclusions by harnessing its inherent PHA production system. These PHA inclusions were engineered to display selected vaccine antigens of the same host at high density while associated host cell components might serve as additional antigens enhancing the induction of broadly protective immunity and/or having adjuvant properties. This is the first study investigating the immunological properties of cellular polymer inclusions of pathogenic bacteria and to utilize the pathogens own inclusions as carrier of its own antigens to be used as a particulate vaccine.

## Results

### Bioengineering of *P. aeruginosa* for self-assembly of antigen-displaying PHA inclusions

**Figure 1:**
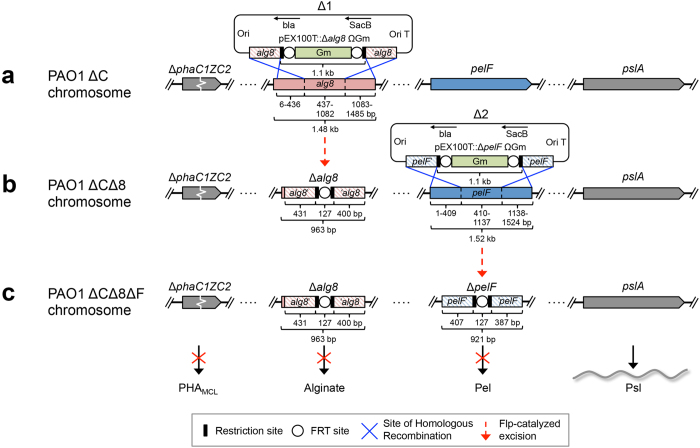
A schematic of the generation of *P. aeruginosa* knockout mutant PAO1 ΔCΔ8ΔF. In order to promote the production of PHA_MCL_ inclusions and vaccine candidate EPS Psl, site-directed homologous recombination was used to delete major parts of (**a**) *alg8* and (**b**) *pelF* genes encoding a glycosyltransferase in the PHA negative mutant PAO1 Δ*phaC1ZC2.* (**c**) Resultant triple mutant strain is defective in PHA/alginate/pel polysaccharide was verified by DNA sequencing (see Supplementary Fig. 1).

To enable the production of antigen-associated PHA_MCL_ inclusions mediated solely by the introduced PHA synthase (PhaC1_Pa_ = non-engineered wildtype) and its fusion protein derivatives (engineered to incoprarate vaccine candidate antigens), an isogenic PHA_MCL_ deficient strain PAO1 Δ*phaC1ZC2* was employed. To promote production of PHA_MCL_ and the vaccine candidate exopolysaccharide (EPS) Psl, essential genes for competing biosynthesis pathways towards the production of alginate and the glucose-rich Pel polysaccharide, respectively, were deleted (Fig. 1a–c and Supplementary Fig. 1)^35^.

**Figure 2:**
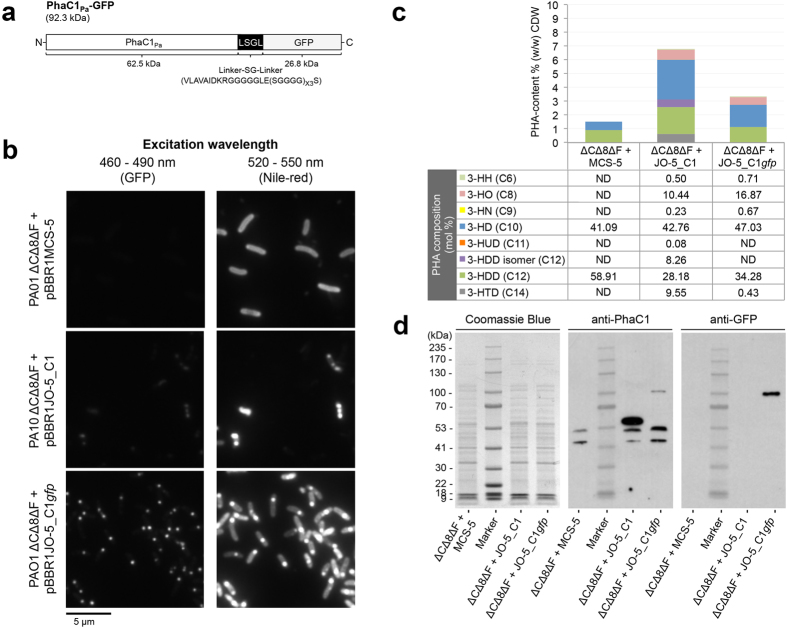
Assessment of the tolerance of the class II PHA synthase (PhaC1_Pa_) to C terminal fusion. (**a**) Schematic representation of fusion protein PhaC1_Pa_-GFP for assessment of class II PHA synthase tolerance to C terminal fusion. (**b**) Fluorescence microscopy analysis of Nile red stained *P. aeruginosa* PAO1 ΔCΔ8ΔF cultures harboring various plasmids grown under PHA_MCL_ accumulating conditions for 24 h and visualized for GFP and *in vivo* PHA_MCL_ inclusions (white arrow). (**c**) Quantification and compositional analysis of PHA_MCL_ in whole-cell by Gas chromatography-mass spectrometry (GC/MS). (**d**) SDS-PAGE and immunoblot analysis of cell lysates to confirm the production of fusion protein. ΔCΔ8ΔF + MCS-5, PAO1 ΔCΔ8ΔF + pBBR1MCS-5; ΔCΔ8ΔF + JO-5_C1, PAO1 ΔCΔ8ΔF + pBBR1JO-5_C1; ΔCΔ8ΔF + JO-5_C1*gfp*, PAO1 ΔCΔ8ΔF + pBBR1JO-5_C1*gfp*; ND, not detected; 3-HH (C6), methyl 3-hydroxyhexanoate; 3-HO (C8), methyl 3-hydroxyoctanoate; 3-HN (C9), methyl 3-hydroxynonanoate; 3-HD (C10), methyl 3-hydroxydecanoate; 3-HUD (C11), methyl 3-hydroxyundecanoate; 3-HDD isomer (C12), methyl 3-hydroxydodecanoate isomer; 3-HDD (C12), methyl 3-hydroxydodecanoate; 3-HTD (C14), methyl 3-hydroxytetradecanoate.

Formation of PHA_MCL_ inclusions mediated by recombinant PhaC1_Pa_ (natural wildtype inclusions) or PhaC1_Pa_-GFP was assessed by fluorescence microscopy, GC/MS, and immunoblot analysis (Fig. 2a–d). In order to assess whether PhaC1_Pa_ tolerates C terminal translational fusions, GFP was fused to its C terminus. A designed linker^10^ was inserted in order to retain functionality of PhaC1_Pa_ and to display the fusion partner GFP on the surface of PHA_MCL_ beads (Fig. 2a). Colocalization of fluorescent foci for GFP and PHA_MCL_ visualized within PAO1 ΔCΔ8ΔF cells producing PhaC1_Pa_-GFP indicated that the GFP fusion to C terminus of PhaC1_Pa_ did not abolish PhaC1_Pa_ activity and implies that GFP fused to the C terminus of this class II PHA synthase was functionally displayed on the surface of the PHA_MCL_ inclusion *in vivo* (Fig. 2b).

Display of GFP on PHA inclusions anchored via fusion to the C terminus of PhaC1_Pa_ was observed by fluorescence microscopy expanding the scope of PhaC1_Pa_ engineering to C terminally fusible antigens. Selected epitopes of the OMPs OprF, OprI (lipoprotein), and AlgE (alginate secretion porin) from *P. aeruginosa* were used as vaccinate candidates to be immobilized to the surface of PHA inclusions. Selection of antigenic epitopes of OprF and OprI was based on previous studies that demonstrated protective immunity in animal models (Supplementary Fig. 2a). Antigenic epitopes of AlgE were selected based on its structure using B-cell antigenic epitope prediction method EPCES (Supplementary Fig. 2b).

**Figure 3:**
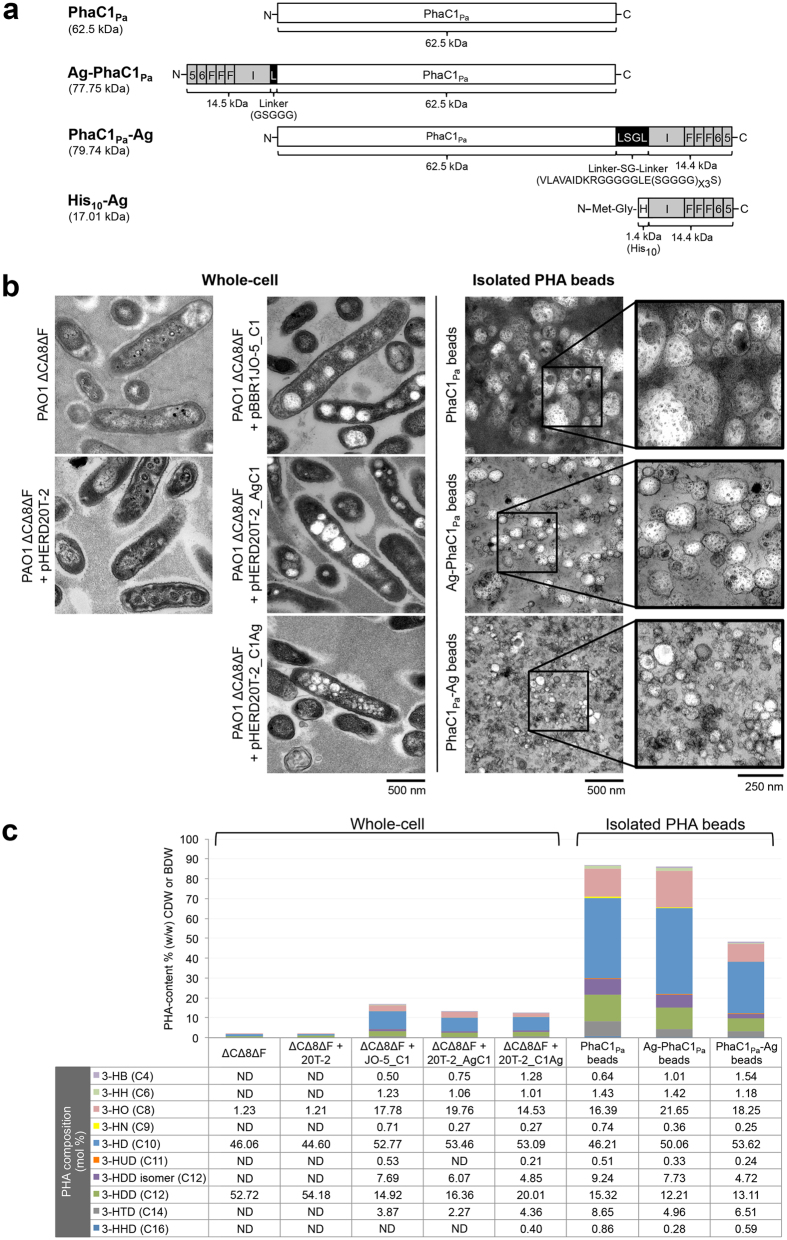
Bioengineering and production of vaccine PHA_MCL_ inclusions *in vivo*. PAO1 ΔCΔ8ΔF cells harboring various plasmids cultivated under PHA_MCL_ accumulating condition for 48 h and their respective isolated PHA_MCL_ beads. (**a**) A schematic representation of various fusion proteins which mediate PHA_MCL_ bead production in strain PAO1 ΔCΔ8ΔF or recombinant protein production in *E. coli* (see Supplementary Fig. 3a,b). (**b**) Accumulation and size of PHA_MCL_ inclusions were analysised by Transmission Electron Microscopy (TEM) in whole-cells and of the isolated PHA_MCL_ bead material. (**c**) Quantification and compositional analysis of PHA_MCL_ using GC/MS. BDW, percentage of the bead dry weight; ΔCΔ8ΔF + MCS-5, PAO1 ΔCΔ8ΔF + pBBR1MCS-5; ΔCΔ8ΔF + JO-5_C1, PAO1 ΔCΔ8ΔF + pBBR1JO-5_C1; ΔCΔ8ΔF + JO-5_C1*gfp*, PAO1 ΔCΔ8ΔF + pBBR1JO-5_C1*gfp*; ND, not detected; 3-HH (C4), methyl 3-hydroxybutanoate; 3-HH (C6), methyl 3-hydroxyhexanoate; 3-HO (C8), methyl 3-hydroxyoctanoate; 3-HN (C9), methyl 3-hydroxynonanoate; 3-HD (C10), methyl 3-hydroxydecanoate; 3-HUD (C11), methyl 3-hydroxyundecanoate; 3-HDD isomer (C12), methyl 3-hydroxydodecanoate isomer; 3-HDD (C12), methyl 3-hydroxydodecanoate; 3-HTD (C14), methyl 3-hydroxytetradecanoate; 3-HHD (C16), methyl 3-hydroxyhexadecanote.

Antigenic epitopes of AlgE, OprF, and OprI were combined as a single fusion antigen (OprI/F-AlgE) and translationally fused to either the N or C terminus of PhaC1_Pa_ and the impact on the production of PHA_MCL_ inclusions as well as the functionality of the fusion partners was analyzed (Fig. 3a–c).

Recombinant *P. aeruginosa* expressing the various genes, PhaC1_Pa_ (wildtype control) or Ag-PhaC1_Pa_ (antigens fused to N terminus) or PhaC1_Pa_-Ag (antigens fused to C terminus) accumulated PHA_MCL_ inclusions, enabling subsequent isolation as PHA_MCL_ bead material as observed by TEM (Fig. 3b). Interestingly, PhaC1_Pa_-Ag mediated production of significantly smaller inclusion (15 to 186 nm, average 48 nm ± 1.16 s.e.m) compared to Ag-PhaC1_Pa_ fusion protein (46 to 316 nm, average 130 nm ± 1.98 s.e.m) and PhaC1_Pa_ protein (45 to 377 nm, average 172 nm ± 2.61 s.e.m).

Quantification and composition of intracellular PHA_MCL_ and purity of the isolated PHA_MCL_ bead material was assessed by GC/MS analysis (Fig. 3c). Low levels of 3-hydroxyalkanoic acids mainly composed of 3-hydroxydecanoate (C10) and 3-hydroxydodecanoate (C12) likely derived from rhamolipid synthesis^36^ contributing to about 1.8% (w/w) of CDW were detected in PHA_MCL_ negative control i.e. means in cells haboring only vector pHERD20T. PAO1 ΔCΔ8ΔF cells harboring the plasmid encoding PhaC1_Pa_ accumulated PHA_MCL_ contributing to about 17% (w/w) of cellular dry weight (CDW), while strain PAO1 ΔCΔ8ΔF harboring the plasmid encoding Ag-PhaC1_Pa_ or PhaC1_Pa_-Ag accumulated PHA_MCL_ contributing to about 13% and 12.5% (w/w) of CDW, respectively. The composition of the PHA_MCL_, i.e. the molar fractions of comonomers, between the different PHA_MCL_ beads showed only slight variation. The beads were composed mainly of 3-hydroxyoctanoate (C8), 3-hydroxydecanoate (C10) and 3-hydroxydodecanoate (C12) and reflected the composition of PHA_MCL_ in whole cells. PHA_MCL_ purity of the isolated bead material is displayed as percentage of the bead dry weight (BDW). Both PhaC1_Pa_ and Ag-PhaC1_Pa_ beads were purified to approximately 87% of PHA_MCL_ content, while PhaC1_Pa_-Ag beads contained only 48.5% of PHA_MCL_.

**Figure 4:**
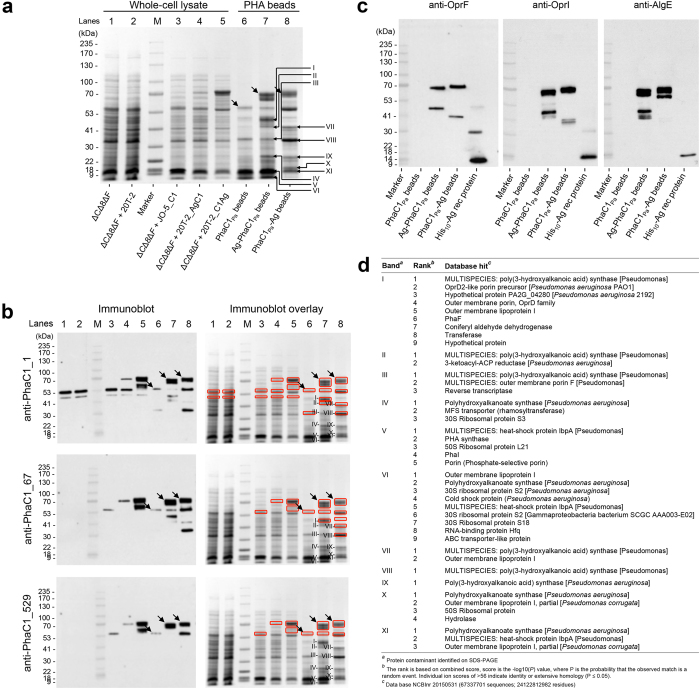
Protein analysis of vaccine PHA_MCL_ beads. (**a**) SDS-PAGE analysis of whole-cell lysate and PHA_MCL_ beads stained with Coomassie Blue or (**b**) probed using anti-PhaC1 polyclonal antibodies raised against various epitopes of PhaC1_Pa_ (see Materials). Fusion proteins of interest in PHA_MCL_ bead samples are indicated by black arrows and the eleven HCP bands identified of interest are indicated in roman numerals. These indicated proteins were isolated for protein identification by MALDI-TOF MS (see Supplementary Tables 1 and 2). Detected bands from **b** immunoblotting were overlaid and matched to specific bands on the Coomassie Blue stained gel (red square). Antibody detected epitopes were aligned with peptides identified by MALDI-TOF MS (see Supplementary Table 3). Major copurified protein bands from PHA_MCL_ beads formed by PHA synthase antigen fusions were identified on SDS-PAGE (see Materials for criteria). (**c**) Immunoblot analysis of isolated PHA_MCL_ beads using polyclonal antibodies raised against epitopes of OprF or OprI or AlgE. (**d**) Table summarizing the Identification of the eleven PHA_MCL_ bead associated HCPs by MALDI-TOF MS (see also Supplementary Table 2). ΔCΔ8ΔF + MCS-5, PAO1 ΔCΔ8ΔF + pBBR1MCS-5; ΔCΔ8ΔF + JO-5_C1, PAO1 ΔCΔ8ΔF + pBBR1JO-5_C1; ΔCΔ8ΔF + JO-5_C1*gfp*, PAO1 ΔCΔ8ΔF + pBBR1JO-5_C1*gfp*.

Whole cell lysates and isolated PHA_MCL_ beads contained dominant proteins with an apparent molecular weight of 62.5 kDa (PhaC1_Pa_), 77.75 kDa (Ag-PhaC1_Pa_) and 79.74 kDa (PhaC1_Pa_-Ag) (Fig. 4a), and their identity was confirmed (Fig. 4b,c and Supplementary Table 1). Densitometry analysis indicated that PhaC1_Pa_, Ag-PhaC1_Pa_, and PhaC1_Pa_-Ag fusion proteins accounted for 8.8%, 12.3%, and 9.9% of total PHA_MCL_ bead associated protein, respectively (data not shown). Several additional copurifying host cell proteins (HCPs) were detected within the bead material (Fig. 4b,c). The major copurifying eleven proteins (Fig. 4a) were selected (labeled I–XI) for identification and peptides belonging to *P. aeruginosa* proteins for each protein band (Fig. 4d and Supplementary Table 2) were ranked based on combined score i.e. –log(*P*) value. Database hits included the PHA synthase, OMPs (i.e. OprI and OprF), ribosomal proteins, naturally bead associated proteins (i.e. PhaI and PhaF), and heat-shock proteins. Some of these OprI, OprF, and AlgE related protein bands were additionally confirmed by immunoblot analysis (Fig. 4c). Interestingly, tryptic peptides of the PhaC_Pa_ were identified in the majority of protein bands as the best hit (Fig. 4d, bands I–IV and VII–XI) and second best hits (Fig. 4d, bands V–VI). Identified PhaC1_Pa_ peptides suggested some degradation from the C terminus. Immunoblot analysis using the antibody anti-PhaC1_1 detected co-purifying protein bands I, II, VII, and VIII, while anti-PhaC1_67 detected protein bands I, VII, and VIII indicating some degradation of the fusion protein.

Antibody detected epitopes were aligned with peptides identified by MALDI-TOF MS for each band and showed that anti-PhaC1_1 recognized epitopes exhibited a coverage of 22% for bands I, II and VII and 71% coverage for band VIII, while anti-PhaC1_67 showed full epitope coverage for bands I and VIII and partial coverage of 17% for band VII (Supplementary Table 3). Alignment of the anti-PhaC1_529 recognized epitope with peptides identified by MALDI-TOF MS in the eleven bands showed that the respective epitope was absent.

The recombinant soluble fusion antigen was produced and purified as shown in Supplementary Fig. 3.

### Immunological response to vaccination with antigen-displaying PHA_MCL_ beads

**Figure 5:**
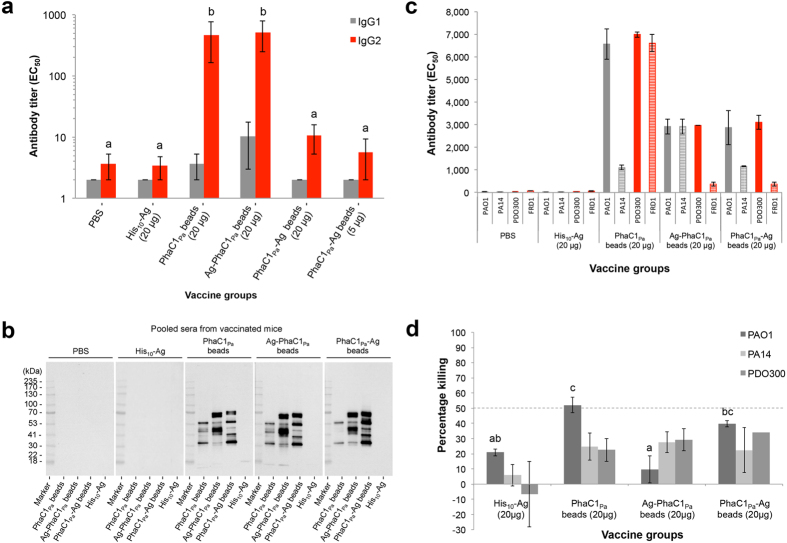
Antibody response to vaccination with vaccine PHA_MCL_ beads. (**a**) Antigen-specific IgG1 or IgG2c isotype antibody responses measured by ELISA using a pool of OprI, OprF, and AlgE antigen specific peptides from sera. Results are expressed in reciprocal antibody titers, representing the dilution required to obtain half of the maximal amount of the OD signal (EC_50_). (**b**) To identify antigenic proteins on PHA_MCL_ beads, sera obtained was pooled in to their respective groups and used as a primary antibody for detection of epitopes on PHA_MCL_ beads separated by SDS-PAGE. (**c**) Reactivity of pooled immune sera to different *P. aeruginosa* strains using a whole-cell ELISA was used. Nonmucoid (grey bars) and mucoid (red bars) strains of *P. aeruginosa* were tested. (**d**) Opsonic killing of *P. aeruginosa* nonmucoid strains by serum from mice immunized with vaccine PHA_MCL_ beads. Bar represents the mean percent killing of three replicates for PAO1 and PA14 or duplicates for PDO300 relative to sera of the PBS vaccinated control group, and error bars represents the s.e.m. Data of graph for IgG are reported as means ± s.e.m (6 mice per group). Statistical significance (*p* < 0.05) of IgG2c is indicated by ‘letter based’ representation of pairwise comparisons between groups using Tukey’s post-hoc test. IgG1 were not statistically significant. Data of graph for whole-cell ELISA represent means of two replicates of pooled sera ± s.e.m of the replicates. There are insufficient replicates to undertake a statistical analysis. Statistical significance (*p* < 0.05) for opsonic killing assay is indicated by ‘letter based’ representation of pairwise comparisons between groups using Tukey’s post-hoc test. PA14 were not statistically significant. There are insufficient replicates to undertake a statistical analysis for PDO300.

C57/BL/6 mice were vaccinated with 20 μg of PhaC1_Pa_ attached to beads or 20 μg OprI/F-AlgE antigen immobilized to beads (Ag-PhaC1_Pa_ or PhaC1_Pa_-Ag) formulated in saline without alum adjuvant. Following vaccination, no obvious adverse effects were observed in any of the animals, with mice gaining weight in all groups. PHA_MCL_ beads stimulated the generation of OprI/F-AlgE antigen specific IgG2c antibodies, indicating a Th1 dominant response (Fig. 5a).

The greatest response was obtained in groups vaccinated with 20 μg of OprI/F-AlgE antigen immobilized to PhaC1_Pa_ beads or 20 μg PhaC1_Pa_ immobilized to beads. No significant difference, but a positive trend was seen between vaccinated groups receiving vaccine formulated with alum compared to their respective groups formulated without alum (Supplementary Fig. 4a). All beads induced sera antibodies specific for epitopes in bead-associated proteins (HCPs, PhaC_Pa_ and PhaC_Pa_ antigen fusions) while this was not observed in the PBS or soluble antigen His_10_-Ag group (Fig. 5b). Reactivity of sera antibodies to whole cells of different *P. aeruginosa* strains were tested (Fig. 5c). Results suggest strong reactivity of sera antibodies in the bead vaccinated groups to both nonmucoid and mucoid strains of *P. aeruginosa*. The PhaC1_Pa_ vaccinated group showed the strongest overall responses compared to Ag-PhaC1_Pa_ or PhaC1_Pa_-Ag bead vaccinated groups. Minimal responses were seen with PBS and recombinant protein vaccinated groups.

An opsonophagocytic killing assay testing serum antibodies from the vaccinated mice was also conducted (Fig. 5d). Serum antibodies from both PhaC1_Pa_ and PhaC1_Pa_-Ag bead vaccinated groups showed significantly higher killing against strain PAO1 than serum antibodies from the PBS group and only PhaC1Pa vaccinated group showed significantly more killing than soluble antigen from the His_10_-Ag group (*p* < 0.05).

**Figure 6:**
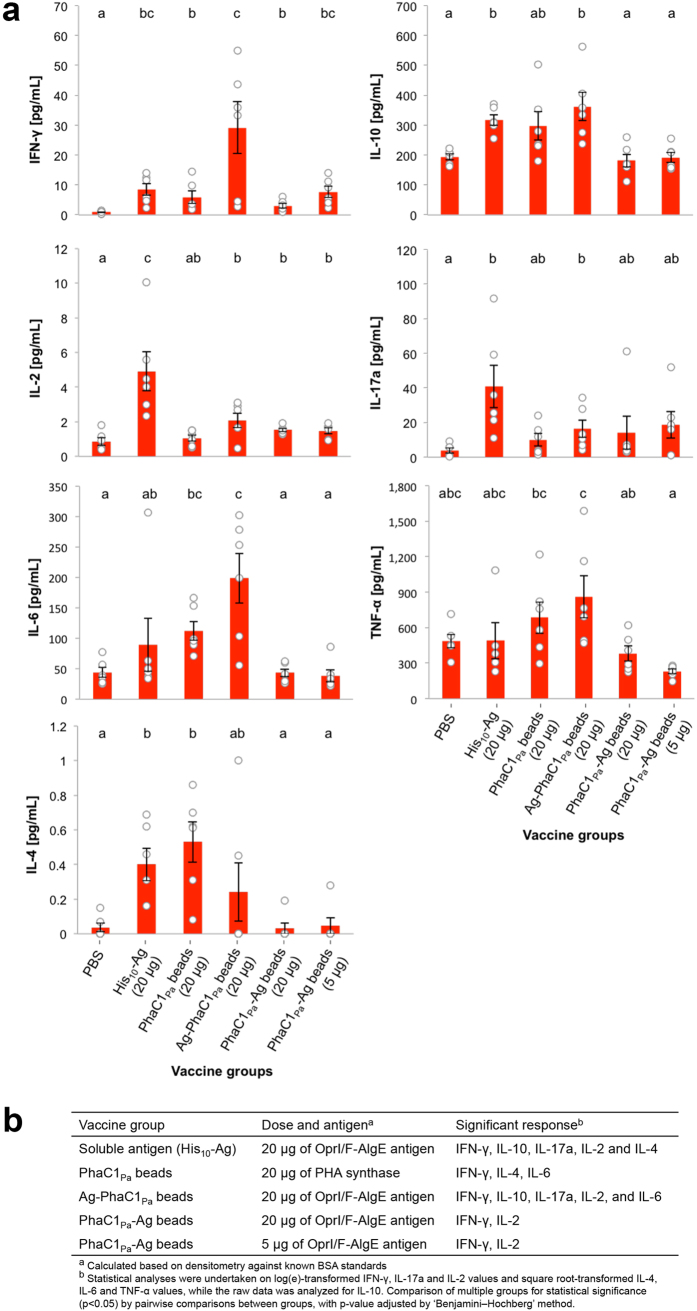
Cytokine response to vaccination with vaccine PHA_MCL_ beads. (**a**) Release of cytokines from splenocyte cultures restimulated with soluble recombinant His_10_-Ag was measured by cytometric bead array. Results are calculated by subtracting cytokine values of the media-stimulated samples from the cytokine values of the recombinant protein stimulated samples. (**b**) Summary of the cytokine response as a comparison to PBS negative control group. Data of graphs are reported as means ± s.e.m and each individual mouse are reported as a dot (n = 6 per group). Statistical significance (*p* < 0.05) is indicated by ‘letter-based’ representation of pairwise comparisons between groups.

Cytokine responses of splenocytes to soluble recombinant protein His_10_-Ag (Fig. 6a,b) showed that Ag-PhaC1_Pa_ beads induced production of significantly more IFN-γ, IL-10, IL-17a, and IL-6 than found in the PBS group, while the PhaC1_Pa_-Ag beads induced significantly more IFN-γ and IL-2 (Fig. 6a,b). Vaccination with 20 μg of PhaC1_Pa_ on beads induced significantly more IFN-γ, IL-4, and IL-6 compared to the PBS group. While the His_10_-Ag group produced significantly more IFN-γ, IL-10, IL-17a, IL-2, and IL-4 compared to the PBS group (Fig. 6a,b). Minimal cytokine responses were observed when splenocytes were re-stimulated with AlgE or OprI or OprF peptides or a combined pool of all peptides (data not shown). Antigens presented on PHA_MCL_ beads did not induce cytokine responses significantly greater than the His_10_-Ag (Fig. 6a). Only the group vaccinated with 20 μg of OprI/F-AlgE antigen on Ag-PhaC1_Pa_ beads produced significantly more IL-6 than the His_10_-Ag group. Notably, a positive trend was observed for cytokines IFN-γ and TNF-α for this Ag-PhaC1_Pa_ beads group and TNF-α for the 20 μg PhaC1_Pa_ beads group when compared with His_10_-Ag group.

Attachment of antigenic proteins to the surface of the beads mediated an enhanced immune response. Significantly more IFN-γ was produced by mice vaccinated with 20 μg OprI/F-AlgE antigen on Ag-PhaC1_Pa_ beads compared with the 20 μg PhaC1_Pa_ beads group (Fig. 6a,b). Although not significant, a positive trend was observed for IFN-γ with the group receiving 20 μg OprI/F-AlgE antigen on PhaC1_Pa_-Ag beads compared to the PhaC1_Pa_ beads group. Comparatively, the N terminal fusion of OprI/F-AlgE antigen to PhaC1_Pa_ on Ag-PhaC1_Pa_ beads induced a greater cytokine response with significantly more IFN-γ, IL-10, IL-6, and TNF-α than its C terminal fusion counterpart, the PhaC1_Pa_-Ag beads. No significant dose response difference was observed in mice receiving either 20 μg or 5 μg of OprI/F-AlgE antigen immobilized on PhaC1_Pa_-Ag beads (Fig. 6a). The addition of adjuvant, alum, generally enhanced the immune response (Supplementary Fig. 4b).

## Discussion

Many bacteria, including animal and human pathogens, are capable of producing spherical discrete PHA inclusions for carbon and energy storage^37^. Here we utilized the intrinsic PHA_MCL_ synthesis capacity of the disease causing pathogen *P. aeruginosa* towards production of antigen-displaying PHA_MCL_ beads. The aim was to display selected repeated epitopes of vaccine candidate antigens of *P. aeruginosa* at high copy number on the surface of the PHA storage granules^5^,^8^,^14^. Design and production of PHA beads in the respective pathogen potentially avoids the need for extensive downstream processing in order to remove host cell derived impurities such as HCPs. Impurities originating from the pathogen could be beneficial, by providing additional epitopes, i.e. a large antigen repertoire, and acting as an adjuvant towards enhanced protective immunity to infections caused by *P. aeruginosa*.

*P. aeruginosa* strain PAO1 was genetically engineered (Fig. 1) by deleting key genes required for the synthesis of PHA, alginate, and pel polysaccharide to enable enhanced recombinant production of its own PHA_MCL_ beads additionally coated with surface epitopes of outer membrane vaccine candidates AlgE, OprF, and OprI as an OprI/F-AlgE fusion antigen^28^,^30^,^38^. This was achieved by protein engineering of the *P. aeruginosa* PhaC1_Pa_ that catalyzes PHA_MCL_ synthesis mediating PHA_MCL_ bead assembly while remaining covalently attached to the surface of PHA_MCL_ inclusions^39^. Various studies have shown great promise for epitopes of OprF and OprI to be used in vaccine formulations^21^,^22^,^34^,^40^,^41^.

The successful recognition and uptake of the vaccine PHA beads by professional antigen presenting cells (APCs) may differ due to the mode of display of the OprI/F-AlgE antigen being presented to immune cells. The tolerance of C terminal fusion was assessed by translational fusion with GFP (Fig. 2a–d), while translational fusion to the N terminus of the PhaC_Pa_ had been previously shown^42^. Hence the OprI/F-AlgE vaccine candidate antigen was fused to either the N or C terminus of PhaC1_Pa_ (Fig. 3a). Translational fusion of the OprI/F-AlgE antigen to the different termini of PhaC1_Pa_ impacted on PHA_MCL_ bead size suggesting an impact of the fusion partner and fusion site on PHA bead assembly (Fig. 3b). Data also showed fusion to the N terminus or C terminus of PhaC1_Pa_ did not influence PHA composition i.e. substrate specificity of PhaC1_Pa_, but reduced PHA_MCL_ accumulation compared to the wildtype (PhaC1_Pa_) implying an impact of the fusion on *in vivo* activity (Fig. 3c). This impact had been found for PhaC_Re_ and was dependent on the fusion partner^10^,^12^,^13^.

Significantly more fusion protein was produced if the OprI/F-AlgE antigen was fused to the C terminus (PhaC1_Pa_-Ag) compared to N terminal fusion (Ag-PhaC1_Pa_) (Fig. 4a). However, the amount of fusion protein did not correlate with the amount of PHA accumulated i.e. PhaC1_Pa_-Ag did not mediate greater levels of PHA_MCL_ accumulation (Fig. 3c).

Successful control of disease caused by many extracellular pathogens typically requires an antibody response characterized by the production of IgG1 isotype and production of cytokines IL-4 and IL-5. However, this might not be the case for chronically *P. aeruginosa* infected CF patients who predominantly show a bias towards a Th2 type immune response when compared to noninfected CF patients or healthy controls^24^,^43^. Elevated levels of antibodies in CF patients tend to be associated with a poor prognosis. There is increasing evidence that a Th1 type immune response characterized by increased cytokine IFN-γ leads to better pulmonary outcomes and may be the preferred response for the successful control of acute and chronically infected CF patients^24^,^26^,^43^.

Here we showed that vaccination of mice with *P. aeruginosa* derived PHA_MCL_ beads displaying the OprI/F-AlgE antigen fused to the N terminus of PhaC1_Pa_ (Ag-PhaC1_Pa_) without adjuvants induced a robust T cell immune response with a Th1 pattern. The immune response was characterized by enhanced production of IgG2c isotype titers and antigen-specific cytokine IFN-γ in association with low levels of IL-4 and IgG1 isotype that are both elevated in the Th2 type response. This suggested induction of a Th1 type response through enhanced CD4^+^ type 1^6^ T cell and Toll-like receptor (TLR) activation^44^.

Vaccination with Ag-PhaC1_Pa_ beads induced significant levels of antigen-specific serum antibodies (Fig. 5a). These antibodies may have resulted from B cell class-switching to IgG2c isotype associated with the activation of Th1 antigen-specific T cells^45^, which conceivably playing a critical role in clearance of acute infection with *P. aeruginosa*^46^.

Vaccination with plain PhaC1_Pa_ beads without OprI/F-AlgE antigen also generated an antigen-specific antibody response to epitopes of the OprI/F-AlgE antigen at levels similar to the Ag-PhaC1_Pa_ bead group (Fig. 5a). This indicated an immune response to the copurified HCPs associated with plain PhaC1_Pa_ beads. For example, the full-length OMP OprF was identified in band III of the separated copurified proteins (Supplementary Table 2). OprI and OprF are present in high copy numbers in the OM of *P. aeruginosa*^47^ and therefore, more likely to be found as part of the HCP impurities compared to the low copy number AlgE^29^. The detection of antibodies against a wide range of copurifying HCPs supported the concept of the immunogenic delivery of a large antigen repertoire using isolated PHA beads produced by the pathogen (Fig. 5b). Moreover, these serum antibodies in the bead vaccinated groups showed strong reactivity across different *P. aeruginosa* strains which include nonmucoid and mucoid variants (Fig. 5c). Opsonophagocytic killing activity of serum antibodies as an indication of protective immunity was also tested (Fig. 5d). Sera antibodies from all bead vaccinated goups could mediate around 20–30% killing to nonmucoid and mucoid variants of *P. aeruginosa*, with lower killing activity seen for soluble His_10_-Ag. The strongest biologically significant killing was mediated by sera antibodies from PhaC1_Pa_ vaccinated group showing opsonophagocytic killing (≥50% killing) against the nonmucoid PAO1 strain, possibly correlating with the strong reactivity of the sera antibodies to antigens of PAO1 (Fig. 5c). Although antigen epitopes were not engineered into the surface of PhaC1_Pa_ beads, evidence was obtained that outer membrane proteins were associated and hence might have contributed to this immune response (Fig. 4). The relatively low killing shown for all vaccine groups may have been due to high MOI of 100:1 in the assay and greater antibody-mediated enhancement of killing may have been seen at a lower MOI.

Alum added to PhaC1_Pa_-Ag beads induced an increase in levels of antigen-specific antibodies (Supplementary Fig. 4a) and a significant increase in some cytokines (Supplementary Fig. 4b) suggesting further scope to enhance immunogenicity of PHA_MCL_ beads.

Attachment of OprI/F-AlgE antigen to the PHA_MCL_ beads enhanced the immune response with a bias towards a Th1 response when compared to vaccination with only antigen. Soluble peptides/proteins require addition of a suitable adjuvant and/or delivery system to generate an optimal immune response^48^. Our results showed that vaccination with His_10_-Ag formulated with alum adjuvant induced mainly a humoral Th2 type response (Supplementary Fig. 4a,b).

Conversely, vaccination with OprI/F-AlgE antigen fused to the C terminus of PhaC1_Pa_ and displayed on PHA_MCL_ beads induced a response similar to plain PHA_MCL_ beads (PhaC1_Pa_), but a weaker response than observed when vaccinating with Ag-PhaC1_Pa_ beads (Figs 5a and 6a). This suggests the OprI/F-AlgE antigen fused to the C terminus of PhaC1_Pa_ may not be fully displayed on the surface of the PHA_MCL_ beads. Due to the inherent orientation of the PHA synthase on the bead surface, the hydrophobic C terminus of PhaC1_Pa_ was proposed to be attached to the hydrophobic PHA core and hence, required a designed linker to enable surface exposure of the fusion partner^10^. The length of the linker may have not been adequate for the full display of the OprI/F-AlgE antigen on the PHA_MCL_ beads surface compared to the N terminal fusion to the PhaC1_Pa_, possibly resulting in reduced OprI/F-AlgE antigen processing by APCs and therefore, leading to a poor antigen-specific immune response^49^.

The reduced immune response seen with PhaC1_Pa_-Ag beads compared to Ag-PhaC1_Pa_ beads could be due to the smaller bead size, resulting in suboptimal antigen uptake compared to the larger Ag-PhaC1_Pa_ beads. Bead size is a major contributing factor influencing particulate antigen uptake by APCs^16^. The mechanism of antigen uptake can influence the type of immune response, inducing humoral and/or cell-mediated immunity. However, the actual size for the most efficient uptake of particulates by APCs is still controversial as efficiency can be affected by a range of other factors including shape, surface charge, hydrophobicity/hydrophilicity and mode of administration^16^. Therefore, it remains unclear if size was a contributing factor for the reduced response to the PhaC1_Pa_-Ag beads. PHA_MCL_ beads produced in this study were all within the generally accepted effective range (<0.5 μm) for uptake by professional APCs and induced an antigen-specific immune response^50^.

Vaccination with Ag-PhaC1_Pa_ beads resulted in significantly increased levels of cytokines IFN-γ, IL-6 and IL-10 with low but significant levels of IL-17a and IL-2 compared to the PBS group suggesting a Th1 and Th17 type immune response (Fig. 6a,b). IL-17a plays a critical role in maintaining control of host defense against extracellular pathogens^51^. Significant level of IL-6 was induced with vaccination using Ag-PhaC1_Pa_ beads, but this did not correlate with high levels of IL-17a. IL-6 together with cytokines IL-1β or TNF-α during acute inflammation can also result in the recruitment of neutrophils^52^. TNF-α levels were elevated but not significantly in mice vaccinated with Ag-PhaC1_Pa_ beads. IL-6 and IL-10 may limit damage in the lungs of CF patients caused by hyper inflammation associated with exacerbated recruitment of neutrophils that lead to pulmonary decline.

**Figure 7:**
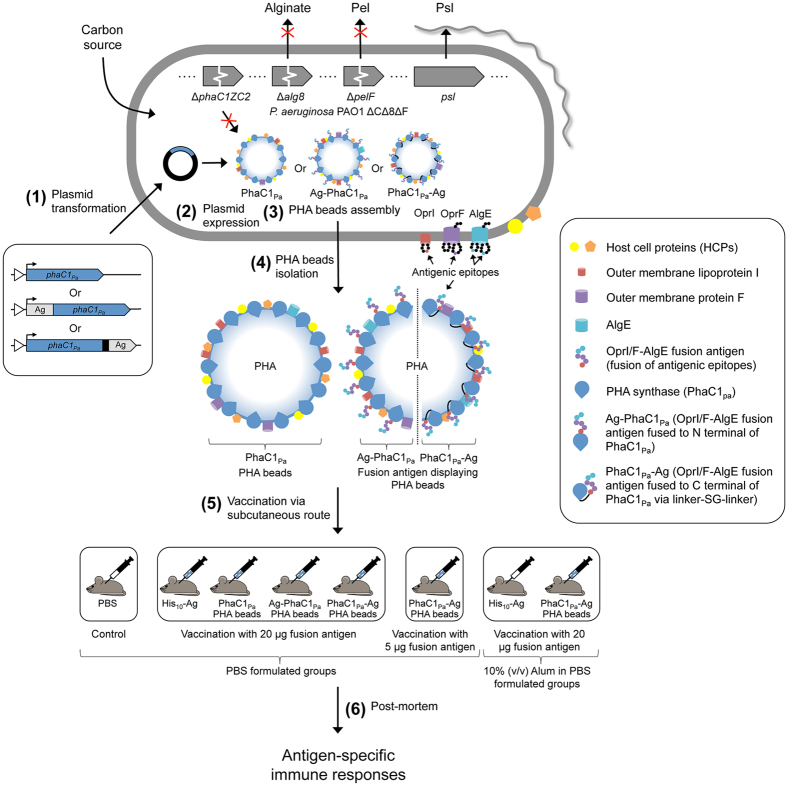
Engineering the pathogens intrinsic ability to produce PHA_MCL_ beads as particulate subunit vaccines. A schematic overview of the production and immunological evaluation of custom-made PHA_MCL_ beads displaying both engineered vaccine candidate antigens and antigens derived from the host expression cells. (**1**) Plasmid encoding wildtype PHA synthase (*phaC1*_*Pa*_) or OprI/F-AlgE fusion antigen fused to the N terminal of PhaC1_Pa_ or OprI/F-AlgE fusion antigen fused to the C terminal of PhaC1_Pa_ via linker-SG-linker were transformed in to *P. aeruginosa* PAO1 ΔCΔ8ΔF mutant strain. This strain is defective in production of native PHA_MCL_ and of EPS alginate and Pel (see Fig. 1). (**2**) Plasmid harboring strains are then grown under PHA_MCL_ accumulating conditions to mediate overproduction of the fusion protein and subsequent PHA_MCL_ bead assembly (See Fig. 3a–c). (**3**) Formation of PHA_MCL_ beads results in the display of fusion antigens covalently linked to the PHA synthase and the incorporation of granule associated and HCPs (See Fig. 4a–d). (**4**) PHA_MCL_ beads are isolated from the host by mechanical disruption and subsequently purified. (**5**) C57BL/6 mice were vaccinated with sterilized PHA_MCL_ beads, recombinant His-tagged OprI/F-AlgE, and PBS via subcutaneous route three times at biweekly intervals. (**6**) Blood and splenocytes were collected from mice euthanized three-weeks after the last vaccination for analysis. Antigen-specific serum antibodies (ELISA) (see Fig. 5a) and cytokines (Cytometric bead array, mouse Th1/Th2/Th17 cytokine kit) (see Fig. 6a,b) were measured.

In conclusion, this study showed that cellular inclusions of bacterial pathogens are immunogenic capable of inducing cell-mediated immune responses. Hence, it is proposed that vaccine research should consider nano-/microsized cellular inclusions as antigen reservoir and delivery system towards the development of safe and efficient particulate vaccines. We proofed the concept of hijacking the capacity of the pathogen *P. aeruginosa* to naturally produce PHA_MCL_ inclusions for the design and production of PHA_MCL_ beads displaying selected antigens of the same host as particulate vaccine candidates (Fig. 7). PHA_MCL_ beads with associated HCPs represented as a large antigenic repertoire. PHA_MCL_ beads displaying vaccine candidates AlgE, OprF and OprI without adjuvant induced a dominant Th1 type response required for the control of *P. aeruginosa* infection^26^,^43^. Since, a range of pathogens such as e.g. *Mycobacterium tuberculosis, Legionella pneumophila*, and *Bacillus anthracis* (Supplementary Fig. 5 and Supplementary Tables 4 and 5), are able to inherently produce PHA inclusions, the demonstrated concept of producing particulate subunit vaccines within the disease causing pathogen represents a novel approach to subunit vaccine development applicable to a range of infectious diseases.

## Methods

### Bacterial strains and growth conditions

All bacterial strains and plasmids used are listed in Supplementary Table 6. *E. coli* strains were grown in Luria broth (LB) medium (Difco, Detroit, MI) at 37 °C unless stated. LB medium was supplemented with 1% NaCl for growth of osmosensitive *E. coli* strain ClearColi (Lucigen, Middleton, WI). When required, antibiotics were used at the following concentrations: ampicillin, 100 μg/mL; and gentamycin 10 μg/mL.

*P. aeruginosa* strains were grown in LB medium (Difco, Detroit, MI) or mineral salt medium (MSM)^53^ at 37 °C and when required, antibiotics were added at the following concentrations: carbenicillin, 300 μg/mL; and gentamycin, 100 to 300 μg/mL.

### Isolation and manipulation of DNA

General cloning procedures were performed as described previously^54^. Electroporation was used for the transfer of plasmid into *P. aeruginosa* strains as described elsewhere^55^. All plasmid isolations were performed using High Pure Plasmid Isolation Kit (Roche, BASEL, Switzerland). DNA primers were purchased from Integrated DNA Technologies (Coralville, IA). *Taq* and platinum *pfx* polymerases were purchased from Invitrogen (Carlsbad, CA). Synthesized peptides and antibodies were purchased from GenScript (Piscataway, NJ). All newly amplified DNA fragments and final plasmid constructs were confirmed by DNA sequencing.

### Construction of alginate- pel- deletion mutant in a PHA negative background

Generation of the isogenic triple mutant (PAO1 ΔCΔ8ΔF) incapable of PHA/alginate/Pel production is outlined in Fig. 1.

The alginate biosynthesis gene *alg8* was disrupted by using the previously described gene-knockout plasmid pEX100T::Δ*alg8*ΩGm^56^. The plasmid was transferred via electroporation into PHA_MCL_ negative *P. aeruginosa* strain PAO1 Δ*phaC1ZC2*^36^ and transformants having undergone the first homologous recombination event were selected on LB medium containing 100 μg/mL of gentamicin. Subsequently, a second homologous recombination event was selected for by plating single cell colonies on LB medium containing 300 μg/mL of gentamicin and 5% (w/v) sucrose. Insertion of FRT-Gm-FRT cassette was confirmed by PCR with primers Alg8_XUP and Alg8_XDN. Gentamicin cassette was removed by the introduction of Flp recombinase-encoding plasmid pFLP2^57^ by electroporation and plated on to LB medium containing carbenicillin (300 μg/mL). Resistant colonies were then screened on LB medium containing 5% (w/v) sucrose. CFU were subsequently screened for gentamicin (300 μg/mL) and carbenicillin (300 μg/mL) sensitivity. PCR with primers Alg8_XUP and Alg8_XDN were used to confirm loss of gentamicin cassette and therefore, *P. aeruginosa* PAO1 Δ*phaC1ZC2* Δ*alg8* double mutant was generated.

Disruption of *pelF* in newly generated strain PAO1 Δ*phaC1ZC2* Δ*alg8* was achieved similarly as described above with the introduction of previously described gene-knockout plasmid pEX100T::Δ*pelF*ΩGm^58^. PCR with primers PelF_XUP and PelF_XDN was used to confirm the insertion and subsequent removal of the gentamicin cassette. Consequently, *P. aeruginosa* PAO1 Δ*phaC1ZC2* Δ*alg8* Δ*pelF* triple mutant was generated, and form now will be referred to as PAO1 ΔCΔ8ΔF. PCR products amplified with primers flanking Δ*alg8* (Alg8_XUP and Alg8_XDN) and Δ*pelF* (PelF_XUP and PelF_XDN) were subsequently used to confirm deletion by DNA sequencing (Supplementary Fig. 1).

### Analysis of the tolerance of translational fusions to the C terminus of the class II PHA synthase

A DNA fragment comprising the Shine-Dalgarno (SD) sequence and gene encoding the class II PHA synthase (*phaC1*_*Pa*_) was excised from pBHR71 with XbaI and BamHI^59^. The fragment was subsequently ligated into the corresponding sites in pBBR1JO-5. Resultant plasmid pBBR1JO-5_C1 constitutively expresses *phaC1*_*Pa*_ in *P. aeruginosa*.

To assess the ability of the class II PHA synthase to tolerate C terminal fusions, a flexible linker extension fusion with the GFP reporter (Linker-SG-linker-*gfp*) used previously to assess C terminal fusion to class I PHA synthase from *Ralstonia eutropha* (PhaC_Re_)^10^ was adapted for use in this study.

The stop codon of *phaC1*_*Pa*_ was removed by PCR amplification using primers F_phaC1 and R_phaC1_(-)stop_BamHI with pBHR71 as template. The amplified PCR fragment encoding SD sequence and *phaC1*_*Pa*_ flanked by sites XbaI and BamHI were ligated into vector pGEM-T easy. Resultant plasmid pGEM-T_C1(-) was hydrolyzed with XbaI and BamHI. Excised DNA fragment was ligated into the corresponding sites in vector pBBR1JO-5 giving intermediate plasmid pBBR1JO-5_C1(-). To generate the corresponding insert, primers F_BgIII_LSGLgfp and R_LSGLgfp_BamHI were used to amplify the DNA sequence encoding the Linker-SG-Linker-*gfp* (LSGL*gfp*) region of pET-14b PhaC-linker-SG-Linker-*gfp*. Resultant fragment flanked with newly introduced BgIII and BamHI sites was ligated into vector pGEM-T easy. Following confirmation, LSGL*gfp* fragment was excised from pGEM-T_LSGL*gfp* with introduced sites and subsequently ligated into the BamHI site in plasmid pBBR1JO-5_C1(-) downstream and in frame of *phaC1*_*Pa*_, resulting in plasmid pBBR1JO-5_C1*gfp*. Ligation resulted in the destruction of the BgIII and BamHI site between *phaC1*_*Pa*_ and linker. Orientation was confirmed by directional PCR.

### Construction of plasmids for the production of OprI/F-AlgE antigen-displaying PHA inclusions

Antigenic epitopes from the outer membrane protein F (OprF_329–342_), mature outer membrane lipoprotein I (OprI_21–83_), and outer membrane porin AlgE (AlgE_233–241_–_287–303_) were combined in a single chain fusion antigen (OprI/F-AlgE) and covalently displayed on the surface of the PHA_MCL_ inclusions. Eptitopes of OprF and OprI (Supplementary Fig. 2a) were selected as previously described^22^,^34^,^38^,^41^,^60^,^61^. Two epitopes, HLRRPGEEV (L5) and NLTTRIATGKQ (L6), corresponding to amino acids 233–241 and 287–303 of AlgE were identified by B-cell epitope prediction method EPCES^62^ (Supplementary Fig. 2b). The OprI/F-AlgE antigen was designed with one copy of AlgE (L5 and L6) and OprI epitopes while including three repeats of the OprF epitope (Fig. 3a). Epitopes in the fusion antigen fragment were arranged as follows: L5-L6-OprF(x3)-OprI for N terminal PhaC1_Pa_ fusion and OprI-OprF(x3)-L6-L5 for C terminal PhaC1_Pa_ fusion. All epitope encoding DNA fragments were synthesized with codon usage bias for *P. aeruginosa* by GenScript (Piscataway, NJ).

An arabinose inducible system (pHERD20T)^63^ was chosen for the expression of genes required for the production of antigen-displaying PHA_MCL_ inclusions in *P. aeruginosa*. Modification of pHERD20T vector was required to remove an alternative start site encoded by *LacZ*. The vector was linearized with NcoI and EcoRI. Resulting cohesive ends of the vector fragment were blunted using T4 DNA polymerase to allow religation, resulting in vector pHERD20T-2.

Generation of the plasmid encoding the N terminal fusion of OprI/F-AlgE antigen to PhaC1_Pa_ was achieved in two steps. Firstly, the DNA fragment encoding *phaC1*_*Pa*_ was excised from plasmid pBBR1JO5_C1 by hydrolysis with XbaI and HindIII and subsequently ligated into the corresponding sites in vector pHERD20T-2. Secondly, resultant plasmid pHERD20T-2_C1 was linearized by hydrolysis with XbaI and NdeI and OprI/F-AlgE antigen fragment excised from pUC57_Ag(N) was successively ligated upstream of *phaC1*_*Pa*_ with corresponding sites, generating the final plasmid pHERD20T-2_AgC1 which encodes for fusion protein Ag-PhaC1_Pa_.

Generation of plasmid encoding C terminal fusion to PhaC1_Pa_ was achieved in a similar fashion to the above. OprI/F-AlgE antigen fragment from pUC57_Ag(C) was excised with SmaI and EcoRI and linear fragment ligated into corresponding sites of plasmid pBBR1JO-5_C1*gfp*, replacing GFP reporter. Newly generated plasmid pBBR1JO-5_C1Ag was hydrolyzed with XbaI and HindIII and resultant linear fragment encoding *phaC1*Ag was ligated into corresponding sites of vector pHERD20T-2, generating final plasmid pHERD20T-2_C1Ag that encodes for fusion protein PhaC1_Pa_-Ag.

### Construction of plasmid pET16b-HisAg for soluble recombinant antigen production

Plasmid pUC57_Ag containing the DNA fragment encoding OprI/F-AlgE antigen fragment with the following arrangement of epitopes OprI-OprF(x3)-L6-L5 synthesized by GenScript with codon usage bias for *E. coli* was hydrolyzed with NdeI and BamHI. The resulting linear OprI/F-AlgE antigen encoding DNA fragment was ligated into the corresponding sites of vector pET16b located downstream and in frame with His_10_-tag resulting in plasmid pET16b-HisAg which encodes for fusion protein Met−Gly−His_10_-OprI_21−83_−(OprF_329**–**342_)_x3_−AlgE_233–241_–_287–303_ (His_10_-Ag) (Fig. 3a).

### Production of PHA inclusions and isolation (beads)

To promote PHA inclusion formation, *P. aeruginosa* strain PAO1 ΔCΔ8ΔF was grown under nitrogen limitation utilizing sodium gluconate as a carbon source^64^. MSM was modified with the following: 0.05% (w/v) NH_4_Cl; and supplemented with 1% (w/v) sodium gluconate^42^. Antibiotics were added at the following concentrations: carbenicillin, 300 μg/mL for strains harboring pHERD20T-2 derivatives; and gentamycin, 300 μg/mL for strains harboring pBBR1MCS-5 derivatives.

A preculture was inoculated from frozen stock and incubated at 37 °C for 10–12 h with agitation. The preculture was then used to inoculate MSM using 5% (v/v) inoculum and grown for further 10–12 h. Main cultures were inoculated with overnight culture giving a starting with OD_600_ of 0.5 and were cultivated at 37 °C with agitation. Induction of main cultures with a final concentration of 0.5% (w/v) arabinose was required when OD_600_ reached 0.4 for PAO1 ΔCΔ8ΔF strains harboring pHERD20T-2 derivatives. PAO1 ΔCΔ8ΔF strains harboring pBBR1MCS-5 derivatives were constitutively expressed in *P. aeruginosa* and did not require induction. All cultures were cultivated for a further 48 h.

Cells were harvested at 4 °C by centrifugation for 10 min at 9,000 × g. The pellet was washed with 100 mL of 50 mM Tris buffer (pH 8) and then again with 50 mL for a total of two washes. Washed cells were then centrifuged at 9,500 × g for 40 min at 4 °C. The pellet was suspended as a 10% slurry (w/v) in lysis buffer (50 mM Tris buffer [pH 8], 50 mM EDTA, 62.5 μg/mL lysozyme) and incubated at 37 °C for 35 min with agitation to digest the cell walls. Cells were then sonicated for 30 sec with a power output of 15 W to sheer DNA prior to mechanical lysis by passing the cell suspension through a French press four times at 6,000 Psi. The crude cell lysate was then sonicated for 30 sec with a power output of 15 W, diluted five times in TE buffer (50 mM Tris buffer [pH 8], 50 mM EDTA) and collected by centrifugation at 9,500 × g for 1 h and 4 °C. The pellet containing crude PHA_MCL_ beads and cell debris was washed with 50 mM Tris buffer (pH 8) and pelleted at 9,500 × g for 30 min and 4 °C and then re-suspended in 20 mL of 50 mM Tris buffer (pH 8) and treated with 0.05 mg/mL DNase + 5 mM MgCl_2_ for 20 min at 4 °C with mixing. Following DNase treatment, the crude PHA_MCL_ bead suspension was sonicated for 30 sec with a power output of 9 W and subsequently washed two times with 50 mM Tris buffer (pH 8), centrifuging for 30 min at 9,500 × g and 4 °C. The PHA_MCL_ bead material was then suspended as 20% slurry (w/v) in 50 mM Tris buffer (pH 8) with 25% glycerol as a cryoprotectant for storage at −80 °C.

### Production and isolation of recombinant protein

*E. coli* strain ClearColi (Lucigen, Middleton, WI) was transformed with pET16b-HisAg and grown in LB miller medium containing 100 μg/mL ampicillin. The main culture was inoculated with overnight culture to give a starting OD_600_ of 0.1 and cultivated at 37 °C. Induction of main culture was achieved with 1 mM IPTG when cultures had reached OD_600_ of 0.3 and further cultivated at a reduced temperature of 30 °C for 15 h with agitation. Cells were harvested at 4 °C by centrifugation for 10 min at 9,000 × g and washed once with 1x PBS (pH 7.4). Sediment The pellet was then suspended as 20% slurry (w/v) in binding buffer (50 mM Tris buffer [pH 7.7], 300 mM NaCl, 10 mM Imidazole). To achieve lysis, cell slurry was sonicated for 10 sec ‘on’ and 10 sec ‘off’ for a total of 10 min ‘on’ at a power setting of 21 W. Crude cell lysate was centrifuged at 9,500 × g for 5 min and supernatant fraction containing soluble protein was filtered through a 0.45 μM pore size filter. Zymo (Irvine, CA) His-Spin Protein Miniprep was used for affinity purification of recombinant His-tagged proteins with the following modifications to the manufacturer’s instructions: 400 μL of filtered cell lysate was mixed with 300 μL of dried His-Affinity Gel per P1 column, and with mixing on a tilting platform left to bind for 5 min. The column was centrifuged at 17,000 × g and sample binding step was repeated several times. Each column was washed twice with 250 μL of wash buffer (50 mM Tris buffer [p H 7.7], 300 mM NaCl, 50 mM Imidazole) and subsequently eluted with 150 μL of elution buffer (50 mM Tris buffer [pH 7.7], 300 mM NaCl, 500 mM Imidazole). The eluted protein was dialyzed against 1x PBS (pH 7.4) at 4 °C overnight using dialysis tubing with 10 K MWCO. Insoluble material was removed by centrifugation at 17,000 × g for 10 min. Recombinant protein His_10_-Ag was sterilized by filtration through a 0.22 μM pore size syringe filter.

### Sterilization of PHA beads

PHA_MCL_ beads were thawed and washed two times using 1x PBS (pH 7.4), centrifuging for 1 h at 14,500 × g and 4 °C. PHA_MCL_ beads were suspended to a 20% slurry (w/v) in 1x PBS (pH 7.4). For sterilization, 1 mg/mL carbenicillin was added to PhaC1_Pa_ PHA beads and 1 mg/mL gentamycin was added to both Ag-PhaC1_Pa_ and PhaC1_Pa_-Ag PHA_MCL_ beads. PHA_MCL_ beads were then distributed into 2 mL screw cap vials and placed in a sonication water bath for 1 h while maintaining a water temperature below 50 °C. Respective PHA_MCL_ bead samples were pooled and washed two times with 1x PBS (pH 7.4), centrifuged at 14,500 × g for 40 min at 4 °C. Beads were suspended as a 10% slurry (w/v) in 1x PBS (pH 7.4) and a representative sample of 200 μl was plated for each group onto LB agar and incubated at 37 °C to check for CFU.

### Nile Red staining and fluorescence microscopy

The presence of PHA_MCL_ inclusions were observed with fluorescent microscopy by staining cells with lipophilic dye Nile Red^65^. MagnaFire imaging software was used to digitally capture images.

### TEM

Transmission electron microscopy (TEM) analysis was used to confirm the accumulation, shape and size of PHA_MCL_ inclusions inside PHA_MCL_ producing recombinant *P. aeruginosa* and respective PHA_MCL_ bead material. Samples were processed for TEM analysis as previously described^66^. Diameters of PHA_MCL_ inclusions in whole-cells was quantified using ImageJ imaging and analysis software (Wayne Rasband) giving approximately 500 data points for each fusion protein.

### Analysis of proteins attached to PHA_MCL_ beads

Sodium dodecyl sulfate-polyacrylamide gel electrophoresis (SDS-PAGE) was used as previously described^67^ to assess the protein profiles of PHA_MCL_ beads and recombinant protein. The gels were strained with Coomassie Brilliant Blue G250. The amount of fusion protein was determined by densitometry against known bovine serum albumin (BSA) standards using Gel Doc XR for detection and Image lab software (version 5.2.1) (Bio-Rad, CA) for analysis. Protein bands of interest were excised from the gels and identified by tryptic peptide fingerprint analysis using matrix-assisted laser desorption-ionisation time-of-flight mass spectrometry (MALDI-TOF MS). The major co-purified protein bands from PHA_MCL_ beads formed by PHA synthase antigen fusions were identified on SDS-PAGE using the following criteria: Image lab software (BioRad, USA) was used for the identification of dominant bands which had a volume threshold of >1,000,000 as analyzed by Image lab software (BioRad, USA) and which were not detected using the specific anti-PhaC1_529 antibody. The anti-PhaC1_529 antibody as opposed to anti-PhaC1_1 and anti-PhaC_67 was specifically detecting the proteins PhaC1_Pa_ (62.5 kDa), Ag-PhaC1_Pa_ (77.8 kDa) and PhaC1_Pa_-Ag (79.7 kDa).

For immunoblot analysis, proteins were separated by SDS-PAGE and transferred to nitrocellulose membranes using an i-BLOT system (Invitrogen, Carlsbad, CA). Membranes were blocked with 2% skim milk in 1x PBS with 0.05% Tween 20 (PBS-T) for 1 h. Following washing with PBS-T, primary antibodies were diluted in 2% BSA and used as follows: For detection of PhaC1_Pa_ (the epitope used for generating anti-PhaC1_Pa_ antibodies: anti-PhaC1_1, MSQKNNNELPKQAA; anti-PhaC1_67, QSELRPGDDDRRFS; and anti-PhaC1_529, RSGKTRKAPASLGN), 0.15–0.2 μg/mL rabbit polyclonal; AlgE (anti-AlgE, and left at room temperature for phase separation. The bottom phase containing HLRRPGEEVC), OprF (anti-OprF, NATAEGRAINRRVEC) and OprI (anti-OprI, SHSKETEARLTATC), 0.1 μg/mL rabbit polyclonal; GFP, 1:4000 dilution rabbit polyclonal (A01388, GenScript, NJ); and specificity of mouse sera antibodies for epitopes in the bead-associated proteins, 1:1500 dilution of pooled mouse serum. After incubation for 1 h, the membrane was washed three times using PBS-T for each 5 min. Secondary antibodies were diluted in 2% BSA and used as follows: anti-mouse HRP at 1:20,000 dilution, and anti-Rabbit HRP at 1:25,000 (Ab6721, Abcam, UK) and incubated for 1 h. After three washes with PBS-T, development was carried out using SuperSignal West Pico chemiluminescent substrate (Thermofisher, Waltham, MA).

### Quantification and analysis of PHA

Typically 10–20 mg of lyophilized cells was subjected to methanolysis as described previously^68^. Methyl esters of the corresponding fatty acid constituents was recovered and analyzed by Gas chromatography-mass spectrometry (GC/MS) for 3-hydroxyalkanoate methyl esters.

### Vaccination of mice

All animal experiments had the approval of the AgResearch Animal Ethics Committee. Female C57BL/6 mice aged 6 to 8 weeks (obtained from the animal breeding facility of AgResearch, Ruakura, Hamilton, New Zealand) were vaccinated three times subcutaneously with 200 μl/injection at 2 week intervals (n = 6 per group) with 20 μg of PHA synthase on PhaC1_Pa_ PHA_MCL_ beads or 20 μg of OprI/F-AlgE fusion antigen on Ag-PhaC1_Pa_ or PhaC1_Pa_-Ag PHA_MCL_ beads or 5 μg OprI/F-AlgE fusion antigen on PhaC1_Pa_-Ag PHA_MCL_ beads (low dose). Additional adjuvant formulated groups were included, 20 μg OprI/F-AlgE fusion antigen on PhaC1_Pa_-Ag PHA_MCL_ beads were mixed with 10% (v/v) alum (A8222, Sigma, MO) or 20 μg soluble recombinant antigen His_10_-Ag protein either alone or mixed with alum to a final concentration of 10% (v/v). PBS-vaccinated control animals were included. Protein concentration was calculated using densitometry and BSA standards.

### Immunological assay

Immunological assays were performed as previously described^14^. Briefly, all mice were anesthetized using a mix of ketamine and xylazine hydrochloride three weeks after last vaccinated. Blood was collected by cardiac puncture, allowed to clot and centrifuged before serum was collected. Spleens were removed and single-cell suspensions were prepared by pushing the samples through a sieve and then repeatedly drawn through a 23 g needle. Suspensions were washed with TAC buffer (17 mM Tris-HCl and 140 mM NH_4_Cl), followed by subsequent washes with PBS. Cells were then cultured at 37 °C and 10% CO_2_ in Dulbecco’s modified Eagle medium supplemented with 2 mM glutamine, 100 U/mL penicillin, 100 μg/mL streptomycin, 5 × 10^−5^ M 2-mercaptoethanol, and 5% (w/v) FCS in 7 well of a flat-bottomed 46-well plate at a concentration of 2 × 10^6^ cells/well in a 1 mL volume. Cells were incubated in medium alone or medium containing 5 μg/mL recombinant protein His_10_-Ag (calculated based on OprI/F-AlgE fusion protein) or 5 μg/mL synthesized peptide (AlgE_233–241_–_287–303_ or OprF_329–342_ or overlapping peptides OprI_21–48, 39_ – _66, 57–83_) or a peptide pool containing a combination of all peptides. 5 μg/mL of Concanavalin A (ConA, Sigma) was used as a positive control.

### Measurement of cytokines

A cytometric bead array (CBA; mouse Th1/Th2/Th17 cytokine kit, BD) was used according to the manufacturer’s instructions to measure interleukin-2 (IL-2), interleukin-4 (IL-4), Interleukin-6 (IL-6), interferon-γ (IFN-γ), tumor Necrosis Factor-α (TNF-α), interleukin-17 (IL-17A), and interleukin-10 (IL-10). Fluorescence was measured using a FACSverse^TM^ flow cytometer (BD) and analyzed using FCAP array software (BD).

### Measurement of serum antibody

Serum antibodies were measured using ELISA. For measuring serum antibodies against recombinant protein, MICROLON 600 (Greiner Bio-One) 96 well flat bottom high binding plates were coated overnight with 5 μg/mL soluble recombinant protein His_10_-Ag or a pool of peptides containing 5 μg/mL of each peptide (AlgE_233–241_–_287–303_, OprF_329**–**342_, OprI_21**–**48, 39_ – _66, 57–83_) in carbonate-bicarbonate buffer (Sigma). Following washing with PBST, plates were blocked for 1 h using PBS-B (1x PBS with 1% (w/v) BSA). After washing with PBS-T, sera was diluted 1:10 and then serially diluted 1:5 in 1x PBS (pH 7.4) were added and incubated for 1 h. After PBS-T wash, secondary IgG1-HRP or IgG2c-HRP (ICL, Newberg, OR) was added at a concentration of 1:5000 and 1:7500, respectively, and incubated for 1 h. Plates were washed and tetramethylbenzidine substrate (BD) was added, color allowed to develop and the reaction was stopped by the addition of 0.5 M H_2_SO_4_ and absorbance read at 450 nM on a VersaMax microplate reader.

Whole-cell EILSA was used to measure reactivity of serum anatibodies to different *P. aeruginosa* strains and were conducted as previously described^69^,^70^. Briefly, *P. aeruginosa* strains were cultivated overnight, cells were washed and diluted to OD_600_ of 0.5 in 1x PBS. Subsequently, resuspended cells were diluted 1:2 and used to coat MICROLON 600 (Greiner Bio-One) 96 well flat bottom high binding plates overnight. Plates were washed with PBS-T and blocked for 1 h using PBS-B. Following washing with PBS-T, plates were blocked for 1 h with serum 1:3 serially diluted in PBS-B starting with a dilution of 1:40. Plates were washed with PBS-T and treated with HRP-conjugated anti–mouse secondary antibodies at a dilution of 1:5000 for 1 h. Plates were washed, OPD substrate (Sigma) added and the reaction was stopped by the addition of 3 M H_2_SO_4_. Absorbance was then read at 490 nm.

### Measurement of opsonophagocytic killing activity of serum antibody

Opsonophagocyticic killing assay was performed as previously described^71^ with some modifications. In brief, assays were performed in 96-well plates using 25 μl of the following assay components: mouse serum diluted 1:2.5 (final concentration 1:10), mouse macrophage RAW 264.7 cells at 2 × 10^6^/ml, *P. aeruginosa* from log-phase culture at 2 × 10^8^/ml, and 4% guinea pig complement as complement (1% final concentration). DMEM medium with 10% heat-inactivated fetal calf serum was used as the diluent. Control reactions, wherein antibody was omitted and substituted with DMEM-10%FCS. Assay was performed at 37 °C with mixing on a plate mixer for 90 mins. Following incubation 25 μl was removed and diluted in deionized water and the serially in saline as described previously^72^, and plated on LB agar for bacteria counts. Plates were incubated overnight at 37 °C. The percent killing was calculated as follows: [1 - (CFU surviving in immune serum at 90 min/CFU surviving in PBS vaccinated serum at 90 min)] × 100.

### Identification of PHA synthases in bacterial human pathogens

eggNOG 4.5^73^, a hierarchical orthology framework with annotations was used for the identification of PHA synthases in bacteria. A sequence search of the database with the amino acid reference sequence of PhaC1_Pa_ from *P. aeruginosa* (X66592.1) identified greater then 359 species of bacteria (e-value ranging from 6.56e-185 to 1.6e-07). 33 human pathogens of interest were identified (Supplementary Table 4).

Homology between the PHA synthases from the selected 33 human pathogens was inferred by multiple alignment of the primary amino acid sequence using T-Coffee^74^ with BLOSUM (Supplementary Fig. 5 and Supplementary Table 5).

### Statistical analysis

ELISA data were analyzed using SoftMax pro 7 and expressed in titers representing the reciprocal of the serum dilution which gave half the maximal optical density (OD) (EC_50_).

For IgG anlaysis, data of graph are reported as means ± s.e.m (6 mice per group). Statistical analyses were undertaken on log(e)-transformed IgG1 and IgG2c values. IgG1 was analyzed by Krustal-Wallis nonparametric test, no significant differences found between the groups. IgG2 was analyzed by one-way ANOVA with statistical significance (*p* < 0.05) indicated by ‘letter based’ representation of pairwise comparisons between groups using Tukey’s post-hoc test.

Analysis of the mean percent kill from triplicate samples of serum were compared by one-way ANOVA with pairwise comparision between groups using Tukey’s post-hoc test (*p* < 0.05).

For analysis of cytokines, results are calculated by subtracting cytokine values of the media-stimulated samples from the cytokine values of the recombinant protein stimulated samples. Data of graphs are reported as means ± s.e.m and each individual mouse are reported as a dot (6 mice per group). Statistical analyses were undertaken on log(e)-transformed IFN-γ, IL-17a, and IL-2 values and square root-transformed IL-4, IL-6, and TNF-α values, while the raw data was analyzed for IL-10. Comparison of multiple groups for statistical significance (*p* < 0.05) is indicated by ‘letter-based’ representation of pairwise comparisons between groups, with *p*-value adjusted by ‘Benjamini–Hochberg’ method.

## Additional Information

**How to cite this article**: Lee, J. W. *et al*. Bioengineering a bacterial pathogen to assemble its own particulate vaccine capable of inducing cellular immunity. *Sci. Rep.* **7**, 41607; doi: 10.1038/srep41607 (2017).

**Publisher's note:** Springer Nature remains neutral with regard to jurisdictional claims in published maps and institutional affiliations.

## Supplementary Material

Supplementary Material
