# Supplementary material for: Bioengineering a bacterial pathogen to assemble its own particulate vaccine capable of inducing cellular immunity
Source: Sci Rep. 2017 Feb 2;7:41607. doi: 10.1038/srep41607 (PMC5288705; doi:10.1038/srep41607)
Supplement: Supplementary Material [file srep41607-s1.pdf]

`

Bioengineering a bacterial pathogen to assemble its own particulate vaccine capable of inducing cellular immunity

Jason W. Lee[1,2], Natalie A. Parlane[2], D. Neil Wedlock[2], and Bernd H. A. Rehm[1,3,4,]*

[1]Institute of Fundamental Sciences, Massey University, Palmerston North, New Zealand; [2]AgResearch, Hopkirk Research Institute, Palmerston North, New Zealand; [3]The MacDiarmid Institute for Advanced Materials and Nanotechnology, Wellington, New Zealand; and [4]PolyBatics, Palmerston North, New Zealand

Supplementary Figures and Tables

Δalg8

Nucleotide sequence:

ATGATGGAAACTTACAAACGTGGCCTCGCCGAAGCCACCGGCTGGCTGGTGTTCCTCAGCCTGCTGATGGT
GCTCGCGCTGGCAGTGCCGAAGACCGTGTTCGACGCCGACTCCAAGGATTTCATCCTGCTTATCGGCGCC
GTCGGCATCTGGCGCTACTCCATGGGCGGCGTGCACTTCCTGCGCGGCATGCAGTTCCTCCACGTGGTCT
ACCCGTACTACCTCCGGCGCGTGCGCCAGTTGGGCAGCGCGGCCGACCCGTCGCACGTGTTCCTGATGGT
CACCAGTTTCCGCATCGACGCCCTGACCACTGCCATGGTCTATCGCTCGGTGATCCGCGAAGCCATCGACA
GCGGCTACCCGACCACCGTGGTCTGCTCCATCGTCGAGATGTCCGACGAGGTCCTGGTCCGTTCTCTGTG
GGAGAAGATGAACC**<>**GGATCCCCGGGTACCGAGCTCGAATTAGCTTCAAAAGCGCTCT**GAAGTTCCTATA
CTTTCTAGAGAATAGGAACTTC**GGAATAGGTACTTCAAGATCCCCAATTCGAGCTCGGTACCCGGGGATCC
G**<>**TCACCATGCTGGTGCTGTTCGACCAGCGCGTCTCGATGTGGACCAGCCTGCTCGGCCTGGTGGTGGC
GATCCTCGCCAGCCTCAAGTACAGCATCGCCTTCCTGCTGGTGTACCTGCTCTGGATCGGCCTCACCCGCC
TGGTGCTGACCCTGCTCCTCTCGCTCTCCGGGCACCGCATCGGCCCGGCCTATCCGCTGATCCTCTATTAC
AACCAGATCGTCGGCGCGCTGGTGAAGATCTACGTGTTCTTCCGCCTCGACCGGCAGTCCTGGACCCGCC
AGCCGACCAAGCTGGAGCGCGGCCTGGCCAGCTTCCAGCGCTGGTTCAACGCCTGGTCGTCGCGGGCCA
TGACCTTCTCTGCCGCCAGCATCTCGTCGCCGTGCTCTATTACAACCAGATCGTCGGCGCGCTGGTGAAGA
TCTACGTGTTCTTCCGCCTCGACCGGCAGTCCTGGACCCGCCAGCCGACCAAGCTGGAGCGCGGCCTGGC
CAGCTTCCAGCGCTGGTTCAACGCCTGGTCGTCGCGGGCCATGACCTTCTCTGCCGCCAGCATCTTCGTC
GCCGTGCTGCTGACCATCGTATGA

ΔpelF

Nucleotide sequence:

ATGACCGAACACACCGCTCCGACGGCGCCCGTCGCCGATGTCTGCCTGCTGCTGGAGGGCACCTGGCCCT
ATGTCCGCGGCGGCGTCTCCAGCTGGGTCAACCAGTTGATCCTCGGTCTCCCCGACCTGACCTTCTCGGT
GTTCTTCATCGGCGGCCAGAAGGATGCCTACGGCAAGCGCCACTACCCGATCCCGGACAATGTGCTGCAC
ATCGAGGAACACTTCCTGGAAACCGCCTGGAGTTCGCCGAACCCGCAGACGCGACAGGGCAGTAGCGAGA
CCGAAAAGGCGTTGCGCGATCTGCACCGTTTCTTCCACTACCCGGAGACGCCGGACGTGGAGGAGGGCGA
CGCGCTGCTCGACCTGCTCGCCGAGGGCCGCATCGGCCGCGAGGACTTTCTCCACAGCA**<>**GGATCCCCG
GGTACCGAGCTCGAATTGGGGATCTTGAAGTACCTATTCC**GAAGTTCCTATTCTCTAGAAAGTATAGGAAC
TTC**AGAGCGCTTTTGAAGCTAATTCGAGCTCGGTACCCGGGGATCC**<>**GTGAAGTTCCTCGGTTTCCGTCG
GATCGGCGAGGTCCTGCCGCAACTCGGCCTGATGGTCCTCACCTCGATCAGCGAAGCGCAGCCGCTGGTG
ATCCTCGAAGCCTGGGCTGCCGGCGCCCCGGTGGTGAGCAGCGACGTCGGCTCCTGCCGCGAACTGATC
GAAGGCGCCGACGCCGAAGATCGCGCCCTGGGTCGCGCCGGGGAGGTGGTGGCGATCGCCGACCCGCA
GGCCACTTCGCGGGCGATCCTCGCCCTGCTGCGCAATCCGCAGCGCTGGCAGGCGGCCCAGGCGGTCGG
CCTGCAACGGGTCGAACGCTACTACACCGAGGCGCTGATGCTCGGACGTTACCGCGGGCTGTACCGCGAA
GCCACGGAGATTGCATGACCATGGCCGGCATCGGCTCCGGGGAGGTGGTGGCGATCGCCGACCCGCAGG
CCACTTCGCGGGCGATCCTCGCCCTGCTGCGCAATCCGCAGCGCTGGCAGGCGGCCCAGGCGGTCGGCC
TGCAACGGGTCGAACGCTACTACACCGAGGCGCTGATGCTCGGACGTTACCGCGGGCTGTACCGCGAAGC
CACGGAGATTGCATGA

Supplementary Figure 1. DNA sequencing results for the generation of *P. aeruginosa* knockout mutant PAO1 ΔCΔ8ΔF. Nucleotide sequences of the truncated genes are displayed and annotated as follows: gene unrelated nucleotides (red), BamHI restriction sites (underlined), and FRT site (Bold).

`

a

OprI — Amino acid sequence:

MNNVLKFSALALAAVLATGCSSHSKETEARLTATEDAAARAQARADEAYRKADEALGAAQKAQQTAD
EANERALRMLEKASRK

OprF — Amino acid sequence:

MKLKNTLGVVIGSLVAASAMNAFAQGQNSVEIEAFGKRYFTDSVRNMKNADLYGGSIGYFLTDDVELA
LSYGEYHDVRGTYETGNKKVHGNLTSLDAIYHFGTPGVGLRPYVSAGLAHQNITNINSDSQGRQQMT
MANIGAGLKYYFTENFFAKASLDGQYGLEKRDNGHQGEWMAGLGVGFNFGGSKAAPAPEPVADVCS
DSDNDGVCDNVDKCPDTPANVTVDANGCPAVAEVVRVQLDVKFDFDKSKVKENSYADIKNLADFMK
QYPSTSTTVEGHTDSVGTDAYNQKLSERRANAVRVNEYGVEGGRVNAVGYGESRPVADNATAEGRA
INRRVEAEVEAEAK

b

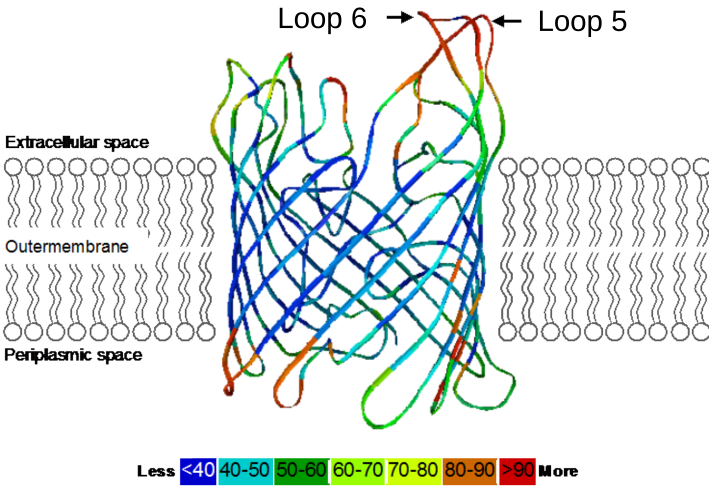

AlgE — Amino acid sequence:

KNFGLDVKITGESENDRDLGTAPGGTLNDIGIDLRPWAFGQWGDWSAYFGQAVAATDTIETDTPDKSY
LAAREFWVDYAGLTAYPGEHLRFGRQRLREDSGQWQDTNIEALNWSFETTLLNAHAGVAQRFSEYRT
DLDELAPEDKDRTHVFGDISTQWAPHHRIGVRIHHADDSG**<L5>**HLRRPGEEV**<L5>**DNLDKTYTGQLT
WLGIEATGDAYNYRSSPLNYWASATWLTGDRD**<L6>**NLTTTRIATGKQ**<L6>**SGDVNAFGVDLGLRWNI
DEQWKAGVGYARGSGGGKDGEEQFQQTGLESNRSNFTGTRSRVHRFGEAFRGELSNLQAATLFGS
WQLREDYDASLVYHKFWRVDDDSDIGTSGINAALQPGEKDIGQELDLVVTKYFKQGLLPASSQYVDEP
SALIRFRGGLFKPGDAYGPGTDSTHRAFVDFIWRF

Supplementary Figure 2. Antigenic epitopes of OprI, OprF, and AlgE. (**a**) The amino acid sequences of OprI and OprF. Selected antigenic epitopes are indicated in red. (**b**) EPCES B-cell epitope prediction of AlgE epitopes. Analysis of chain B in AlgE RCSB Protein Data Bank (3RBH) entry identified extracellular loop 5 (L5) and loop 6 (L6) out of the 9 extracellular loops of AlgE to have high probability of being immunogenic. Predicted antigenic epitopes are illustrated by color, ranging from low (blue) to high (red) probability of being antigenic. The amino acid sequences of selected antigenic epitopes in loops 5 and 6 of AlgE identified by EPCES with >80% probability is indicated in red.

a

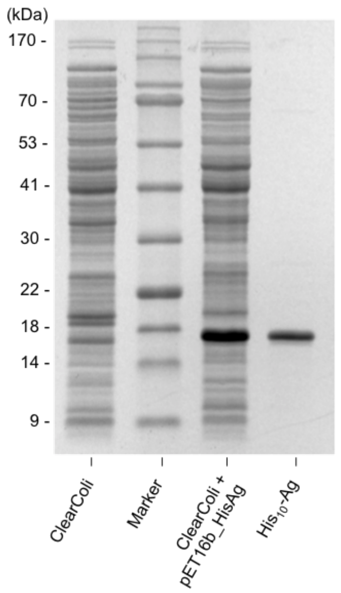

b

His$_{10}$-Ag

Peptide no.	M_r			Miss[a)]	Score[b)]	Expected[c)]	Peptide[d)]
	Observed	Exptl	Calculated				
1	724.3178	723.3105	723.3187	0	37	0.00019	R.ADEAYR.K
4	829.361	828.3537	828.3548	0	34	3.70E-04	R.HMSSHSK.E+Oxidation (M)
8	946.4615	945.4542	945.4516	0	73	7.40E-08	R.VENATAEGR.A
9	973.4897	972.4825	972.4876	0	53	4.80E-06	K.ADEALGAAQK.A
10	1018.5169	1017.5096	1017.5091	0	91	8.00E-10	R.LTATEDAAAR.A
13	1102.5703	1101.563	1101.5527	1	69	1.10E-07	R.RVENATAEGR.A
17	1232.5596	1231.5523	1231.5429	0	102	6.00E-11	K.AQQTADEANER.A
28	1519.7744	1518.7671	1518.7638	1	77	6.40E-08	R.RVENLTTTTVDDR.R
31	1675.866	1674.8587	1674.8649	2	74	3.60E-08	R.RVENLTTTTVDDRR.I

[a)] The number of missed cleavage sites.
[b)] The score is the -log10(P) value, where P is the probability that the observed match is a random event. Individual ion scores of >56 indicate identity or extensive homology (P ≤ 0.05).
[c)] Expected score based on BLAST search.
[d)] The sequence between the peptides was identified by MS. The amino acid before the period at the N terminal and that after the period at the C terminal indicate the cleavage sites.

Supplementary Figure 3. Analysis of soluble recombinant protein His$_{10}$-Ag. His$_{10}$-tagged fusion protein was produced by recombinant ClearColi and subjected to Ni^{2+}-NTA based His-affinity purification. Soluble His$_{10}$-Ag was recovered as 95% pure protein as assessed by (**a**) SDS-PAGE and densitometry (data not shown). (**b**) Protein identification of His$_{10}$-Ag fusion proteins by peptide finger printing using MALDI-TOF MS. (For confirmation by immunoblot analysis see Fig. 4c).

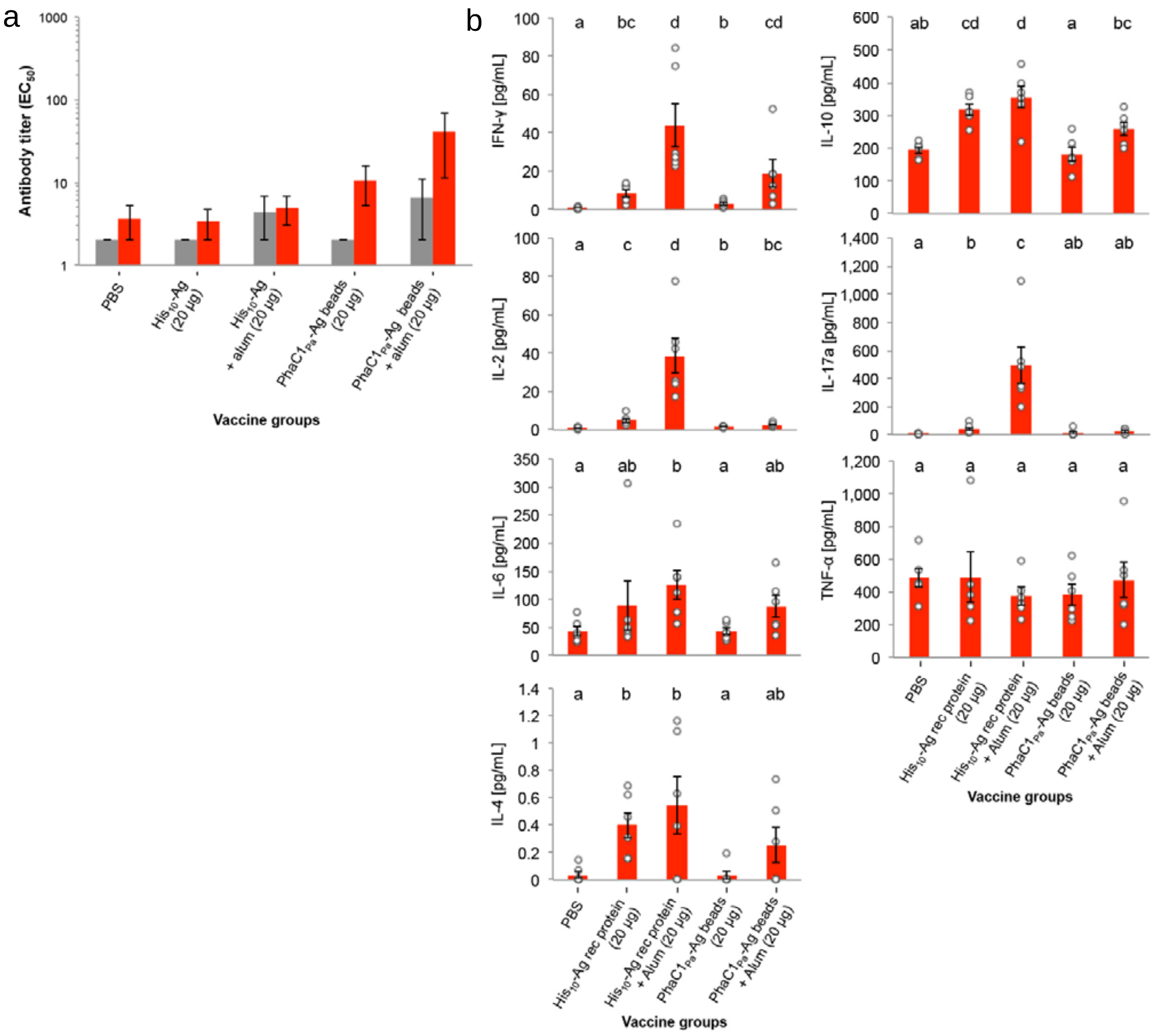

Supplementary Figure 4. Antigenic response to vaccintion with alum formualated vaccine PHA_{MCL} beads. Analysis of antigen-specific antibody and cytokine responses in mice, a comparison of alum and their respective non-alum vaccinated groups. (**a**) Antigen-specific IgG1 or IgG2c isotype antibody responses measured by ELISA using a pool of OprI, OprF, and AlgE antigen specific peptides from sera. Data are reported as means ± s.e.m (6 mice per group). No significance was found. (**b**) Release of cytokines from splenocyte cultures restimulated with soluble recombinant His_{10}-Ag was measured by cytometric bead array. Results are calculated by subtracting cytokine values of the media-stimulated samples from the cytokine values of the recombinant protein stimulated samples. Data of graphs are reported as means ± s.e.m and each individual mouse are reported as a dot (6 mice per group). **S**tatistical significance (p < 0.05) is indicated by 'letter-based' representation of pairwise comparisons between groups**.**

1: *A. baumannii* (HMPREF0010_00690)	MKRLKSLVSEQ-S------------------QI----K------------	---	------------------------HLSTR--------------------	---
2: *A. calcoaceticus* (BDGL_001038)	MKRLKSLVSEQ-S------------------QI----K------------	---	------------------------HLSTR--------------------	---
3: *A. radioresistens* (HMPREF0018_01802)	MFTLKARIRQQKT------------------RF----F------------	---	------------------------HLSRR--------------------	---
4: *B. anthracis* (PHAC)	MTTFATEWEKQ----------LE---------L-----------------	---	-------------------Y-----------------------------	---
5: *B. pertussis* (PHBC)	MNAHLSAAWP--	---	-------------VPV-SVAPDALAEIQADFSREWLRLCDEAK-------	RGVLGA--PADKRFAGAAWLDDRQRLLMAHAYLLSARAMAR-LVEAAQ-V
6: *B. cenocepacia* (PHBC)	MTASKNSSTSAHAGTSAGSTGFDP-AAQPMQQMFESWLNAWRGFADPARA	ATA---SASVNP-FAT-FQFPTSFPFQMPS------MPDFGAMAS-----	-------PFAGLKLPVAAIPPERLQALQADYARDCMTLMQQAA-------	AAKLESPELKDRRFSGDAWKASPAHAFAAAWYLLNARYLQE-LADALQ-T
7: *B. mallei* (PHBC)	MTASKKSSTSSHTDTPQGRSTAGL-AAQPMQQLFESWLGAWRSFADPARA	AAGDAPSPSPSP-FAA-FQPPQPFAFAMPAMPPMPPMPDWSGAAA-----	-------SFAGL-APVASVPPARLQKLQADYSRDCLALIQQAS-------	AATPTVPELKDRRFSADAWKASPAHGFAAAWYLLNARYLQE-LADALE-T
8: *B. mallei* (PHAC)	MDTRHAPESG-----------------AP---------------------	---DAPLPA-HP-PAS-YAPESPYRIF--D------LAKEASVAKLTSGL	SPASLQLALADWLIH-LAAAPGKRAELATLALRHAALLGQYLLEAATGRT	PAAPAQPSPGDRRFRAGAWQLEPYRFW-HQSFLLAEQWWRAATRDVP-GV
9: *B. multivorans* (BMUL_1483)	MTASKNSSTSAAAGTSAGNTGFGS-AAQPMQQMFEAWLNAWRDFADPARA	ATA---SPAVNP-FAS-FQFPKSFPFQMPS------MPDFGAMAS-----	-------PFAGLTLPVAAIPPERLQKLQADYARDCVALMQQAA-------	AAKLEAPELKDRRFSGDAWKASPAHAFAAAWYLLNARYLQE-LADALE-T
10: *B .multivorans* (BMULJ_01759)	MTASKNSSTSAAAGTSAGNTGFGS-AAQPMQQMFEAWLNAWRDFADPARA	ATA---SPAVNP-FAS-FQFPKSFPFQMPS------MPDFGAMAS-----	-------PFAGLTLPVAAIPPERLQKLQADYARDCVALMQQAA-------	AAKLEAPELKDRRFSGDAWKASPAHAFAAAWYLLNARYLQE-LADALE-T
11: *B. pseudomallei* (PHBC)	MQQLFE----------------------------SWLGAWRSFADPARA	AAGDAPSPSPSP-FAA-FQPPQPFAFAMPAM---PPMPDWSGAAA-----	-------SFAGL-APVASVPPARLQKLQADYSRDCLALIQQAS-------	AATPTVPELKDRRFSADAWKASPAHGFAAAWYLLNARYLQE-LADALE-T
12: *B. pseudomallei* (BPSS1954)	MDTRHAPESG-----------------AP---------------------	---DAPLPA-HP-PAS-YAPESPYRIF--D------LAKEASVAKLTSGL	SPASLQLALADWLIH-LAAAPGKRAELATLALRHAALLGQYLLEAATGRT	PAAPAQPSPGDRRFRAGAWQLEPYRFW-HQSFLLAEQWWRAATRDVP-GV
13: *L. pneumophila* (lpg0599)	MLSGARMTHDTE---------LSE----LMQAVAKKSLQIMTDFK----E	K----PIPISSLVSQY-IDLTE---------------------------	-------HFQNL-IAVILKNPEKVWQMQLNYLEDALSLAQAQFNY-----	WLEGKPLPINDQRFNGEDWINNPFFNLLSQHYLLANEHMNS-LLENMEYG
14: *L. pneumophila* (lpg1058)	MSAMKGLEKQ----------------CCPK----KRILTQSQELQ-----	-E-GVDFSC-CA-PADRGTSDNFFPFF--T------RLVQANLAKWTAGI	SPAAIGSSYSTWLWQ-LAQSPGVLWELAFYPVFHAKDCINNIV-------	-CVERAADGKDVRFKKDSWQPMPWRLF-AEGFLQMEDWWRRATTDVP-GL
15: *L. pneumophila* (lpg1097)	MNGLEKQC-------------------CPK----DVILTQQEKAS-----	-E-SLSV-C-CA-PSILGASDSFFSFF--H------KLFQANLAKMTLGI	SPAALGSSYSTWLWQ-LAQSPEVLWELALYPFLHANDCINNII-------	-CVERAADGKDVRFKKDSWQPMPWRLL-AEGFLQVEDWWRR-ATNIP-GL
16: *L. pneumophila* (lpg2260)	MSFMECCYTIFYKSNTYRMQGMKKTNEKPINKTNEMPASSKKEIV-----	----SPL-E-QT-PAS-EQSDPIFRFI--D------KLYQANLGKLTAGI	SPAALGTAYYSWFAQ-LLQSPGSMLRLASYPLLHANDYLSNLF-------	-KYDKPRDGKDVRFHTDNWSYYPWRLW-AEQFLQFEDWCLQASSKVP-GI
17: *L. borgpetersenii* (LBL_2592)	MIAILKNRRSPFY-------------------LATTFF------------	--------ILLF----------------F------ALFASTLAFLFTGI	------------LILILIIHPLLLNW-----------------------	---
18: *L. interrogans* (LA_2034)	MATTFLIL--	----------LL----------------F------ALFASTLAFIFTGV	------------LILILLIHPLLLNW-----------------------	---
19: *M. abscessus* (MAB_2348)	MAFN-ISS----------------------------L------------	-----TKPVARL-AA----------------------------------	-------------------------------------TA-----------	---
20: *M. avium* (MAP1389)	METGT---	---	---	---
21: *M. kansasii* (MKANA1_010100013815)	MAESPKPAAAPDE------------------L-----------------	-----AAPLDL-LLT--SA-----------------------TRPFASR	MMPD-A-TWARLGA-NLAQRPGAVAGRTATLARE---LG----SIAAG--	-KSHRAPGRADKRFGDVAWQQNPLLHRVMQAYLAGAETAEG-LLADAE-L
22: *M. leprae* (ML1346)	MDLN-FSI----------------------------V------------	-----TRPVERL-VA----------------------------------	-------------------------------------TA-----------	---
23: *M. tuberculosis* (MT1723)	MVDLNFSM----------------------------V------------	-----TRPIERL-VA----------------------------------	-------------------------------------TA-----------	---
24: *N. farcinica* (NFA_45720)	MSLAD-TL----------------------------T------------	------L--	-------------------------------------AA-----------	---
25: *P. aeruginosa* (PhaC1)	MSQKNNNEL-PKQ------------------A-----------------	--------AEN-T----LN-----------------------LNPVIGI	RGKDLLTSARMVLL-QAVRQPLHSARHVAHFSLE---LK----NVLLG--	-QSELRPGDDDRRFSDPAWSQNPLYKRYMQTYLAWRKELHS-WISHSD-L
26: *P. aeruginosa* (PhaC2)	MREKQESGSVPVP-------------------------------------	--------AEF-M----SA-----------------------QSAIVGL	RGKDLLTTVRSLAV-HGLRQPLHSARHLVAFGGQ---LG----KVLLG--	-DTLHQPNPQDARFQDPSWRLNPFYRRTLQAYLAWQKQLLA-WIDESN-L
27: *R. equi* (REQ_24810)	MMQVTVSCWGSA---------LVS--------LGSEYV------------	-----VGSLRRA-VA----------------------------------	-------------------------------------TA-----------	---
28: *R. prowazekii* (E.RP820)	MYNITHETIINN---------FKE--------IANKYQELILHFM-QGKG	S----MIPRSL-IDSD-KNIII---------------------------	--------VSCI-IEQFCKNPQKFCQLNIEYIEKLRELTTNSFAKFVGNT	SKDVFSTDNRDKRFKDALWEDNVYFHFVKQYYLLSAEWIKK-NIEQYE-L
29: *R. typhi* (PHBC)	MYNITHETIINN---------FKE--------IANKYQELILHFM-QGKG	S----MIPKSL-IDSD-KNIII---------------------------	--------AYLI-IEQFCKNPQKFCQLNIEYIDKLRELTTNSFAKFVGST	SKDIFYTDNRDKRFKDSLWEDNVYFHFVKQYYLLSAAWIKK-NIEQYE-L
30: *S. rugosus* (HMPREF9336_01483)	MRLAQ-EF----------------------------G------------	------NVAARL-IN----------------------------------	-------------------------------------TA-----------	---
31: *S. maltophilia* (SMAL_2415)	MKGPLGFNADD--------------------L-----------------	---	---	---
32: *V. cholera* (VC_A0688)	MFQHAFTDYLVQ----------------L-QQVNQRW---WKEVE-QSKA	AV---NSPLNKA-MQE-VNLED---------------------------	--------SLKF-FEQAANQPAALLKVQTQWWEQQLQIWQKVV---LESK	IQSIMEAEKGDKRFSHEAWQQDPFFNFIKQSYLLFSKTYLD-TINAIEGL
33: *V. vulnificus* (VV2_0739)	MLQHFFSDYLVK----------------L-QETNQQW---WQDFE-VGKA	AV---NSPLNKA-MQE-VNFED---------------------------	--------TAKF-FEKAANQPQAILQLQTQWWEQQLQIWQNVA---LSGN	TQSIIEADKGDKRFSNEAWQSEAMYSFIKQSYLLFSKTYMD-TIDAIEGL
	*			
CONSENSUS	M			

1: *A. baumannii* (HMPREF0010_00690)	----------------LFRPQTLVL------------------------	----------------------------SQSTPFEVIGEFNQTR-VRYY	AATE-----KSFREPLVFVAPLAINMAIYDLYPYRSLIKYFQNAGFDVYL	VDWGRLGFKDRH-------LNFLSFIEDFIPKAIELVRTHSGSDQISLHG
2: *A. calcoaceticus* (BDGL_001038)	----------------LFRPQTLVL------------------------	----------------------------SQSTPFEVIGEFNQTR-IRYY	AATQ-----KQFKEPLVFVAPLAINMAIYDLYPYRSLIKYFQNAGFDVYL	VDWGRLKFKDRH-------LNFLSFIEDFIPKAIQLVRTHSGSEQISLHG
3: *A. radioresistens* (HMPREF0018_01802)	----------------VLNPESLVL------------------------	----------------------------SQSTPFQVISKYHGSQ-LRYY	AAAH-----KRYKEPLVFVAPLAVDMAIYDLYPYRSLVQHFQLQGFDVYL	VDWGTFTFQDRY-------RDFLFFIDDCLPHYIKTVCEHSQSEKISLHG
4: *B. anthracis* (PHAC)	-----------------------------PEEYR-KAYRRVKRASEIL	LR---------------------EPEPQVGLTPKEVIWTKNKTKLYRYI	PKQE-----KTQRVPILLIYALINKPYIMDLTPGNSLVEYLVDRGFDVYM	LDWGTFGLEDSH-------LKFDDFVFDYIAKAVKKVMRTAKSDEISLLG
5: *B. pertussis* (PHBC)	SEPMRNRLRFSVMQWVDAMSPANFLAFNPDAQRAIVESAGRTLQEGMANL	LNDIQRG----RISQTDETQF---EIGRNVATTPGHVVFENSLMQLIQYA	PQTA-----KVCERPLVIVPPNINKYYILDLQPENSFVRYAVEQGHTVFI	ISWRNPLAADTDG---VDTATWSEYLDDAVLKALAVASDISGQPQVNALG
6: *B. cenocepacia* (PHBC)	DPKTRERIRFTVQQWTAAAAPSNFLALNPDAQKSILETQGESLRQGMMNL	LGDLQRG----KISQTDESQF---VVGKNLGCTEGSVVYENDLIQLIQYT	PKTD-----KVFERPLLIVPPCINKFYILDLQPENSLVAHALSNGHQVFL	VSWRNADASVAH-------KTWDDYMNEGLLAAIDAVQQISGREQINTLG
7: *B. mallei* (PHBC)	DPKTRERIRFTVQQWTAAASPSNFLALNPEAQKNLVETQGESLRLGMMNL	LADMQRG----KISQTDESQF---VVGKNLAVTPGAVVYENDLIQLIQYT	PTTA-----TVFERPLLIVPPCINKFYILDLQPENSLVAHALSCGHQVFL	VSWRNADASVAH-------KTWDDYIDEGLLAAIDVVQQVSGREQINTLG
8: *B. mallei* (PHAC)	SPHHEDVVAFSARQMLDTFAPANYVATNPEIAQRTALTGGANLAQGVWNY	LDDVRRLI--TKQPPAGAEQF---ELGRNLATTPGRVVFRNHLIELLQYS	PTTP-----DVYAQPVLIVPAWIMKYYILDLSAHNSLIRYLVGEGHTVFC	ISWRNVDASDRD-------LSLDDYRKLGVMDALDTIGAIVPGEKIHATG
9: *B. multivorans* (BMUL_1483)	DPKTRERIRFAVQQWTAAAAPSNFLALNPDAQKSILETQGESLRQGMMNL	LGDLQRG----KISQTDESQF---VVGKNLGCTEGAVVYENDLIQLIQYT	PKTA-----KVFERPLLIVPPCINKFYILDLQPENSLVAHAVSSGHQVFL	VSWRNADASVAH-------KTWDDYMNEGLLAAIDAVQQVSGREQINTLG
10: *B .multivorans* (BMULJ_01759)	DPKTRERIRFAVQQWTAAAAPSNFLALNPDAQKSILETQGESLRQGMMNL	LGDLQRG----KISQTDESQF---VVGKNLGCTEGAVVYENDLIQLIQYT	PKTA-----KVFERPLLIVPPCINKFYILDLQPENSLVAHAVSSGHQVFL	VSWRNADASVAH-------KTWDDYMNEGLLAAIDAVQQVSGREQINTLG
11: *B. pseudomallei* (PHBC)	DPKTRERIRFTVQQWTAAASPSNFLALNPEAQKNLVETQGESLRLGMMNL	LADMQRG----KISQTDESQF---VVGKNLAVTPGAVVYENDLIQLIQYT	PTTA-----TVFERPLLIVPPCINKFYILDLQPENSLVAHALSCGHQVFL	VSWRNADASVAH-------KTWDDYIDEGLLAAIDVVQQVSGREQINTLG
12: *B. pseudomallei* (BPSS1954)	SPHHEDVVAFSARQMLDTFAPANYVATNPEIAQRTALTGGANLAQGVWNY	LDDVRRLI--TKQPPAGAEQF---ELGRNLATTPGRVVFRNHLIELLQYS	PTTP-----DVYAQPVLIVPAWIMKYYILDLSAHNSLIRYLVGEGHTVFC	ISWRNVDASDRD-------LSLDDYRKLGVMDALDTIGAIVPGEKIHATG
13: *L. pneumophila* (lpg0599)	DENLAKRVRFFTRQYLDALSPANFIHTNPQLMAETLQSHGKNLLRGLHNL	LSDVEAGSSRLIIKMTDTEAF---KIGENLATTPGKVIFRNSMMELIQYC	PRTT-----KVKSIPLLIVPPWINKYYILDLSPHNSLIRWLVEQGITVFI	ISWVNPDETYAN-------KSFYDYLNEGPREAIAVIQKQLRVKQVNTLG
14: *L. pneumophila* (lpg1058)	PNQVERTVSFWARQCLDALSPSNFVWSNPDLFHEAMRTGGLNLIQGGQIA	LEDWLEKL--TGAPPTGSEHF---IPGKQVAITPGRVVFQNHLIELIQYE	AQTK-----TVYKEPILILPAWIMKYYILDLSPHNSLVKWLVSQGHTVFI	ISWRNPDKEDQD-------LGMDDYYRQGAMAAIDAVSTLFPETKINLMG
15: *L. pneumophila* (lpg1097)	PRQVERTVSFWVRQCLDSVSPSNFVWSNPDLFHEAIRTNGLNLLKGSQIA	MEDWLEKL--TGAPPTGSENF---IPGKDVAVTAGRVVFQNHLIELIQYE	AQTK-----TVYKEPILILPAWIMKYYILDLSPHNSLVKWLVKQGHTVFI	ISWRNPDKEDCD-------LGMDDYYRLGAMAAIDMVSTILPETKINLMG
16: *L. pneumophila* (lpg2260)	PLHVKRTVTFSTRQILDALSPSNFVLTNPDLLQETIRSNGQNLIRGTELA	FQDFVEKI--TGSPPAGVENF---IPGKQVAITKGKVVYSNHLIELIQYT	PQTE-----KVYKEPILILPAWIMKYYILDLLPENSLVNWLVRQGHTVFI	VSWRNPTKEDRN-------LGLDDYYKLGAMDAINAVSNAIPHTKIHLMG
17: *L. borgpetersenii* (LBL_2592)	---	IGKLY---G--QEDIADEVHFAKTKDGW-------------NIALHRHI	PPQQ-----NPQLAPVLVVHGIATNKFMVDLDRRHSLPYYLKLRGYDVFA	VSLRGCGRSYHESPTRYEDFTFDDIVKYDIPAMFEKVKKITGSERVSYVG
18: *L. interrogans* (LA_2034)	---	IGKLY---G--QEDIADEVHFAKTKDGW-------------NLALHRHV	PIQP-----NPQLAPVLVVHGIATNKFVMDLDRRHSLPYYLKLRGYDVFA	VSLRGCGRSYHESPTRYEDFTFDDIVKYDVPAMIEKVKKITGSDRISYVG
19: *M. abscessus* (MAB_2348)	---QNGLEVL	-----------RL-----GGL---ETG--STASSHQIVESVPMYRLRRYF	APGAG---TEDAGPVVLMVHPMMMAADMWDVTQDGGAVGILHRAGIDPWV	IDFGSPDRMAGGM-----ERTLSDHVV-AVSDAIETVHRIT-GRQIHLAG
20: *M. avium* (MAP1389)	---	----------------------------VASPSQIVESVRMYKLRRYF	PPDSRP-GQPPVGPPVLMVHPMMMSADMWDVTRDEGAVGILHAHGLDPWV	IDFGEPDKVEGGM-----RRTLTDHIV-ALSQAIDTVKDVT-GADIHLVG
21: *M. kansasii* (MKANA1_010100013815)	DWRDQEKMQFVVDNLVEGLAPSNNPLISPLGWKALIDTGGLSAVRGLRAF	VRDMLSKPR--VPSMVEPDAF---VVGETVAITKGAVVLQTSMFELIQYT	PQTA-----KVRSIPLLMVPPVINKFYIMDIAPGRSMIEYFLQQGQQVFA	ISWRNPQARHRD-------WGFDAYG-GAIVEAMDAVQNIAGTDSVHLMA
22: *M. leprae* (ML1346)	---QNGLEVL	-----------RL-----GGL---ETG--SFPSPSQIVESVPMYKLRRYF	PPGNRP-GQPLLGAPVLMVHPMMMSADMWDVTREEGAVGILHVRGLDPWV	IDFGSPDKVEGGM-----RRNLADHIV-ALSEAVDTVKEVT-GNDVHLVG
23: *M. tuberculosis* (MT1723)	---QNGLEVL	-----------RL-----GGL---ETG--SVPSPSQIVESVPMYKLRRYF	PPDNRP-GQPPVGPPVLMVHPMMMSADMWDVTREDGAVGILHASGLDPWV	IDFGSPDEVEGGM-----RRNLADHIV-ALSEAVDTVKDAT-GHDVHFVG
24: *N. farcinica* (NFA_45720)	---RNAWALT	-----------FG-----PGV---EAP---EPTRSTVLWDAAHRELRRFE	RDEARDGAAAEGADPVLLVPPLAAPASCFDLRPDQSLARFLLGTGRTPYV	VDYGEITFADRR-------MGFEDWINDILPEAVLRTSADRDGAAVDLVG
25: *P. aeruginosa* (PhaC1)	SPQDISRGQFVINLLTEAMSPTNSL-SNPAAVKRFFETGGKSLLDGLGHL	AKDLVNNGG--MPSQVDMDAF---EVGKNLATTEGAVVFRNDVLELIQYR	PITE-----SVHERPLLVVPPQINKFYVFDLSPDKSLARFCLRNGVQTFI	VSWRNPTKSQRE-------WGLTTYI-EALKEAIEVVLSITGSKDLNLLG
26: *P. aeruginosa* (PhaC2)	DCDDRARARFLVALLSDAVAPSNSL-INPLALKELFNTGGISLLNGVRHL	LEDLVHNGG--MPSQVNKTAF---EIGRNLATTQGAVVFRNEVLELIQYK	PLGE-----RQYAKPLLIVPPQINKYYIFDLSPEKSFVQYALKNNLQVFV	ISWRNPDAQHRE-------WGLSTYV-EALDQAIEVSREITGSRSVNLAG
27: *R. equi* (REQ_24810)	---QNGLEVV	-----------RL-----GGL---ETG--ATPSPFQIVERAPMYRLRRYF	ADTEP----AEAGPPIVLVPPMMMSADVYDVTRDQGAVGILHEMGLDPWV	VDFGSPDAEEGGW-----NRTLADHIV-AISEIVDRVHEHT-GRDVHLSG
28: *R. prowazekii* (E.RP820)	SHDLKQHLEFTTKHFIDAFAPSNFAFCNPKVLRETLESGGHNLVQGLENF	LRDIKSSGDILNINTTDKSAF---KLGQNIAATKGKIIFQNDLMQLICYE	PK-E-----KVHKIPIFIIPPCINKYYILDLSSHNSLVSFLVENNFQVFL	ISWVNPDTSLSK-------KGFEDYLKEGILAPFEYVKNL-GFAKIDFVG
29: *R. typhi* (PHBC)	SHDLKQHLEFTTKHFIDAFSPSNFAFCNPKVLRETLESGGHNLVQGLENF	LRDIKSSGDILNINTTDKSAF---KLGQNIAATKGKIIFQNDLMQLICYE	PK-K-----KVHKIPIFIIPPCINKYYILDLSSHNSLVSFLVENHFQVFL	ISWVNPDTSLSE-------KGFEDYLKEGILAPFEYVKNL-GFAKIDFVG
30: *S. rugosus* (HMPREF9336_01483)	---QNGLEVL	-----------RF-----GGF---DIG--HYESDHQIVDTKPMAQLRRYF	PPGQR---DAPRTAPVLLVPPLGVSADVYDISEHG-AVTQLREAGLDPWV	LDFGSPNRSEREA-----ERSLSDHVL-AVVQAIDLINKHT-KQAPHLAG
31: *S. maltophilia* (SMAL_2415)	------------------------------MQETL-AMQRKLMEGLKLL	PQ---------------------VEDVDYGVTAREEVWRDGKVVLYRFV	GEQA-----PTRRTPLLIVYALVNRPYMVDLQADRSLVQKLLALGQDVYV	LDWGYPDRSERF-------QTLEDYLLRYIDGAVDALRARSG-GPVDMLG
32: *V. cholera* (VC_A0688)	DEKAKERILFFSRQMINALSPSNFIATNPELLRLTLEKNGENLIAGLEQL	KEDVASSADILKIRMTNNNAF---RLGEDVANTPGEVVFKNEVFELIQYK	PLTE-----QVAVTPLLIVPPFINKYYILDLREKNSMVRWLVEQGHSVFM	ISWRNPGAAQAQ-------LNFEDYVLEGVVKAVNAIESITGQEQINAAG
33: *V. vulnificus* (VV2_0739)	DEKTKERISFFSRQAINALSPSNFIATNPELLKLTIEKNGENLLAGMELL	REDVESSADILKIRMTNNNAF---RIGEDIATTAGDIVFQNDLFELIQYR	PLTE-----QVNATPLLIVPPFINKYYILDLTAKNSMVRWLLEQGHSVFM	MSWKNPGKAQAN-------VEFGDYVTEGVVKAVTAIEEITGQEQINAAG
		.	:..: *: :	:. . .
CONSENSUS		G	LLIV DL F	IS I G

Supplementary Figure 5. Multiple alignment of primary structures from 33 known and putative PHA synthase from bacterial human pathogens. Amino sequences were aligned using T-Coffee with BLOSUM. Amino acids are specified by the standard one-letter abbreviations. The consensus sequence represents conserved amino acid sequences. (*), conserved in all sequences; (:), conserved substitutions; (.), semi-conserved substitutions.

1: *A. baumannii* (HMPREF0010_00690)	WSMAGIFVTLYTAHN----HPNYVKNLIVLGSPIDSYASG-YIGKLY-RT	INNT-IA-RNKKLQERIYSGLPKRLIHTPGILNSLGFKILDPKGWF-DGH	IQLLKNLNDLQFVQEHATLSSFLNN--MIDYPG-------------GIN	QDMLFNVWLQNPLRQ-GSIQLKDKKIE-LKNIDCSLLVGAGRSDQLVTAD
2: *A. calcoaceticus* (BDGL_001038)	WSMAGIFVTLYTAHN----HPNYVKNLIVLGSPIDSYASG-YIGKLY-RT	INNA-IG-RNKKIQERIYSGLPKRLIHSPGILNSLGFKILDPKGWL-DGH	IQLLKNLNDLQFVQEHATLSSFLNN--MIDYPG-------------GIN	QDMLFNVWLQNPLKR-GFIELKDKKIE-LKNIDCSLLVGAGRSDQLVTAD
3: *A. radioresistens* (HMPREF0018_01802)	WSMGGIFALLYSALA----KQSHVKNLIILGSPIDSYASG-RIGKLF-KT	VNQL-LT-RHAKIRHSI-ENIPEGLIHTPGFINALGFKIIDPAGWL-NSC	IQLFKYIDNEKFLREHTTVQTFLNH--MNDYPG-------------AIN	KDMIFKVWLKNPLKT-GSIDLKDRLID-LKNIECSLLLGAGTTDQIVTEA
4: *B. anthracis* (PHAC)	YCMGGTLTSIYAALH----PHMPIRNLIFMTSPFDFSETG-LYGPL--LD	EKYF-NL-D-KAVDTF-------GNIPP--EMIDFGNKMLKPITNFVGPY	VALVDRSENERFVESWRLVQKWVGD--GIPFPG-------------ESY	RQWIRDFYQNNKLVK-GELVIRGQKVD-LANIKANVLNISGKRDHIALPC
5: *B. pertussis* (PHBC)	FCVGGTMLASALALAQVRG-ERPVASLTLLTSLLDFHDTG-ILKVF--VD	EAHA-LL-RDHQYGQR-------GLMPA--RDLATTFSFLRPNELV-WNY	V--VSNYLKGKTPPA-FDLLFWNAD--STNLPG-------------PFF	AWYFRNTYLENNLKVPGRARVAGVPLD-LTRLDMPTYLYGSREDHIVPWP
6: *B. cenocepacia* (PHBC)	FCVGGTMLATALAVLAARG-EHPAASMTLLTAMLDFTDTG-ILDVF--VD	EAHV-QM-REQTIGGK--NGAQPGLMRG--VEFANTFSFLRPNDLV-WNY	V--VDNYLKGRTPAP-FDLLYWNSD--STSLPG-------------PMY	AWYLRHTYLENKLREPGALTVCGESVD-LSLIDVPTFIYGSREDHIVPWQ
7: *B. mallei* (PHBC)	FCVGGTMLATALAVLAARG-EHPAASMTLLTSMLDFSDTG-ILDVF--VD	EAHV-QM-REQTIGGK--GGAPAGLMRG--VEFANTFSFLRPNDLV-WNY	V--VDNYLKGRTPAP-FDLLYWNGD--STSLPG-------------PMY	AWYLRNTYLENKLREPDALTVCGEPVD-LSRIDVPTFIYGSREDHIVPWQ
8: *B. mallei* (PHAC)	YCLGGTLLSIAAAAMANTG-DDRLASITLLAAQTDFAEPG-ELQLF--ID	DSEI-HF-LESMMWER-------GYLGA--HQMAGSFQLLMSNDLI-WSR	V--IHDYLLGERTPM-IDLMAWNAD--STRMPY-------------RMH	SEYLRHLFLDNDLAT-NRYVIDGQTVS-VHNIRAPFFVVGTEHDHIAPWR
9: *B. multivorans* (BMUL_1483)	FCVGGTMLATALAVLAARG-EHPAASMTLLTAMLDFSDTG-VLDVF--VD	EAHV-QM-REQTIGGK--NGTPPGLMRG--VEFANTFSFLRPNDLV-WNY	V--VDNYLKGRTPAP-FDLLYWNSD--STSLPG-------------PMY	AWYLRNTYLENKLREPGALTVCGEPVD-LSRIDVPTFIYGSREDHIVPWQ
10: *B .multivorans* (BMULJ_01759)	FCVGGTMLATALAVLAARG-EHPAASMTLLTAMLDFSDTG-VLDVF--VD	EAHV-QM-REQTIGGK--NGTPPGLMRG--VEFANTFSFLRPNDLV-WNY	V--VDNYLKGRTPAP-FDLLYWNSD--STSLPG-------------PMY	AWYLRNTYLENKLREPGALTVCGEPVD-LSRIDVPTFIYGSREDHIVPWQ
11: *B. pseudomallei* (PHBC)	FCVGGTMLATALAVLAARG-EHPAASMTLLTSMLDFSDTG-ILDVF--VD	EAHV-QM-REQTIGGK--GGAPAGLMRG--VEFANTFSFLRPNDLV-WNY	V--VDNYLKGRTPAP-FDLLYWNGD--STSLPG-------------PMY	AWYLRNTYLENKLREPDALTVCGEPVD-LSRIDVPTFIYGSREDHIVPWQ
12: *B. pseudomallei* (BPSS1954)	YCLGGTLLSIAAAAMANTG-DDRLASITLLAAQTDFAEPG-ELQLF--ID	DSEI-HF-LESMMWER-------GYLGA--HQMAGSFQLLMSNDLI-WSR	V--IHDYLLGERTPM-IDLMAWNAD--STRMPY-------------RMH	SEYLRHLFLDNDLAT-NRYVIDGQTVS-VHNIRAPFFVVGTEHDHIAPWR
13: *L. pneumophila* (lpg0599)	FCIGGTLLASLLAYNKATK-DHSIRSATFLAAMIDFSDPG-DIAVF--ID	EQQI-NK-LEEEMKSK-------GYLAG--KFMASSFNSLRANDLI-WSF	F--IKNYLRGKSPVP-FDILYWNAD--STNMPA-------------TMH	SQYLRWMYLHNNLIKPGKIRLNHIPID-VTNIDIPTFFLSTQKDHIAPWK
14: *L. pneumophila* (lpg1058)	YCLGGTLAMITAAAMGRDK-DERLNSLTLLAAQGDFTEAG-ELMLF--VT	ESQV-DF-LKSMMREQ-------GYLDT--KQMAGSFQMLRAYDLI-WSK	M--VQDYMHGMRRGM-IDLTAWNAD--ATRMPY-------------KMH	SEYLEKLFLRNDFAE-GRYTVEGKPVA-AENIKLPVFAVSTEKDHVAPWQ
15: *L. pneumophila* (lpg1097)	YCLGGTLAMITASMMGRYE-DERLNCLTLLAAQGDFSEAG-ELMLF--VN	ESQV-DF-LKSMMWEQ-------GYLDT--KQMAGSFQMLRAYDLI-WSK	M--VQDYMHGMRRGM-IDLTAWNAD--ATRMPY-------------KMH	SEYLEKLFLNNDFAE-GRYMVEGKSVA-AENIQCPIFAVSTKKDHVAPWQ
16: *L. pneumophila* (lpg2260)	YCLGGTLALLTAAAMAHDH-DNRLKTLSLLAAQGDFIDAG-ELLLF--IT	KSEV-SF-LKSMMWEQ-------GYLDT--KQMSGTFQMLRSYDLI-WSK	M--VQDYMHGTQRGM-IPLLAWNAD--ATRMPY-------------KMH	SEYLEKLFLNNDFAE-GRFILDGKPVV-GENIRIPAFVVSTEKDHVAPWK
17: *L. borgpetersenii* (LBL_2592)	HSMGAMILYSHFCMSERKKDTEDIAAFVSLGGPGNLNHIG-IT-------	--LI-GL-LSRFPRAR-------KML----------------DLK-FGA	S--ILAPLAGELYTP-IDEILYNPK--VTSSKTVKKIMKNAIENIADGVT	EQ-FMH-WIETKRMH--SLNGFYDYIRLQKNISVPALFIAGEKDVIATPE
18: *L. interrogans* (LA_2034)	HSMGAMILYSHFCISEHKKDVEDIAAFVSLGGPGNLNHIG-IT-------	--LI-GM-LSRFPRAR-------KML----------------DLK-FGA	S--ILAPLAGELYTP-IDEVLYNPN--TTSSRTVKKIMKNAIENVSDGVT	EQ-FMH-WIETKRMH--SLNGFYDYVQLQKKISVPSLFIAGEKDVIATPE
19: *M. abscessus* (MAB_2348)	YSQGGMFCYQTAALR----KSRTIASIITFGSPVDANAAM-PMGMPAGLS	ADIAE--FMADHVFSR--FSIPA-------WSARIGFQMLDPVKTI-KGR	LDFLRQLHDRDALLPREQQRKFLANEGWIAWSG-------------PAI	AELLRQFVVHNRMTT-GGFTVNDRVVT-LSDITCPVLAVVGEVDDIGQPA
20: *M. avium* (MAP1389)	YSQGGMWCYQVAAYR----RSKSLASIVTFGSPVDTLAAL-PMGIPANFA	APAAN--FMADHVFSR--LAIPS-------WMARTGFQMLDPLKTA-KAR	VDFLRQLHDREALLPREQQRRFLEREGWIAWSG-------------PAI	SELLKQFIAHNRMMT-GGFAVNGQMVT-LTDITCPVLAFVGEVDDIGQPA
21: *M. kansasii* (MKANA1_010100013815)	SCSGGIIAAMTAAHLAHIGEADRVAGLTLAVTVLDETRAG-FAAAA--MS	DRAA-QT-AIRVSARK-------GYLDG--RDMAEMFAWLRPTDLV-WRY	W--VNNYVQGRKPAA-FDVLFWNAD--TTRMAA-------------ALH	RDMV-LMGLRNALVTPGAVTMLGSPVD-LADITSDAYVIGGVADHISPWQ
22: *M. leprae* (ML1346)	YSQGGMFCYQAAAYR----RSENIASIVAFGSPVDTLAAL-PMGIPPNFG	VVLAN--FMADHVFNR--LDIPS-------WLARAGFQMLDPLKTV-KAR	VDFVRQLHNREALLSREQQRRFLESEGWIAWSG-------------PAI	SELLKQFIAHNRMMT-GGFAVNGQMVT-LTDITCPVLAFVGEVDDIGQPA
23: *M. tuberculosis* (MT1723)	YSQGGMFCYQAAAYR----RSKDIASVVAFGSPVDTLAAL-PMGIPANMG	AAVAD--FMADHVFNR--LDIPS-------WMARMGFQMMDPLKTA-KAR	VDFVRQLHDREALLPREQQRRFLESEGWIAWSG-------------PAI	SELLKQFIAHNRMMT-GGFAISGQMVT-LTDITCPILAFVGEVDDIGQPA
24: *N. farcinica* (NFA_45720)	WSLGGTLALLTAAAH----PQLPIGSITAVGSPLDYDRMT-GMPQVRAVA	-KLDGGLAVSTAVRAA--GGIPA-------PLTRAAYRVTAWNREL-TRP	LFVASNIARTEALAKMESIDRFMAQ--MPGYPG-------------RFY	GQLWGRLILNNDIGR-GVLRLGGREIA-LAAVTAPVLLVGGPADVITPAP
25: *P. aeruginosa* (PhaC1)	ACSGGITTATLVGHYVASG-EKKVNAFTQLVSVLDFELNT-QVALF--AD	EKTL-EA-AKRRSYQS-------GVLEG--KDMAKVFAWMRPNDLI-WNY	W--VNNYLLGNQPPA-FDILYWNND--TTRLPA-------------ALH	GEFV-ELFKSNPLNRPGALEVSGTPID-LKQVTCDFYCVAGLNDHITPWE
26: *P. aeruginosa* (PhaC2)	ACAGGLTVAALLGHLQVRRQLRKVSSVTYLVSLLDSQMES-PAMLF--AD	EQTL-ES-SKRRSYQH-------GVLDG--RDMAKVFAWMRPNDLI-WNY	W--VNNYLLGRQPPA-FDILYWNND--NTRLPA-------------AFH	GELL-DLFKHNPLTRPGALEVSGTAVD-LGKVAIDSFHVAGITDHITPWD
27: *R. equi* (REQ_24810)	YSQGGMFCYQAAAYR----RCRNIASLITFGAPVDTLAAL-PFNIPAGLA	TKGAD--LLADHVFNR--LSIAG-------WMARTGFQLLDPVKTA-KSR	FDFLRQLHDRDALLPREQQRRFLAQDGWVAWSG-------------PAV	AELLKQFIVHNRMMT-GGFVIKDTAVS-LAELSCPILAFVGEVDDIGQPL
28: *R. prowazekii* (E.RP820)	YCMGGMFLAIIAYFKVKR-IDSVHSSTFFTTLLDYTNPG-ELGIF--LN	KNTI-NY-IKEEIKLK-------GYFDG--KYLSNSFSLLRANDLI-WTF	F--VNNYLLGKKPMP-FDLLYWNAD--STNLPA-------------KMY	EEYLHNTYCNNLLKESNALEVLGTKID-LGNVDCNSFFLAAKEDHITPWR
29: *R. typhi* (PHBC)	YCMGGMFLAIIAYFKVKS-IDSVHSSTFFTTLLDYTNPG-ELGIF--LN	ENTI-NY-IKEEIKLK-------GYFDG--KYLSNSFSLLRANDLI-WTF	F--VNNYLLGKKPMP-FDLLYWNAD--STNLPA-------------KMY	EEYLHNTYCNNLLKEANALEVLGTKID-LGNIDCNSFFLAAKEDHIAPWR
30: *S. rugosus* (HMPREF9336_01483)	YSQGGMFCYQAAAYR----RSEGIASVITFGSPVDVQAGL-PLGFGGDTG	SDLAE--FLADQVLRR--LAVPK-------WMVRTGFQMMDPVKSV-RAR	VDFLRHLHDRELLLPRERQRRFLMSDGWLATSG-------------PAL	ADFIKAFVVHNRMMT-GGLVINGVPVT-LAEVTCPVLAFVGSYDAIARPP
31: *S. maltophilia* (SMAL_2415)	ICQGGVFALCYAALR----RQ-KLGKLITMVTPVDFHTADNMLSHW--AR	QVDV-DL----LVDTL-------GNIPA--ELMNASYLMLKPFRLNVQKY	VGLLDILDDKAALEDFLRMEKWIFD--SPDLAG-------------EAF	RDFIKQFYQGNGLMY-GTVRIGEEAVD-LSKVTLPVLNIYAEQDHLVPPD
32: *V. cholera* (VC_A0688)	YCIGGTVLATTIAYYAAKRMKKRIKTASFFTTLLDFSQPG-EVGAY--IN	DTII-RA-IELQNNAK-------GYMDG--RSLSVTFSLLRENSLY-WNY	Y--VDNYLKGQSPVD-FDLLYWNSD--STNVAG-------------ACH	NFLLRELYLENKLVQDKGVKVGGVWID-LDKIKVPSYFISTKEDHIALWQ
33: *V. vulnificus* (VV2_0739)	YCIGGTVLACTVGYYAAKRMKKRIKSATFFTTLLDFSQPG-EVGAY--IN	DTII-SA-IELQNNAK-------GYMDG--RSLSVTFSLLRENSLY-WNY	Y--VDNYLKGSSPVD-FDLLYWNSD--STNVAA-------------ATH	NFMLRELYLNNKLVQDKGVKIGGVWID-LDKIRIPSYFISTKEDHIALWQ
	. .. :		:	. : : *:
CONSENSUS	C GG D		W	N V I D I

1: *A. baumannii* (HMPREF0010_00690)	AAQPLSQL-TSSQDVTFTLIP--------GGHLGLMSSQAS---------	---	---	---
2: *A. calcoaceticus* (BDGL_001038)	AAQPLSQL-TSSQDVTFTLIP--------GGHLGLMSSQAS---------	---	---	---
3: *A. radioresistens* (HMPREF0018_01802)	AIQPLSQL-TNSADVSFTAIP--------GGHIGLMSSQAS---------	---	---	---
4: *B. anthracis* (PHAC)	QVEALLDH-ISSTDKQYVCLP--------TGHMSIVYGGTAVKQTY----	---	---	---
5: *B. pertussis* (PHBC)	SAYASTQL-LRG-PMRFVLGA--------SGHIAGVIN------------	---	---	---
6: *B. cenocepacia* (PHBC)	TAYASTSI-LSG-PLKFVLGA--------SGHIAGVIN------------	---	---	---
7: *B. mallei* (PHBC)	TAYASTSL-LTG-PLKFVLGA--------SGHIAGVIN------------	---	---	---
8: *B. mallei* (PHAC)	SVYKIHYL-SGS-DVTFVLTA--------GGHNAGIVS------------	---	---	---
9: *B. multivorans* (BMUL_1483)	TAYASTSI-LTG-PLKFVLGA--------SGHIAGVIN------------	---	---	---
10: *B .multivorans* (BMULJ_01759)	TAYASTSI-LTG-PLKFVLGA--------SGHIAGVIN------------	---	---	---
11: *B. pseudomallei* (PHBC)	TAYASTSL-LTG-PLKFVLGA--------SGHIAGVIN------------	---	---	---
12: *B. pseudomallei* (BPSS1954)	SVYKIHYL-SGS-DVTFVLTA--------GGHNAGIVS------------	---	---	---
13: *L. pneumophila* (lpg0599)	TTYKGFEL-MKG-PKRFVLGG--------SGHIAGIIN------------	---	---	---
14: *L. pneumophila* (lpg1058)	SVYKIHLM-TEG-DVTFVLTG--------GGHNAGIIS------------	---	---	---
15: *L. pneumophila* (lpg1097)	SVYKIHLM-TEE-NVTFVLTG--------GGHNAGIIS------------	---	---	---
16: *L. pneumophila* (lpg2260)	SVYKTHLL-INS-DITFVLTN--------GGHNAGIVS------------	---	---	---
17: *L. borgpetersenii* (LBL_2592)	AVHSVYEN-ASSKKKEFRVISKANGSSDDYGHACLVMGDRAEDD------	---	---	---
18: *L. interrogans* (LA_2034)	SVRSVYEK-ASSRKKEFRVISKANGASDDYGHACLVMGDRAEDD------	---	---	---
19: *M. abscessus* (MAB_2348)	SVRGILR-AAPKADVYEYLIR--------AGHFGLVVGSTAVAQTWPTVS	QWVQWREGE-AAKPPSVDLMY---EHEAGQLDRGGVPLASRVAHGLSTTT	EVAITAARTAGAAAAAANKSVKSIAVEAVRTLPRLTRLGQIHDHTRISMG	RLMTEQARRTPHGECFLFDGRVHTYEAVDRRINNVVKGLIEVGVRQGVRV
20: *M. avium* (MAP1389)	SVRGIRR-AAPDAEVYECTIR--------TGHFGLVVGSKAAQHSWPTVA	AWVKWLSTG-GDKPTGIDPMA---DQPAEHTD-SGVALSSRIAHELGEAS	EAAIGLVRGAANAVVTANKSVRTLAVETARTLPRLVRLGQINDHTRISLG	RIIEEQAHDAPQGEFLLFDGRVHTYEAVNRRINNVVRGLIEVGVRQGDRV
21: *M. kansasii* (MKANA1_010100013815)	ATYRSARL-LGSKDNRYVLST--------SGHIAALVN------------	---	---	---
22: *M. leprae* (ML1346)	SVRGIRR-AAPNAEVYESLIR--------TGHFGLVVGSRAAQQSWPTVA	EWVCWLAAN-ANKPANIHIMP---DQPVEHTA-SGVAISSRLAHGLGEVS	ETALALARGVADAIVAANRSVHTLAVETVRTLPRLARLGQLNDHTRISLG	RIIGEQAHDAPRGEFLLFDGRVHTYEAVDRRVNNVVRGLIAVGVRQGDRV
23: *M. tuberculosis* (MT1723)	SVRGIRR-AAPNSEVYECLIR--------AGHFGLVVGSRAAQQSWPTVA	DWVRWISGD-GTKPENIHLMA---DQPAEHTD-SGVAFSSRVAHGIGEVS	EAALALARGAADAVVAANRSVRTLAVETVRTLPRLARLGQLNDHTRISLG	RIIDEQAHDAPKGEFLLFDGRVHTYEAVNRRINNVVRGLIAVGVRQGDRV
24: *N. farcinica* (NFA_45720)	AVEAGTRTLTGAAFVRYETAP--------GSHLGILTGETARETTWTYLD	EFL--	---	---
25: *P. aeruginosa* (PhaC1)	SCYKSARL-LGG-KCEFILSN--------SGHIQSILN------------	---	---	---
26: *P. aeruginosa* (PhaC2)	AVYRSALL-LGG-QRRFILSN--------SGHIQSILN------------	---	---	---
27: *R. equi* (REQ_24810)	AVRGIKR-AAPRAEVFESTLR--------AGHFGLVVGSAAASRTWPTTG	EWVRWREDM-GPRPEAVDVMI---HDEPSGQD-SGVSLTNRITHTVASIA	EVGVGVGKGIADFATDTVRGTREISIEAARALPRLARLGQLQPHSRISLG	LLLAEQGRQAPNGECFLFDDRVHTNAAVNTRIDNVVRGLVHAGVRPAAHV
28: *R. prowazekii* (E.RP820)	SIYDGVKL-LNG-RKIFCLTD--------SGHVAGVVN------------	---	---	---
29: *R. typhi* (PHBC)	SIYDGVKL-LNG-DKIFCLTD--------SGHVAGVVN------------	---	---	---
30: *S. rugosus* (HMPREF9336_01483)	TVRALPK-VASRAAVWEKEIP--------AGHFGLVVGSLSEREVWPTVA	EWIRWQAGERAGRPEDIEKMAVVAKKAAKKPTQASPGLLGLVQQGAGVLV	EAGVRAGQDALELGVQVGRTATAFAGEGTRAIPLLIRLGQLRPQTRVSLG	LIMAEQARRAPREECFLFADRVHTHEAVGKRVDNVVRGLISVGIRHGDRV
31: *S. maltophilia* (SMAL_2415)	ASRAMRGR-LGTEDYTESSFR--------GGHIGIYVSGRAQREVP----	---	---	---
32: *V. cholera* (VC_A0688)	GTYRGALR-TGG-NKTFVLGE--------SGHIAGIVN------------	---	---	---
33: *V. vulnificus* (VV2_0739)	GTYRGALN-MGG-NKTFVLGE--------SGHIAGIVN------------	---	---	---
	.* .			
CONSENSUS	GH N			

`

Supplementary Figure 5. (*Continued*)

`

1: *A. baumannii* (HMPREF0010_00690)	--	--	--	--
2: *A. calcoaceticus* (BDGL_001038)	--	--	--	--
3: *A. radioresistens* (HMPREF0018_01802)	--	--	--	--
4: *B. anthracis* (PHAC)	--	--	--	--
5: *B. pertussis* (PHBC)	--	--	--	--
6: *B. cenocepacia* (PHBC)	--	--	--	--
7: *B. mallei* (PHBC)	--	--	--	--
8: *B. mallei* (PHAC)	--	--	--	--
9: *B. multivorans* (BMUL_1483)	--	--	--	--
10: *B .multivorans* (BMULJ_01759)	--	--	--	--
11: *B. pseudomallei* (PHBC)	--	--	--	--
12: *B. pseudomallei* (BPSS1954)	--	--	--	--
13: *L. pneumophila* (lpg0599)	--	--	--	--
14: *L. pneumophila* (lpg1058)	--	--	--	--
15: *L. pneumophila* (lpg1097)	--	--	--	--
16: *L. pneumophila* (lpg2260)	--	--	--	--
17: *L. borgpetersenii* (LBL_2592)	--	--	--	--
18: *L. interrogans* (LA_2034)	--	--	--	--
19: *M. abscessus* (MAB_2348)	GILMETRPSALVAIAALSRLGAVAVLLPPDADLEVAVKLGEISELLTDPP	NLPAA----QDLPVHVLVLGGGESRDLSIPDD----GSTIDMEKIDPDAV	ELPGWYRPDPGQARDLAFVMFSGAGSKLLPKQITNHRWALSAFGTASAAA	LSSNDTVYCLTPMHHQSGLLVSIGGSVAGGARIALSRG-----LDPDRFV
20: *M. avium* (MAP1389)	GVLMETRPSALVAIAALSRLGAIAVVMRPDADLAASVRLGGATEILTDPT	NLESVLASDRQLLRQVLVLGGGEARDLHLPEDSAEQPYVIDMEKIDPDAV	ELPGWYRPNPGLARDLAFIAFSAAGGELVAKQITNYRWAVSAFGTASTAA	LDRRDTVYCLTPLHHESALLVSLGGAVVGGTRIALSRG-----LDRDRFV
21: *M. kansasii* (MKANA1_010100013815)	--	--	--	--
22: *M. leprae* (ML1346)	GVLMETRPSALVAIAALSRLGAVAVMMRPDADLAASVRIGGATKILTDPA	NLGVVLAYGRQLTGQVLVLGGGESRDLHLPEDALQQNQVIDMEKIDPNAV	DLPAWYRPNSGLARDLAFIACSTVGGELVAKQITNYRWAVSAFGTASTAA	LDRRDTVYCLTPLHHESALLVSLGGAVVGGARIALSRG-----LCSNRFV
23: *M. tuberculosis* (MT1723)	GVLMETRPSALVAIAALSRLGAVAVVMRPDTDLSASVRLGRVTEILTDPT	NLDAA----RQLPGQVLVLGGGESRDLDLPADALEQGQVIDMEKIDPDAV	ELPAWYRPNPGLARDLAFIAFSSADGDLVAKQITNYRWAVSAFGTASTAA	LGRRDTVYCLTPLHHESALLVSLGGAVVGGTRIALSRG-----LRPDRFV
24: *N. farcinica* (NFA_45720)	--	--	--	--
25: *P. aeruginosa* (PhaC1)	--	--	--	--
26: *P. aeruginosa* (PhaC2)	--	--	--	--
27: *R. equi* (REQ_24810)	GVLMATRPSALVAIAALSRLGAVAVLLPPGGDLDEAVRLGRVDRIVVDPD	HLEDAV----ATGQKVLVLGGGEFRGLAVEL----GPDVIDLEQVDPDAV	TLPGWYRPDPGLARELAFVIFSGSCGNLEAKYITNFRWALSAFGTASAAS	LSRSDTVYCLAPLHHSSGLMATLGGAIAGGARIALSRG-----LDPDRFA
28: *R. prowazekii* (E.RP820)	--	--	--	--
29: *R. typhi* (PHBC)	--	--	--	--
30: *S. rugosus* (HMPREF9336_01483)	GVLMRTRPSALVTIAALNRLGAVAVLIPPWADLRQAVEATGAGAVITDIT	NLEAA----RAAGVTVLVLGVAESRDT--PD---LGPGVVDLEKIDPDKV	SLPGWYTPNPGLAKDLAFILVTNSAGHLVATPMTNQRWAVSAFGTATAAS	LSASDTVYCTSPLHHSAGLVVGWGGAVASGARIALSEYNDVSHNDPERFF
31: *S. maltophilia* (SMAL_2415)	--	--	--	--
32: *V. cholera* (VC_A0688)	--	--	--	--
33: *V. vulnificus* (VV2_0739)	--	--	--	--
CONSENSUS				

1: *A. baumannii* (HMPREF0010_00690)	--	--	--	--------------------------AQEFWPKLATWLSERST-------
2: *A. calcoaceticus* (BDGL_001038)	--	--	--	--------------------------ALEFWPQLAKWLTERST-------
3: *A. radioresistens* (HMPREF0018_01802)	--	--	--	--------------------------ANEFWPKLTEWLVQRSSRIK----
4: *B. anthracis* (PHAC)	--	--	--	-------------------------------PTIGDWLDE----------
5: *B. pertussis* (PHBC)	--	--	------------------------------------PPAKQRRSYWVNES	AGAVSHDLPGDPNAWLAG----AVEHAGSWWPDWTSWLAGHGGKQVAAP-
6: *B. cenocepacia* (PHBC)	--	--	------------------------------------PPAKKKRSYWVNEG	------DLPESADDWFAA----ATEQPGSWWTTWVEWLDAYGGRKVAPP-
7: *B. mallei* (PHBC)	--	--	------------------------------------PPAKRKRSYWSYGA	SA---KELPESANDWLDA----AVEHPGSWWPVWIEWLDQYGGKKVKPR-
8: *B. mallei* (PHAC)	--	--	------------------------------------EPGHAKRHYRMKMT	AA---AAPSISPDEWLAG----ATDFEGSWWPAWHAWLARHSSPQRVAP-
9: *B. multivorans* (BMUL_1483)	--	--	------------------------------------PPAKKKRSYWVNDD	------DLPSAADDWFAG----ATEHPGSWWTTWIEWLDQYGGRKVAAP-
10: *B .multivorans* (BMULJ_01759)	--	--	------------------------------------PPAKKKRSYWVNDD	------DLPSAADDWFAG----ATEHPGSWWTTWIEWLDQYGGRKVAAP-
11: *B. pseudomallei* (PHBC)	--	--	------------------------------------PPAKRKRSYWSYDA	SA---KELPESANDWLDA----AVEHPGSWWPVWIEWLDQYGGKKVKPR-
12: *B. pseudomallei* (BPSS1954)	--	--	------------------------------------EPGHAKRHYRMKMT	AA---AAPSISPDEWLAG----ATDFEGSWWPAWHAWLARHSSPQRVAP-
13: *L. pneumophila* (lpg0599)	--	--	------------------------------------PPTQQKYGYRTNN-	------SMDLSAEQWFEK----SKEHSGSWWPEWLNWLKLHSGRLINSP-
14: *L. pneumophila* (lpg1058)	--	--	------------------------------------EPGHPGRSYRVHEQ	KQ---GEAYLNPESWLAM----AERREGSWWREWNEWLVQQNTKKRIAS-
15: *L. pneumophila* (lpg1097)	--	--	------------------------------------EPGHSGHAYHVHER	KK---GDAYLNPTNWLEI----AEKREGSWWREWHDWLVQQSTKKRILP-
16: *L. pneumophila* (lpg2260)	--	--	------------------------------------EPGHEGRYYRIRER	KM---DSTYLDPTTWVKR----AELREGSWWIAWHDWLVNHSSQKQVSA-
17: *L. borgpetersenii* (LBL_2592)	--	--	--	-----------------------------VFQYVESFLKKHGLRSQ--P-
18: *L. interrogans* (LA_2034)	--	--	--	-----------------------------VFQHVESFLKKHGLRDQ--P-
19: *M. abscessus* (MAB_2348)	QEIHQYGVSVVSYTWAMMHEVIDDPA--LALGAHHPVRLFIGSGMPAGLW	RRVTEKFDPAHVVEFFATTDGEAVLANVSGTKVGSKGRPLPGGGKVRLAA	YDPVEDVIIEGEDGFVQIAEPGEVGLLLAKPPGDVDPTAAVRRGVF----	---------APGDTWVSSEFLFRRDEDGDFWM------LDGRGTAIRTAH
20: *M. avium* (MAP1389)	QEVRQYGVTVVSYTWAMLREIVDDPA--FVLHGNHPVRLFIGSGMPTGLW	GRVVEAFAPAHVVEFFATTDGQAVLANVSGAKVGSKGRPLPGAGRIELGA	YDTEHDLILENDRGFVQIAEPHQVGVLLAASNGPIDPSASVKRGVF----	---------AAGDTWISTEYLFYRDDDGDYWL------AGRRGSVVHTPR
21: *M. kansasii* (MKANA1_010100013815)	--	--	------------------------------------PPGNPKASFRTGL-	------VGAEKPEEWLES----AQQSAGSWWPDYVSWLAERSGPEVDAP-
22: *M. leprae* (ML1346)	HEVRQYGVTVVSYTWAMLRELVDDPA--FVLHGNHPVRLFMGSGMPTGLW	ERVVEAFAPAHVVEFFATVDGQAVLANVSGAKIGSKGRPLPGAGHVELGA	YDAEQDLILENDRGFVQVADVNQIGVLLAASRGPIDPTASVKRGVF----	---------APADTWIATEYLLRRDYDGDYWL------AGRRSSVVRTAR
23: *M. tuberculosis* (MT1723)	AEVRQYGVTVVSYTWAMLRDVVDDPA--FVLHGNHPVRLFIGSGMPTGLW	ERVVEAFAPAHVVEFFATTDGQAVLANVAGAKIGSKGRPLPGAGRVELGA	YDAEHDLILENDRGFVQVAGVNQVGVLLAQSRGPIDPTASVKRGVF----	---------APADTWISTDYLFWRDDDGDYWL------AGGRGSVVRTAR
24: *N. farcinica* (NFA_45720)	--	--	--	--------------------------------------TE----------
25: *P. aeruginosa* (PhaC1)	--	--	------------------------------------PPGNPKARFMTNP-	------ELPAEPKAWLEQ----AGKHADSWWLHWQQWLAERSGKTRKAP-
26: *P. aeruginosa* (PhaC2)	--	--	------------------------------------PPGNPKACYFEND-	------KLSSDPRAWYYD----AKREEGSWWPVWLGWLQERSGELGNPD-
27: *R. equi* (REQ_24810)	EEVRRYGVTVVSYTWTMMREILDAKS--LPLEEGHPIRLFIGSGMPPGLW	RRISSRFAPARVLEFYASTEGDVVLVNVSGAKVGSKGRRLPGSAEVRVGA	YDPIEGRFLEDARGFVRECEDDEVGLLLGRPGARTEAVPGVMRGVF----	---------AAGDAWVPTENLFRRDADGDFWL------VDHKRTVVDTVR
28: *R. prowazekii* (E.RP820)	--	--	------------------------------------HPDNAKYNYRLNY-	------DLSLSSNEWFMQ----ATEYKGSWWNYWIDWLIKNNDTKMLVD-
29: *R. typhi* (PHBC)	--	--	------------------------------------HPASAKYNYRINY-	------DLSLSSNEWFMQ----ATEYKGSWWNYWIDWLIKNNDTKILVD-
30: *S. rugosus* (HMPREF9336_01483)	EEVHRYGVTVVSYTWTQLRPVLAEMEKRRSAEQRLPIRLFVGSGIPAGQW	ERVQEQFAPARVVEFFASVQGGAVLANVRGVKPGSKGRPLPGAARLELGA	YDASADQLVLGQRGFVRRAKPGEPGVLIVKPTLEQES-FLSRRGIF----	---------EQCDEWVITDHIFRRDDDGDFWL------LDNRSALIRAQD
31: *S. maltophilia* (SMAL_2415)	--	--	--	-------------------------------ATIDGWLKA----------
32: *V. cholera* (VC_A0688)	--	--	------------------------------------HPDKRKYGYWVND-	------TLDDSAEDWLET----AQHREGSWWVHWNEWLNGFADGSKVEP-
33: *V. vulnificus* (VV2_0739)	--	--	------------------------------------PPAKNKYGFWVND-	------NLDESADEWLSN----AQHKEGSWWTHWDQWLTQFNPAEKVLP-
CONSENSUS				

Supplementary Figure 5. (*Continued*)

1: *A. baumannii* (HMPREF0010_00690)	--	--	------------------------------------KI
2: *A. calcoaceticus* (BDGL_001038)	--	--	------------------------------------QI
3: *A. radioresistens* (HMPREF0018_01802)	--	--	-----------------------------------DTL
4: *B. anthracis* (PHAC)	--	--	-----------------------------------RSK
5: *B. pertussis* (PHBC)	--	AQAGNKR---	-----------------FRPIEPAPGRYVKV----RAV
6: *B. cenocepacia* (PHBC)	--	AQPGSAQ---	-----------------FPVIEPAPGRYVL-----QRD
7: *B. mallei* (PHBC)	--	AHLGCAR---	-----------------FPVIEPAPGRYVL-----QRD
8: *B. mallei* (PHAC)	--	PPLGKPG---	-----------------AHTLGDAPGTYVF------QK
9: *B. multivorans* (BMUL_1483)	--	AELGSAQ---	-----------------FPVIEPAPGRYVL-----QRD
10: *B .multivorans* (BMULJ_01759)	--	AELGSAQ---	-----------------FPVIEPAPGRYVL-----QRD
11: *B. pseudomallei* (PHBC)	--	AHLGCAR---	-----------------FPVIEPAPGRYVL-----QRD
12: *B. pseudomallei* (BPSS1954)	--	PPLGKPG---	-----------------ARTLGDAPGTYVF------QK
13: *L. pneumophila* (lpg0599)	--	-DINHLP---	-----------------FAPIMDAPGSYVL------KK
14: *L. pneumophila* (lpg1058)	--	SVMN--	------------------PSLPEAPGTYVL------QK
15: *L. pneumophila* (lpg1097)	--	PVLN--	------------------TSLPSAPGTYVL------QK
16: *L. pneumophila* (lpg2260)	--	PKLD--	------------------KKLPNAPGKYVL------QK
17: *L. borgpetersenii* (LBL_2592)	--	-GIMTKI---	-----------------KEG------ILS-----AFRR
18: *L. interrogans* (LA_2034)	--	-GIGTKI---	-----------------KEGILS---ALF-----RFRG
19: *M. abscessus* (MAB_2348)	GVVYAEATSNALGALGAIDLVATYPVETGETTVAVTAVVLRPGEALSPAD	LAEAFAAVAISERPDIIKVVPNLPLSASYRPSTTHLRASGLPKPGRQTWH	LDPESGAYHRLTAATYEA----------LR-----GAVL
20: *M. avium* (MAP1389)	GVVYAEPVTDALGCINGVDLAVTYNVPVGGHEVAVSAVTLLPGASITAAD	LTEACAKIPIGLGPDIVCVVPEMNLSATYRPTVSALRAAGIPKAGRQVWY	FDAESGQYRRLTPAARAE----------LS-----GGRS
21: *M. kansasii* (MKANA1_010100013815)	--	KALGGQG---	-----------------LPPLGPAPGTYVK------EQ
22: *M. leprae* (ML1346)	GLVYTEPVTDALGFITGVDLAATYSVAVDDRELAVSAVTLLPGAAITAAD	LTEAVASMPVGLGPDIVHVVPELTLSATYRPIVGALRTAGIPKTGRQVWY	FDSASNQFRRMTPGVRAE------LAGKHT-----HTHA
23: *M. tuberculosis* (MT1723)	GMVYTEPVTNALGLITGVDLAVTYGVLVRGRHVAVSAVTLLPGATITAAD	LTEAVASMPVGLGPDIVHVVPQLTLSGTYRPTVSALRANGIPKAGRQAWY	FNSGGNEYRRLTPAVRTE------LTGQHR-----RGNA
24: *N. farcinica* (NFA_45720)	--	--	-----------------------AAAV-R-----ESVS
25: *P. aeruginosa* (PhaC1)	--	ASLGNKT---	-----------------YPAGEAAPGTYVH------ER
26: *P. aeruginosa* (PhaC2)	--	FNLGSAA---	-----------------HPPLEAAPGTYVH------IR
27: *R. equi* (REQ_24810)	GPVFTQPIVDLLGEMPQVDLAVAYGVPTGEHQVPVAAITVHDGRVPSAAE	VTAALGQLPIEQCPDIVHVVDRIPLGPSYRPQATELQAAGLPKPSARSWC	LDAETGRYKRFTKAAAARYGGSPEGPG--------GVPA
28: *R. prowazekii* (E.RP820)	--	-SLDYQN---	-----------------LDVIESAPGSYV-------RR
29: *R. typhi* (PHBC)	--	-SLDYQN---	-----------------LDVIEIAPGSYV-------RR
30: *S. rugosus* (HMPREF9336_01483)	GPIWSQPILDALDRIPAVDLAVVYRTQTAGQELAVAAVTLRPGARLRATD	LQLGLGPVPASERPHLVRVVPEIPLSPTYRPIGYKLQADGTPRPGRGVWC	RD-EDGEYAPFTAAK------------AR-----SLGW
31: *S. maltophilia* (SMAL_2415)	--	--	-----------------------------------RDA
32: *V. cholera* (VC_A0688)	--	YPLGNAD---	-----------------YPVLYSAPGEYVKQVLPIQEA
33: *V. vulnificus* (VV2_0739)	--	YRQGSEA---	-----------------NPVIDIAPGQYVKQVL-PITE
CONSENSUS			

Supplementary Figure 5. (*Continued*)

Supplementary Table 1. Protein identification of fusion proteins by peptide finger printing using MALDI-TOF MS

PhaC1$_{Pa}$

Peptide no.	M_r Observed	Exptl	Calculated	Miss[a)]	Score[b)]	Expected[c)]	Peptide[d)]
4	960.5614	959.5541	959.54	1	20	0.0094	R.GKDLLTSAR.M
6	1123.6532	1122.6459	1122.6397	0	45	3.30E-05	K.SLLDGLGHLAK.D
7	1180.6414	1179.6341	1179.64	0	55	2.90E-06	R.HVAHFSLELK.N
8	1230.6066	1229.5993	1229.5968	0	51	8.00E-06	K.FYVFDLSPDK.S
11	1277.6785	1276.6712	1276.6776	0	58	1.60E-06	K.NLATTEGAVVFR.N
12	1306.6865	1305.6792	1305.683	0	71	7.90E-08	R.NGVQTFIVSWR.N
15	1375.6666	1374.6593	1374.6754	1	23	0.0055	R.YMQTYLAWRK.E + Oxidation (M)
17	1552.7399	1551.7326	1551.7358	0	54	3.60E-06	R.FSDPAWSQNPLYK.R
18	1570.84	1569.8327	1569.8555	0	114	3.80E-12	R.LPAALHGEFVELFK.S
21	1657.8287	1656.8214	1656.8512	1	77	2.00E-08	K.FYVFDLSPDKSLAR.F
22	1708.8257	1707.8184	1707.8369	1	54	3.80E-06	R.FSDPAWSQNPLYKR.Y
23	1722.9229	1721.9156	1721.9424	0	81	2.50E-08	K.QAAENTLNLNPVIGIR.G
25	1746.9027	1745.8954	1745.9213	1	51	7.70E-06	R.NGVQTFIVSWRNPTK.S
26	2078.094	2077.0867	2077.1167	0	128	1.50E-13	K.SNPLNRPGALEVSGTPIDLK.Q
28	2106.988	2105.9807	2106.013	0	137	2.00E-14	K.ELHSWISHSDLSPQDISR.G
29	2148.9773	2147.97	2148.0078	0	95	2.90E-10	K.HADSWWLHWQQWLAER.S
32	2254.9692	2253.9619	2253.9882	0	27	0.0021	K.DLVNNGGMPSQVDMDAFEVGK.N + 2 Oxidation (M)
34	2618.2908	2617.2835	2617.3421	0	134	4.00E-14	R.GQFVINLLTEAMSPTNSLSNPAAVK.R + Oxidation (M)
35	2618.293	2617.2857	2617.3421	0	136	2.30E-14	R.GQFVINLLTEAMSPTNSLSNPAAVK.R + Oxidation (M)
36	2945.4719	2944.4646	2944.5155	1	26	0.0028	R.HVAHFSLELKNVLLGQSELRPGDDDR.R

Ag-PhaC1$_{Pa}$

Peptide no.	M_r Observed	Exptl	Calculated	Miss[a)]	Score[b)]	Expected[c)]	Peptide[d)]
1	808.4259	807.4186	807.4351	0	48	1.50E-05	R.QPLHSAR.H
2	808.4373	807.43	807.4351	0	40	9.70E-05	R.QPLHSAR.H
6	902.4549	901.4476	901.4657	0	35	0.00034	K.AWLEQAGK.H
7	945.5485	944.5412	944.5477	0	16	2.70E-02	R.MVLLQAVR.Q + Oxidation (M)
8	960.5267	959.5194	959.54	1	33	5.20E-04	R.GKDLLTSAR.M
9	1018.5068	1017.4995	1017.5091	0	36	2.30E-04	R.LTATEDAAAR.A
10	1102.5591	1101.5518	1101.5527	1	62	5.60E-07	R.RVENATAEGR.A
11	1123.6243	1122.617	1122.6397	0	65	3.30E-07	K.SLLDGLGHLAK.D
12	1123.6356	1122.6283	1122.6397	0	54	4.00E-06	K.SLLDGLGHLAK.D
13	1180.6248	1179.6175	1179.64	0	64	4.10E-07	R.HVAHFSLELK.N
14	1230.5981	1229.5908	1229.5968	0	47	2.10E-05	K.FYVFDLSPDK.S
16	1277.6775	1276.6702	1276.6776	0	52	6.70E-06	K.NLATTEGAVVFR.N
17	1306.6832	1305.6759	1305.683	0	82	6.90E-09	R.NGVQTFIVSWR.N
18	1316.6763	1315.669	1315.6844	1	30	9.30E-04	R.IATGKQNATAEGR.A
20	1389.6586	1388.6513	1388.6646	0	44	4.20E-05	R.FMTNPELPAEPK.A + Oxidation (M)
21	1552.7253	1551.718	1551.7358	0	50	1.10E-05	R.FSDPAWSQNPLYK.R
22	1552.7335	1551.7262	1551.7358	0	29	0.0013	R.FSDPAWSQNPLYK.R
23	1570.8461	1569.8388	1569.8555	0	121	8.00E-13	R.LPAALHGEFVELFK.S
25	1657.8307	1656.8234	1656.8512	1	24	3.60E-03	K.FYVFDLSPDKSLAR.F
26	1657.8418	1656.8345	1656.8512	1	32	6.80E-04	K.FYVFDLSPDKSLAR.F
27	1722.9312	1721.9239	1721.9424	0	66	7.80E-07	K.QAAENTLNLNPVIGIR.G
28	1806.8674	1805.8601	1805.8907	2	48	1.50E-05	R.ADEAYRKADEALGAAQK.A
30	1939.9419	1938.9346	1938.9871	1	46	2.40E-05	K.NVLLGQSELRPGDDDRR.F
31	1939.965	1938.9577	1938.9871	1	35	2.90E-04	K.NVLLGQSELRPGDDDRR.F
32	1958.9553	1957.948	1957.9817	1	32	0.0027	R.RPGEEVNLTTTTVDDRR.I
33	2078.0969	2077.0896	2077.1167	0	96	2.40E-10	K.SNPLNRPGALEVSGTPIDLK.Q
34	2106.9761	2105.9688	2106.013	0	110	1.00E-11	K.ELHSWISHSDLSPQDISR.G
35	2106.9861	2105.9788	2106.013	0	50	1.00E-05	K.ELHSWISHSDLSPQDISR.G

PhaC1$_{Pa}$-Ag

Peptide no.	M_r Observed	Exptl	Calculated	Miss[a)]	Score[b)]	Expected[c)]	Peptide[d)]
1	945.5907	944.5834	944.5477	0	19	0.012	R.MVLLQAVR.Q + Oxidation (M)
3	1018.5368	1017.5295	1017.5091	0	76	2.30E-08	R.LTATEDAAAR.A
4	1018.5511	1017.5438	1017.5091	0	70	9.40E-08	R.LTATEDAAAR.A
6	1102.5912	1101.5839	1101.5527	1	76	2.60E-08	R.RVENATAEGR.A
8	1128.7003	1127.693	1127.6662	1	39	0.00013	R.GSVLAVAIDKR.G
9	1180.6682	1179.6609	1179.64	0	71	7.90E-08	R.HVAHFSLELK.N
10	1232.594	1231.5867	1231.5429	0	62	7.00E-07	K.AQQTADEANER.A
11	1247.6101	1246.6028	1246.5805	0	21	8.50E-03	R.YMQTYLAWR.K + Oxidation (M)
13	1277.7007	1276.6934	1276.6776	0	88	1.50E-09	K.NLATTEGAVVFR.N
14	1306.7054	1305.6981	1305.683	0	79	1.30E-08	R.NGVQTFIVSWR.N
17	1389.688	1388.6807	1388.6646	0	66	2.80E-07	R.FMTNPELPAEPK.A + Oxidation (M)
20	1552.7488	1551.7415	1551.7358	0	94	4.40E-10	R.FSDPAWSQNPLYK.R
21	1570.871	1569.8637	1569.8555	0	110	9.20E-12	R.LPAALHGEFVELFK.S
22	1657.8372	1656.8299	1656.8512	1	42	6.20E-05	K.FYVFDLSPDKSLAR.F
23	1675.8689	1674.8616	1674.8649	2	33	0.0005	R.RVENLTTTTVDDRR.I
26	1718.818	1717.8107	1717.806	0	88	1.60E-09	K.TYPAGEAAPGTYVHER.G
27	1783.8763	1782.869	1782.886	0	35	3.30E-04	K.NVLLGQSELRPGDDDR.R
28	1939.9861	1938.9788	1938.9871	1	60	1.10E-06	K.NVLLGQSELRPGDDDRR.F
29	2078.1182	2077.1109	2077.1167	0	136	2.30E-14	K.SNPLNRPGALEVSGTPIDLK.Q
31	2107.0269	2106.0196	2106.013	0	139	1.30E-14	K.ELHSWISHSDLSPQDISR.G
32	2148.9878	2147.9805	2148.0078	0	39	1.20E-04	K.HADSWWLHWQQWLAER.S
33	2254.9812	2253.9739	2253.9882	0	60	1.10E-06	K.DLVNNGGMPSQVDMDAFEVGK.N + 2 Oxidation (M)
34	2618.3159	2617.3086	2617.3421	0	96	2.70E-10	R.GQFVINLLTEAMSPTNSLSNPAAVK.R + Oxidation (M)
35	2618.3567	2617.3494	2617.3421	0	170	9.10E-18	R.GQFVINLLTEAMSPTNSLSNPAAVK.R + Oxidation (M)
36	3509.9448	3508.9375	3508.9406	0	74	4.00E-08	R.NDVLELIQYRPITESVHERPLLVVPPQINK.F

a) The number of missed cleavage sites.

b) The score is the -log10(P) value, where P is the probability that the observed match is a random event. Individual ion scores of >56 indicate identity or extensive homology (P ≤ 0.05).

c) Expected score based on BLAST search.

d) The sequence between the peptides was identified by MS. The amino acid before the period at the N terminal and that after the period at the C terminal indicate the cleavage sites.

Supplementary Table 2. Protein identification of dominant HCPs by peptide finger printing using MALDI-TOF MS

Peptide no.	M_r						
	Observed	Exptl	Calculated	Miss[a)]	Score[b)]	Expected[c)]	Peptide[d)]
**Band I**							
1	808.4307	807.4235	807.4715	0	55	0.24	KPLHSAR
2	828.4216	827.4143	827.4137	0	35	24	QNAAQAPK + Deamidated (NQ)
3	853.4464	852.4391	852.4276	0	3.20E+01	63	EMALHPR
4	860.3954	859.3881	859.3824	0	2.90E+01	1.70E+02	NFSSYSR
5	906.4799	905.4726	905.4429	0	3.10E+01	1.70E+02	CGFPDVIR
6	945.5456	944.5383	944.5113	1	41	20	NMPLLKGR + Deamidated (NQ); Oxidation (M)
7	1018.5084	1017.5011	1017.509	0	6.30E+01	0.13	LTATENAAAR + Deamidated (NQ)
8	1078.5586	1077.5513	1077.5607	0	47	5.2	EVVQGFFPR
9	1089.5977	1088.5904	1088.5462	1	33	1.30E+02	LRQTPTTSDA
10	1102.5491	1101.5418	1101.5778	0	5.80E+01	0.35	AGVSSPAGTTVR
11	1123.6383	1122.631	1122.6397	0	58	2.10E-01	K.SLLDGLGHLAK.D
12	1180.6326	1179.6253	1179.64	0	65	0.054	R.HVAHFSLELK.N
13	1230.5875	1229.5802	1229.509	0	54	0.84	MNMMDLSALR + Deamidated (NQ); 3 Oxidation (M)
14	1247.5771	1246.5698	1246.6517	1	38	33	ANSSALSAIEKR + Deamidated (NQ)
15	1262.6642	1261.6569	1261.6666	0	73	0.013	R.NDVLELIQYR.A
16	1277.6748	1276.6675	1276.6776	0	98	4.20E-05	K.NLATTEGAVVFR.N
17	1289.6681	1288.6608	1288.7139	0	3.50E+01	87	NLALTEGAVVFR
18	1306.6881	1305.6808	1305.683	0	68	0.04	R.NGVQTFIVSWR.N
19	1318.6893	1317.682	1317.6347	0	2.60E+01	6.40E+02	AAITAMPSQASDR
20	1338.6746	1337.6673	1337.6762	1	3.20E+01	1.50E+02	VAACDLAGKSTFR
21	1409.6619	1408.6546	1408.6623	1	60	0.21	WSELEEAFDKR
22	1421.6638	1420.6565	1420.6946	1	4.50E+01	6.4	ERELEELGYQR
23	1552.7418	1551.7345	1551.7358	0	77	0.0046	R.FSDPAWSQNPLYK.R
24	1620.7782	1619.7709	1619.7791	0	91	0.00024	R.LSDNPDYTAIINER.Q
25	1687.8799	1686.8726	1686.8835	1	37	54	NGRMGVAELASSLGVAR
26	1705.915	1704.9077	1704.9887	1	34	1.00E+02	DVPANTLVAGVPAVVKR
27	1722.9327	1721.9254	1721.9424	0	125	7.30E-08	K.QAAENTLNLNPVIGIR.G
28	1792.9099	1791.9026	1791.9057	0	2.90E+01	3.50E+02	GGFAYGPHQVLLSYQR
29	1792.9309	1791.9236	1791.9057	0	47	6.1	GGFAYGPHQVLLSYQR
30	1877.9371	1876.9298	1876.9319	0	109	4.30E-06	R.VGDVFPETPVIQYGNSR.L
31	1939.9679	1938.9606	1938.9871	1	75	9.20E-03	K.NVLLGQSELRPGDDDRR.F
32	2106.9988	2105.9915	2106.013	0	144	1.30E-09	K.ELHSWISHSDLSPQDISR.G
33	2107.0269	2106.0196	2106.013	0	-143	1.40E-09	K.ELHSWISHSDLSPQDISR.G
34	2254.9941	2253.9868	2253.9882	0	123	1.10E-07	K.DLVNNGGMPSQVDMDAFEVGK.N + 2 Oxidation (M)
35	2618.3281	2617.3208	2617.3421	0	116	7.40E-07	R.GQFVINLLTEAMSPTNSLSNPAAVK.R + Oxidation (M)
36	3509.9463	3508.939	3508.9406	0	72	1.20E-02	R.NDVLELIQYRPITESVHERPLLVVPPQINK.F
**Band II**							
1	828.4369	827.4296	827.4137	0	43	4.1	NEIQAPR + Deamidated (NQ)
2	888.4688	887.4615	887.4461	0	29	2.90E+02	NANINSVR + Deamidated (NQ)
3	929.4977	928.4905	928.5342	0	51	1.5	AELVGTLAR
4	945.5476	944.5403	944.4749	0	38	34	LPTNGLACR + Deamidated (NQ)
5	960.5375	959.5302	959.5036	0	52	1.3	AQVGVATSAR + Deamidated (NQ)
6	975.5542	974.5469	974.5549	0	54	0.8	FEQLVALR
7	986.5489	985.5416	985.4968	0	33	1.10E+02	EPSLAELDL
8	993.5276	992.5203	992.508	0	41	16	DFFKPSPR
9	1079.5712	1078.5639	1078.5771	0	47	4.6	SAYVSGQVIR
10	1123.6278	1122.6205	1122.6397	0	52	0.97	SLLDGLGHLAK
11	1142.6404	1141.6331	1141.6455	0	50	1.9	GIGAAIAQTLAR + Deamidated (NQ)
12	1142.6411	1141.6338	1141.6455	0	74	0.008	R.GIGAAIAETLAR.D
13	1149.6171	1148.6098	1148.619	0	57	0.42	K.AVLFDASGLTR.F
14	1158.5688	1157.5615	1157.5676	0	49	2.5	GAEDQLEGALR
15	1180.6403	1179.633	1179.64	0	55	0.62	HVAHFSLELK
16	1194.6215	1193.6142	1193.6193	0	46	4.8	YIAFANSPVGR
17	1230.5911	1229.5838	1229.5968	0	47	4.6	FYVFDLSPDK
18	1247.5825	1246.5752	1246.5805	0	33	1.00E+02	YMQTYLAWR + Oxidation (M)
19	1262.6631	1261.6558	1261.6666	0	56	0.73	NDVLELIQYR
20	1277.6764	1276.6691	1276.6776	0	95	7.80E-05	K.NLATTEGAVVFR.N
21	1289.6682	1288.6609	1288.7139	0	41	23	NLALTEGAVVFR
22	1306.686	1305.6787	1305.683	0	103	1.40E-05	R.NGVQTFIVSWR.N
23	1318.6794	1317.6721	1317.616	0	28	4.00E+02	AAIAANQTQADSR + 2 Deamidated (NQ)
24	1450.782	1449.7747	1449.7827	0	78	0.0038	R.DGAEVVLLDVPPAR.E
25	1559.8	1558.7927	1558.8719	0	33	1.60E+02	LLNSLFATSEVPIR
26	1705.9141	1704.9068	1704.8869	1	32	1.70E+02	AEKATQVPLMGEIYR
27	1705.9148	1704.9075	1705.0111	2	37	57	RTAIDKRPVAGPVAVR
28	1722.9375	1721.9302	1721.9424	0	128	3.30E-08	K.QAAENTLNLNPVIGIR.G
29	1783.8733	1782.866	1782.886	0	43	13	NVLLGQSELRPGDDDR
30	1848.9384	1847.9311	1847.9418	0	150	3.20E-10	K.LTDAVFAAVDGQFELPR.W
31	1939.973	1938.9657	1938.9871	1	56	0.67	K.NVLLGQSELRPGDDDRR.F
32	2247.2532	2246.2459	2246.2747	1	48	3.2	VVVLGRPPESLKDPVTASVQR
33	2490.3936	2489.3863	2489.4006	0	102	1.40E-05	R.VRPVDGPLVIGGSGALAEAVLPFAGK.L
34	2618.3447	2617.3374	2617.3421	0	118	5.00E-07	R.GQFVINLLTEAMSPTNSLSNPAAVK.R + Oxidation (M)
35	3509.926	3508.9187	3508.9406	0	78	0.0036	R.NDVLELIQYRPITESVHERPLLVVPPQINK.F
36	3509.9485	3508.9412	3508.9406	0	-66	0.055	R.NDVLELIQYRPITESVHERPLLVVPPQINK.F

[a)] The number of missed cleavage sites.

[b)] The score is the -log10(P) value, where P is the probability that the observed match is a random event. Individual ion scores of >56 indicate identity or extensive homology (P ≤ 0.05).

[c)] Expected score based on BLAST search.

[d)] The sequence between the peptides was identified by MS. The amino acid before the period at the N terminal and that after the period at the C terminal indicate the cleavage sites.

Supplementary Table 2. (*Continued*)

Band III

Peptide no.	M_r			Miss[a)]	Score[b)]	Expected[c)]	Peptide[d)]
	Observed	Exptl	Calculated				
1	808.4261	807.4188	807.4351	0	50	0.81	R.QPLHSAR.H
2	887.4237	886.4164	886.4185	0	33	62	YFTNSVR + Deamidated (NQ)
3	945.5349	944.5276	944.5841	1	45	8.30E+00	MVLLKAVR + Oxidation (M)
4	960.5441	959.5369	959.5036	0	54	0.95	AGGDLTITGR
5	1024.5002	1023.4929	1023.4985	0	44	6.7	TLQSEFSGR
6	1089.5287	1088.5214	1088.576	1	35	67	RPGMKTNLR + Deamidated (NQ); Oxidation (M)
7	1102.5559	1101.5486	1101.603	0	50	2.10E+00	VTLAGQSLEGK
8	1102.5585	1101.5512	1101.5526	1	49	3.2	AERAATAAEGR
9	1123.641	1122.6337	1122.6397	0	65	4.00E-02	K.SLLDGLGHLAK.D
10	1180.6158	1179.6085	1179.64	0	66	0.053	R.HVAHFSLELK.N
11	1192.6191	1191.6118	1191.6573	0	44	8.90E+00	VPMLFLSELK + Oxidation (M)
12	1230.588	1229.5807	1229.5968	0	50	2.20E+00	FYVFDLSPDK
13	1247.5695	1246.5622	1246.5805	0	39	21	YMQTYLAWR + Oxidation (M)
14	1262.6544	1261.6471	1261.6666	0	69	0.035	R.NDVLELIQYR.A
15	1277.6613	1276.654	1276.6776	0	98	4.30E-05	K.NLATTEGAVVFR.N
16	1289.6799	1288.6726	1288.7139	0	42	1.50E+01	NLALTEGAVVFR
17	1306.6748	1305.6675	1305.683	0	99	3.30E-05	R.NGVQTFIVSWR.N
18	1329.5942	1328.5869	1328.6077	0	51	1.1	K.YYFTENFFAK.A
19	1338.6759	1337.6686	1337.7667	0	30	2.10E+02	TTQLPAVVGSPIR
20	1406.6688	1405.6615	1405.6838	0	101	2.10E-05	R.DVLVNEYGVEGGR.V
21	1552.7375	1551.7302	1551.7358	0	67	0.053	R.FSDPAWSQNPLYK.R
22	1562.781	1561.7737	1561.7525	0	34	1.10E+02	LVQGFDEAVWNGAR + Deamidated (NQ)
23	1562.788	1561.7807	1561.7848	1	35	8.70E+01	QSQLQRLQAFQGR + 3 Deamidated (NQ)
24	1705.924	1704.9167	1704.9649	2	38	3.60E+01	GVGFRHGGLGRGPGLLR
25	1722.9219	1721.9146	1721.9424	0	127	5.30E-08	K.QAAENTLNLNPVIGIR.G
26	1783.8738	1782.8665	1782.886	0	53	1.3	K.NVLLGQSELRPGDDDR.R
27	1921.9922	1920.9849	1921.0268	2	32	1.80E+02	VQPSLEPNSKKAPKPGSR + 2 Deamidated (NQ)
28	1939.959	1938.9517	1938.9871	1	63	0.15	K.NVLLGQSELRPGDDDRR.F
29	2107.0146	2106.0073	2106.013	0	130	2.90E-08	K.ELHSWISHSDLSPQDISR.G
30	2132.9958	2131.9885	2132.0246	0	115	9.20E-07	R.VNAVGYGESRPVADNATAEGR.A
31	2278.2817	2277.2744	2277.1531	1	23	1.00E+03	MVKALGADCVGMSTVPEVIVAR + 2 Oxidation (M)
32	2311.2021	2310.1948	2310.2114	1	37	55	AGIVTQLQARCSVIAAANPVGGR + Carbamidomethyl (C); 2 Deamidated (NQ)
33	2431.1848	2430.1775	2430.3006	2	24	1.20E+03	VEAVVASDDGKIVFVGKEEQALK
34	2586.1782	2585.1709	2585.1518	0	162	1.70E-11	K.QYPSTSTTVEGHTDSVGTDAYNQK.L
35	2618.3262	2617.3189	2617.3421	0	155	9.70E-11	R.GQFVINLLTEAMSPTNSLSNPAAVK.R + Oxidation (M)
36	3509.9482	3508.9409	3508.9406	0	98	2.90E-05	R.NDVLELIQYRPITESVHERPLLVVPPQINK.F

Band IV

Peptide no.	Observed	Exptl	Calculated	Miss	Score	Expected	Peptide
1	828.3899	827.3826	827.429	1	42	3	AGKYAYR
2	849.4656	848.4584	848.4062	0	32	1.10E+02	NQACTIAK + Deamidated (NQ)
3	907.4406	906.4333	906.4229	0	22	1.10E+03	SGAGAEMLR + Oxidation (M)
4	914.4651	913.4578	913.5233	0	28	2.00E+02	VGLLENLR + Deamidated (NQ)
5	934.4504	933.4431	933.4767	1	34	8.20E+01	SSNELKAGK + Deamidated (NQ)
6	973.4255	972.4182	972.4512	0	21	5.60E+02	LDNAPQASR + 2 Deamidated (NQ)
7	973.4382	972.431	972.4876	0	29	1.20E+02	NAAPSVSEAK
8	1064.5614	1063.5541	1063.5298	0	39	2.80E+01	LSNFGEQLR + Deamidated (NQ)
9	1180.6057	1179.5984	1179.64	0	42	13	HVAHFSLELK
10	1211.605	1210.5977	1210.5805	0	32	1.10E+02	LVAMSFGWQR + Deamidated (NQ); Oxidation (M)
11	1223.6154	1222.6081	1222.6743	1	34	9.50E+01	KMEYLLSALR
12	1223.6355	1222.6282	1222.5829	1	31	1.80E+02	KEYDQLSPSR + Deamidated (NQ)
13	1232.5498	1231.5425	1231.623	1	31	1.20E+02	RGVVMNLSNPK + 2 Deamidated (NQ); Oxidation (M)
14	1232.5765	1231.5692	1231.6482	1	30	2.20E+02	KGILMNLSNPK + 2 Deamidated (NQ); Oxidation (M)
15	1247.5802	1246.5729	1246.634	1	27	4.00E+02	VVEMNLDAGKR + Oxidation (M)
16	1263.6379	1262.6306	1262.6506	0	32	1.60E+02	NIELAINSFNK + Deamidated (NQ)
17	1277.6837	1276.6764	1276.7	0	33	1.30E+02	IDIHRPAQTAR
18	1306.6764	1305.6691	1305.683	0	53	1.3	NGVQTFIVSWR
19	1306.6862	1305.6789	1305.683	0	47	5.20E+00	NGVQTFIVSWR
20	1329.61	1328.6027	1328.6758	0	34	68	ALAAMETSVPAPR + Oxidation (M)
21	1389.6544	1388.6471	1388.6646	0	64	0.086	R.FMTNPELPAEPK.A + Oxidation (M)
22	1405.6636	1404.6563	1404.7473	0	24	1.00E+03	LLAQNINPTHQR + Deamidated (NQ)
23	1440.7407	1439.7334	1439.7521	0	60	2.20E-01	FHIDQVLALNDR
24	1464.6908	1463.6835	1463.6674	1	38	3.70E+01	KTAGQMNSATANPR + 2 Deamidated (NQ); Oxidation (M)
25	1528.7229	1527.7156	1527.7239	1	64	0.095	R.SYQSGVLEGKDMAK.V + Oxidation (M)
26	1528.7593	1527.752	1527.798	1	30	2.50E+02	RHAEGALEFMLVR
27	1570.8456	1569.8383	1569.8555	0	123	1.10E-07	R.LPAALHGEFVELFK.S
28	1586.829	1585.8217	1585.8174	0	41	2.00E+01	LPAALHGELVEMYK + Oxidation (M)
29	1586.8409	1585.8336	1585.8174	0	43	1.30E+01	LPAALHGELVEMYK + Oxidation (M)
30	1683.8477	1682.8404	1682.7426	1	23	1.30E+03	TVMNCDNICVVKDGR + Deamidated (NQ); Oxidation (M)
31	1718.7946	1717.7873	1717.806	0	129	3.40E-08	K.TYPAGEAAPGTYVHER.-
32	2078.0981	2077.0908	2077.1167	0	126	7.70E-08	K.SNPLNRPGALEVSGTPIDLK.Q
33	2090.0444	2089.0371	2088.982	0	23	1.50E+03	SGHGMSGVEIIANAMQTLQK + 2 Deamidated(NQ);Oxidation (M)
34	2148.9944	2147.9871	2148.0078	0	103	1.50E-05	K.HADSWWLHWQQWLAER.S
35	2180.9885	2179.9812	2179.9864	0	24	1.20E+03	HADSWWLHWQQWITER + 2 Deamidated (NQ)
36	2422.1897	2421.1824	2421.2111	0	100	3.30E-05	K.CEFILSNSGHIQSILNPPGNPK.A + Carbamidomethyl (C)

a) The number of missed cleavage sites.

b) The score is the -log10(*P*) value, where P is the probability that the observed match is a random event. Individual ion scores of >56 indicate identity or extensive homology (P ≤ 0.05).

c) Expected score based on BLAST search.

d) The sequence between the peptides was identified by MS. The amino acid before the period at the N terminal and that after the period at the C terminal indicate the cleavage sites.

Supplementary Table 2. (*Continued*)

Band V

Peptide no.	M_r						
	Observed	Exptl	Calculated	Miss[a)]	Score[b)]	Expected[c)]	Peptide[d)]
1	803.4761	802.4688	802.4337	0	53	6.40E-01	SIAYPPR
2	817.3987	816.3915	816.3879	0	53	0.76	R.HSVGFDR.F
3	828.3979	827.3906	827.429	1	27	9.80E+01	KGAYAYR
4	828.4042	827.3969	827.3926	0	28	8.00E+01	GAQYAYR
5	891.3619	890.3546	890.3518	0	43	2.70E+00	HGDNEYR + Deamidated (NQ)
6	903.3601	902.3528	902.377	0	30	2.90E+01	YANDTYR + Deamidated (NQ)
7	924.4958	923.4885	923.4964	0	40	15	SSYVIEVK
8	939.4716	938.4643	938.428	0	29	1.80E+02	GLDGAMFGR + Oxidation (M)
9	951.488	950.4808	950.4743	0	33	9.40E+01	AITCTSNLK + Deamidated (NQ)
10	951.5015	950.4942	950.492	0	42	1.20E+01	SSTVSTELK
11	973.5695	972.5622	972.5577	2	43	1.10E+01	RITSGQRR
12	973.5701	972.5629	972.5716	1	37	4.50E+01	GVLTVSGGKR
13	983.5108	982.5035	982.4641	0	25	4.60E+02	INSTTTMSK + Deamidated (NQ)
14	1010.5897	1009.5824	1009.6535	1	36	2.20E+01	IIEAKVLPK
15	1119.5581	1118.5508	1118.5455	0	26	6.50E+02	INSINQSDVK + 2 Deamidated (NQ)
16	1163.6146	1162.6073	1162.6499	0	32	1.40E+02	GVFGSLAPLFR
17	1193.6244	1192.6171	1192.6088	0	44	9.90E+00	AATNLFESALR + Deamidated (NQ)
18	1211.624	1210.6167	1210.5982	0	92	1.10E-04	R.FNDLFESALR.N
19	1222.6582	1221.6509	1221.6506	0	75	6.50E-03	M.SNAFSLAPLFR.H
20	1234.6425	1233.6352	1233.6466	0	41	21	VNAVFTAQATGR
21	1306.6952	1305.6879	1305.683	0	60	2.50E-01	NGVQTFIVSWR
22	1334.7163	1333.709	1333.7506	1	34	9.40E+01	KIWLAGLGAYSR
23	1409.7491	1408.7418	1408.7422	0	60	2.10E-01	R.IAINGQRPALDNQ.-
24	1421.6964	1420.6891	1420.6834	0	39	34	LDVATGEAIDFDR
25	1421.6973	1420.69	1420.6834	0	41	2.10E+01	LNVATGEAIDFDR + Deamidated (NQ)
26	1539.8849	1538.8776	1538.878	0	151	1.20E-10	K.AASLANGLLNIDLVR.L
27	1551.8749	1550.8676	1550.9396	0	46	4.90E+00	VAGGIAAISGVGILAGLI
28	1568.7222	1567.7149	1567.7154	0	40	2.00E+01	NEAGSTYPPYNVEK
29	1580.7244	1579.7171	1579.7816	1	25	6.40E+02	GGGGTGGGGSTGPVPPGRR
30	1580.7321	1579.7248	1579.7729	0	26	5.90E+02	EELEAYQSAGLLTR + Deamidated (NQ)
31	1683.8401	1682.8328	1682.8992	0	29	3.50E+02	VVQTGNVGLFHVVGAGK + 2 Deamidated (NQ)
32	1702.8475	1701.8402	1701.8434	0	120	2.60E-07	K.STDNVTYLHQGIAQR.A
33	1718.8398	1717.8325	1717.837	2	38	4.50E+01	SDKDAEQVIAEKQEK + Deamidated (NQ)
34	2009.9786	2008.9713	2008.9755	1	95	1.00E-04	R.HSVGFDRFNDLFESALR.N
35	2116.0906	2115.0833	2115.0848	0	182	1.90E-13	R.IVIAAAGFQEEDLDLQVER.G
36	2132.0823	2131.075	2131.0909	1	35	96	GDVIALGFNQELDDLRSIR + Deamidated (NQ)

Band VI

1	852.4163	851.409	851.3773	0	39	1.70E+01	EGEAYGAR
2	908.4755	907.4682	907.4433	0	39	2.30E+01	EASTMTLR
3	918.4775	917.4703	917.4276	0	22	1.50E+03	MNEPSLAR + Deamidated (NQ)
4	950.4763	949.469	949.4691	0	24	8.50E+02	LDFVTCPR
5	973.4709	972.4636	972.4876	0	44	7.70E+00	ADEALGAAQK
6	979.588	978.5807	978.5862	0	48	1.30E+00	LHIINLEK
7	1018.5156	1017.5083	1017.5091	0	80	2.40E-03	R.LTATEDAAAR.A
7	1018.5156	1017.5083	1017.509	0	82	1.50E-03	LTATENAAAR + Deamidated (NQ)
8	1030.5343	1029.527	1029.5567	1	53	1.2	RASTPSAAAAK
9	1078.5746	1077.5673	1077.5818	0	42	13	GSFLSINAIR + Deamidated (NQ)
10	1101.589	1100.5817	1100.5826	1	69	0.033	R.KADEALGAAQK.A
11	1109.5554	1108.5481	1108.5526	0	69	2.50E-02	K.VGAHFGHQTR.Y
12	1119.5812	1118.5739	1118.5203	0	42	1.70E+01	ANAQTAVSEAR + 2 Deamidated (NQ)
13	1149.6067	1148.5994	1148.6189	0	32	1.60E+02	IPPNDPNLLR + Deamidated (NQ)
14	1166.5988	1165.5915	1165.5914	0	31	1.50E+02	SLACGGNIYIR
15	1221.7104	1220.7031	1220.6401	0	42	8.30E+00	ELLFSGGELTR
16	1232.5547	1231.5474	1231.5429	0	91	1.40E-04	K.AQQTADEANER.A
17	1244.569	1243.5617	1243.568	0	34	5.80E+01	EPLTDAENAQR + Deamidated (NQ)
18	1306.6948	1305.6875	1305.683	0	75	8.20E-03	R.NGVQTFIVSWR.N
19	1323.6692	1322.6619	1322.65	1	25	7.10E+02	MSETKTEAAAIR + Oxidation (M)
20	1338.6755	1337.6682	1337.6874	2	31	2.00E+02	SRGNKGGFMVIR + Deamidated (NQ); Oxidation (M)
21	1409.7516	1408.7443	1408.7674	1	28	3.50E+02	LAQRVVLSNGHLT + 2 Deamidated (NQ)
22	1418.8083	1417.801	1417.7426	1	45	5.90E+00	RPVRDDASLSFR
23	1442.7574	1441.7501	1441.7024	0	26	5.50E+02	IPAAQFDGMHVQK + Deamidated (NQ)
24	1505.7699	1504.7626	1504.7674	0	54	1.20E+00	YLALLPYTDSHGR
25	1539.8627	1538.8554	1538.878	0	-70	1.80E-02	K.AASLANGLLNIDLVR.L
26	1539.8654	1538.8581	1538.878	0	71	1.50E-02	K.AASLANGLLNIDLVR.L
27	1586.8965	1585.8892	1585.9192	1	42	1.30E+01	ERVPVSIYLVNGIK
28	1586.9182	1585.9109	1585.9192	1	36	4.60E+01	ERVPVSIYLVNGIK
29	1619.8271	1618.8198	1618.7621	0	36	7.10E+01	LSGGGGGGLTVCLEDER
30	1667.8622	1666.8549	1666.8389	0	40	2.90E+01	TLPMFNEALTFVER
31	1683.8398	1682.8325	1682.8338	0	83	1.20E-03	K.TLPMFNEALTFVER.L + Oxidation (M)
32	1699.8374	1698.8301	1698.8974	0	26	6.40E+02	VITGGIGIIPGATMNER + Deamidated (NQ)
33	1806.891	1805.8837	1805.8907	2	119	4.10E-07	R.ADEAYRKADEALGAAQK.A
34	2024.9935	2023.9862	2023.9792	0	108	4.70E-06	K.GYGFITPESGPDVFVHFR.A
35	2078.1113	2077.104	2077.1167	0	78	0.0039	K.SNPLNRPGALEVSGTPIDLK.Q
36	2206.2007	2205.1934	2205.0293	0	21	2.00E+03	MPIMTETAVAAEEASLPQAGR + Deamidated(NQ); 2 Oxidation (M)

[a)] The number of missed cleavage sites.

[b)] The score is the -log10(*P*) value, where P is the probability that the observed match is a random event. Individual ion scores of >56 indicate identity or extensive homology (P ≤ 0.05).

[c)] Expected score based on BLAST search.

[d)] The sequence between the peptides was identified by MS. The amino acid before the period at the N terminal and that after the period at the C terminal indicate the cleavage sites.

Supplementary Table 2. (*Continued*)

Band VII

Peptide no.	M_r Observed	Exptl	Calculated	Miss[a)]	Score[b)]	Expected[c)]	Peptide[d)]
1	808.4423	807.435	807.4715	0	50	8.20E-01	KPLHSAR
2	929.554	928.5467	928.5342	0	38	3.30E+01	LLEAGATVR
3	945.5618	944.5545	944.5841	1	47	4.00E+00	MVLLKAVR + Oxidation (M)
4	960.5502	959.5429	959.4924	0	42	1.30E+01	QGDIITANK + Deamidated (NQ)
5	1018.5213	1017.514	1017.509	0	62	1.50E-01	LTATENAAAR + Deamidated (NQ)
6	1102.5682	1101.5609	1101.5414	0	54	1.00E+00	NIENVTAQGR + Deamidated (NQ)
7	1128.6777	1127.6704	1127.6523	2	45	4.60E+00	RSSPVALSRR
8	1180.6552	1179.6479	1179.64	0	69	2.30E-02	R.HVAHFSLELK.N
9	1194.6394	1193.6321	1193.604	1	36	5.30E+01	GIGYLSKDDAR
10	1201.5947	1200.5874	1200.6536	1	37	4.40E+01	LDMKLNLGPGK + Oxidation (M)
11	1230.6115	1229.6042	1229.5968	0	56	5.30E-01	K.FYVFDLSPDK.S
12	1247.6042	1246.5969	1246.5805	0	36	6.40E+01	YMQTYLAWR + Oxidation (M)
13	1262.6844	1261.6771	1261.6666	0	55	8.10E-01	NDVLELIQYR
14	1277.6947	1276.6874	1276.6776	0	104	9.30E-06	K.NLATTEGAVVFR.N
15	1289.6853	1288.678	1288.7139	0	39	3.50E+01	NLALTEGAVVFR
16	1306.7017	1305.6944	1305.683	0	91	2.00E-04	R.NGVQTFIVSWR.N
17	1318.6924	1317.6851	1317.6131	0	33	1.20E+02	AGVMIMGMTTYK + Oxidation (M)
18	1318.6984	1317.6911	1317.6929	0	34	1.10E+02	ALEPTFADLSVR
19	1338.6796	1337.6723	1337.6034	0	37	4.60E+01	NGVGFGDLGEMSR
20	1389.6727	1388.6654	1388.6646	0	57	5.00E-01	R.FMTNPELPAEPK.A + Oxidation (M)
21	1403.6923	1402.685	1402.6576	0	27	4.90E+02	STPSVNQLQAAQR + 4 Deamidated (NQ)
22	1442.7759	1441.7686	1441.7314	0	32	1.50E+02	ALPAHLADSTQYR
23	1552.7452	1551.7379	1551.7358	0	72	1.60E-02	R.FSDPAWSQNPLYK.R
24	1570.8711	1569.8638	1569.8555	0	125	6.50E-08	R.LPAALHGEFVELFK.S
25	1675.8722	1674.8649	1674.7598	1	48	5.00E+00	NGVNPKFENDDDVGR
26	1687.8667	1686.8594	1686.8577	0	28	4.80E+02	GVDLDFLVGNSGAPVAR + Deamidated (NQ)
27	1705.9324	1704.9251	1704.9159	0	35	7.40E+01	VENLTNPLGIGTPQPR
28	1722.962	1721.9547	1721.9424	0	120	2.10E-07	K.QAAENTLNLNPVIGIR.G
29	2078.1313	2077.124	2077.1167	0	126	5.90E-08	K.SNPLNRPGALEVSGTPIDLK.Q
30	2107.0259	2106.0186	2106.013	0	132	2.00E-08	K.ELHSWISHSDLSPQDISR.G
31	2149.0164	2148.0091	2148.0078	0	90	0.00032	K.HADSWWLHWQQWLAER.S
32	2255.0002	2253.9929	2253.9882	0	116	5.80E-07	K.DLVNNGGMPSQVDMDAFEVGK.N + 2 Oxidation (M)
33	2311.1963	2310.189	2310.1492	1	36	8.30E+01	VIVQNAGRKPNDVVYTYSER + 3 Deamidated (NQ)
34	2618.3523	2617.345	2617.3421	0	167	7.00E-12	R.GQFVINLLTEAMSPTNSLSNPAAVK.R + Oxidation (M)
35	2774.4446	2773.4373	2773.4432	1	112	2.20E-06	R.GQFVINLLTEAMSPTNSLSNPAAVKR.F + Oxidation (M)
36	3509.9614	3508.9541	3508.9406	0	89	2.40E-04	R.NDVLELIQYRPITESVHERPLLVVPPQINK.F

Band VIII

Peptide no.	M_r Observed	Exptl	Calculated	Miss[a)]	Score[b)]	Expected[c)]	Peptide[d)]
1	808.4412	807.4339	807.4715	0	50	4.70E-01	KPLHSAR
2	828.4234	827.4161	827.4501	0	36	1.30E+01	DGALLPSR
3	929.564	928.5567	928.5892	1	50	1.30E+00	MVLLKAVR
4	945.5574	944.5502	944.5291	1	35	4.10E+01	LDSGQKAVK
5	960.5554	959.5481	959.54	1	65	4.00E-02	R.GKDLLTSAR.M
6	1123.6534	1122.6461	1122.6397	0	51	5.30E-01	SLLDGLGHLAK
7	1139.5364	1138.5291	1138.4682	0	20	5.70E+02	LCCCGNNISGR
8	1180.6503	1179.643	1179.64	0	65	2.60E-02	R.HVAHFSLELK.N
9	1215.5934	1214.5861	1214.5601	0	24	3.90E+02	DMSSVSSYALR
10	1230.6095	1229.6022	1229.5968	0	59	1.20E-01	K.FYVFDLSPDK.S
11	1247.6006	1246.5933	1246.6703	0	33	4.20E+01	SLGCALGTISVAR
12	1262.6823	1261.675	1261.6666	0	69	1.30E-02	R.NDVLELIQYR.A
13	1277.6927	1276.6854	1276.6776	0	101	8.40E-06	K.NLATTEGAVVFR.N
14	1289.6714	1288.6641	1288.7074	1	27	2.40E+02	AASPRFVMIAAR
15	1289.6874	1288.6801	1288.7139	0	38	1.80E+01	NLALTEGAVVFR
16	1306.7019	1305.6946	1305.683	0	99	1.40E-05	R.NGVQTFIVSWR.N
17	1322.691	1321.6837	1321.6415	1	29	1.20E+02	NGKVTFYNHDK
18	1338.6855	1337.6782	1337.7052	0	31	75	NSSTVTLHVPQR
19	1375.6859	1374.6786	1374.6739	0	33	4.90E+01	QIDLSEVSSNQR
20	1552.7577	1551.7504	1551.7358	0	104	3.00E-06	R.FSDPAWSQNPLYK.R
21	1687.8848	1686.8775	1686.9152	2	32	6.00E+01	KATVENVVAEKSDGIK
22	1705.932	1704.9247	1704.9159	0	43	4.70E+00	VENLTNPLGIGTPQPR
23	1722.955	1721.9477	1721.9424	0	125	2.30E-08	K.QAAENTLNLNPVIGIR.G
24	1755.9797	1754.9724	1754.9461	2	21	6.60E+02	ERLEEALVTVMRGPR
25	1778.9189	1777.9116	1778.0163	1	25	2.90E+02	QLGPALGGRLTEAVAAVR
26	1783.8943	1782.887	1782.886	0	68	1.40E-02	K.NVLLGQSELRPGDDDR.R
27	1939.9949	1938.9876	1938.9871	1	71	7.00E-03	K.NVLLGQSELRPGDDDRR.F
28	2107.0254	2106.0181	2106.013	0	-139	8.70E-10	K.ELHSWISHSDLSPQDISR.G
29	2107.0298	2106.0225	2106.013	0	141	5.20E-10	K.ELHSWISHSDLSPQDISR.G
30	2254.9885	2253.9812	2254.0852	0	21	3.10E+02	VLQVDASTLLESIPDEEDPNA
31	2255.0098	2254.0025	2254.0938	2	22	2.90E+02	QAAGKATDDASLHAEGTAQERK
32	2311.219	2310.2117	2310.2444	1	31	5.40E+01	NQRALLDAAAAVFVASGVDAPVR
33	2596.3848	2595.3775	2595.322	2	21	4.60E+02	IDNLFKKVASFTEPEIQSEWSK
34	2618.3601	2617.3528	2617.3244	0	82	4.10E-04	R.GQFVINLMTEAMAPTNTLSNPAAVK.R
35	2774.4446	2773.4373	2773.4255	1	29	7.70E+01	GQFVINLMTEAMAPTNTLSNPAAVKR
36	3509.9651	3508.9578	3508.9406	0	100	3.30E-06	R.NDVLELIQYRPITESVHERPLLVVPPQINK.F

a) The number of missed cleavage sites.

b) The score is the -log10(*P*) value, where P is the probability that the observed match is a random event. Individual ion scores of >56 indicate identity or extensive homology (P ≤ 0.05).

c) Expected score based on BLAST search.

d) The sequence between the peptides was identified by MS. The amino acid before the period at the N terminal and that after the period at the C terminal indicate the cleavage sites.

Supplementary Table 2. (*Continued*)

Band IX

Peptide no.	M_r Observed	Exptl	Calculated	Miss[a)]	Score[b)]	Expected[c)]	Peptide[d)]
1	808.4493	807.4421	807.4715	0	28	1.20E+02	KPLHSAR
2	929.5632	928.5559	928.509	1	33	9.60E+01	LDQGAKAAR
3	934.4607	933.4535	933.4953	1	35	7.30E+01	AAMLEKAGK + Oxidation (M)
4	945.5638	944.5565	944.5841	1	42	1.30E+01	MVLLKAVR + Oxidation (M)
5	960.5496	959.5423	959.5512	1	40	2.30E+01	RSGILTSAR
6	1067.5352	1066.5279	1066.5295	0	49	2.20E+00	SYQSGVLEGK
7	1067.5354	1066.5281	1066.5295	0	57	3.80E-01	R.SYQSGVLEGK.D
8	1180.6586	1179.6513	1179.64	0	61	1.40E-01	R.HVAHFSLELK.N
9	1197.5535	1196.5462	1196.5972	1	25	4.80E+02	VNQTSFKAMR + Oxidation (M)
10	1218.67	1217.6627	1217.6881	0	34	1.00E+02	GPVIGVHLVGDR
11	1230.6108	1229.6035	1229.5968	0	52	1.40E+00	FYVFDLSPDK
12	1247.5995	1246.5922	1246.5805	0	38	3.30E+01	YMQTYLAWR + Oxidation (M)
13	1262.6755	1261.6682	1261.7142	1	42	1.70E+01	ITNNQLLKYR
14	1277.694	1276.6867	1276.6776	0	69	3.10E-02	K.NLATTEGAVVFR.N
15	1289.6842	1288.6769	1288.7139	0	58	4.60E-01	K.NLALTEGAVVFR.N
16	1306.691	1305.6837	1305.683	0	-62	1.40E-01	R.NGVQTFIVSWR.N
17	1306.6958	1305.6885	1305.683	0	66	5.50E-02	R.NGVQTFIVSWR.N
18	1389.6802	1388.6729	1388.6646	0	74	1.00E-02	R.FMTNPELPAEPK.A + Oxidation (M)
19	1405.6807	1404.6734	1404.5762	0	27	5.60E+02	GEGAPQLCDACQR + Carbamidomethyl (C); Deamidated (NQ)
20	1528.7473	1527.74	1527.7239	1	68	3.90E-02	R.SYQSGVLEGKDMAK.V + Oxidation (M)
21	1552.7577	1551.7504	1551.7358	0	47	4.70E+00	FSDPAWSQNPLYK
22	1570.8755	1569.8682	1569.8555	0	120	2.00E-07	R.LPAALHGEFVELFK.S
22	1570.8755	1569.8682	1569.8225	0	69	2.80E-02	R.LPAALHGELVEMFK.S + Oxidation (M)
23	1705.9291	1704.9218	1704.9159	0	33	1.20E+02	QASEHTLGLNPVIGIR + Deamidated (NQ)
24	1722.9612	1721.9539	1721.9424	0	97	4.40E-05	K.QAAENTLNLNPVIGIR.G
25	1734.9114	1733.9041	1733.8882	2	28	4.40E+02	DGKPKKEALNNFMSR
26	1783.8937	1782.8864	1782.886	0	48	4.50E+00	NVLLGQSELRPGDDDR
27	1939.9922	1938.9849	1938.9871	1	55	8.40E-01	K.NVLLGQSELRPGDDDRR.F
28	2078.1379	2077.1306	2077.1167	0	132	1.60E-08	K.SNPLNRPGALEVSGTPIDLK.Q
29	2090.0972	2089.0899	2089.2371	2	21	2.20E+03	LPQREGPRIQTLSILVLR + Deamidated (NQ)
30	2107.019	2106.0117	2106.013	0	118	5.20E-07	K.ELHSWISHSDLSPQDISR.G
31	2181.0098	2180.0025	2180.0183	0	25	8.60E+02	LDGMLQHFGQHVLSGCFFK + Deamidated (NQ); Oxidation (M)
32	2181.0115	2180.0042	2180.0035	1	25	9.00E+02	RSWPDEAAWHEAAQGLAQR + 2 Deamidated (NQ)
33	2255.009	2254.0017	2253.9882	0	73	1.20E-02	K.DLVNNGGMPSQVDMDAFEVGK.N + 2 Oxidation (M)
33	2255.009	2254.0017	2253.9882	0	73	1.20E-02	K.DLVNNGGMPSQVNMDAFEVGK.N + Deamidated (NQ); 2 Oxidation (M)
34	2422.2246	2421.2173	2421.2111	0	120	3.00E-07	K.CEFILSNSGHIQSILNPPGNPK.A + Carbamidomethyl (C)
35	2618.3459	2617.3386	2617.3421	0	137	6.40E-09	R.GQFVINLLTEAMSPTNSLSNPAAVK.R + Oxidation (M)
36	3509.9541	3508.9468	3508.9406	0	108	3.40E-06	R.NDVLELIQYRPITESVHERPLLVVPPQINK.F

Band X

Peptide no.	M_r Observed	Exptl	Calculated	Miss[a)]	Score[b)]	Expected[c)]	Peptide[d)]
1	808.4367	807.4294	807.4715	0	50	8.00E-01	KPLHSAR
2	828.4236	827.4164	827.4501	0	36	1.60E+01	ATALPAQR + Deamidated (NQ)
3	846.4392	845.4319	845.4759	0	35	7.20E+01	QFVSLPR
4	846.4459	845.4386	845.4429	0	45	7	SGMIVSPR
5	929.5566	928.5493	928.5892	1	44	8.10E+00	MVLLKAVR
6	945.5467	944.5394	944.5113	0	48	3.50E+00	VCGQLQAVK
7	960.5452	959.5379	959.54	1	58	3.70E-01	R.GKDLLTSAR.M
8	972.5431	971.5358	971.5036	0	36	5.70E+01	NTPGSELVR
9	1018.509	1017.5017	1017.509	0	73	1.10E-02	R.LTATENAAAR.A + Deamidated (NQ)
10	1102.5579	1101.5506	1101.5414	0	55	7.10E-01	EAAAIAADSAGR
11	1123.6423	1122.635	1122.6397	0	47	3.00E+00	SLLDGLGHLAK
12	1123.6477	1122.6404	1122.6397	0	49	1.50E+00	SLLDGLGHLAK
13	1180.6394	1179.6321	1179.64	0	68	2.70E-02	R.HVAHFSLELK.N
14	1192.6409	1191.6336	1191.6387	0	34	8.40E+01	DINTFVIELK + Deamidated (NQ)
15	1232.5486	1231.5413	1231.5429	0	75	4.80E-03	K.AQQTADEANER.A
16	1247.5829	1246.5756	1246.5805	0	35	6.70E+01	YMQTYLAWR + Oxidation (M)
17	1263.5953	1262.588	1262.6401	1	36	5.80E+01	GRLQTQMGSLR + Deamidated (NQ); Oxidation (M)
18	1306.6842	1305.6769	1305.683	0	80	2.30E-03	R.NGVQTFIVSWR.N
19	1318.6875	1317.6802	1317.6347	0	30	2.60E+02	LDAQGGDGIAMVR + Oxidation (M)
20	1389.6829	1388.6756	1388.7048	1	22	1.80E+03	FRDVSQDVLGPR + Deamidated (NQ)
21	1403.6975	1402.6902	1402.7106	1	35	9.20E+01	RHGFPTELYQR
22	1442.7704	1441.7631	1441.7215	0	30	2.50E+02	FIGAAPPEGHGPHR
23	1519.7671	1518.7598	1518.7817	0	36	6.50E+01	INLNNVYSLEIPK + 3 Deamidated (NQ)
24	1552.7448	1551.7375	1551.7358	0	94	9.60E-05	R.FSDPAWSQNPLYK.R
25	1570.8499	1569.8426	1569.8555	0	92	1.50E-04	R.LPAALHGEFVELFK.S
26	1606.8281	1605.8208	1605.825	0	41	2.40E+01	VLYEIEGVSEEIAR
27	1675.8597	1674.8524	1674.8611	0	49	4.00E+00	AMQDITTALTVGAEVR
28	1705.9166	1704.9093	1704.9709	1	41	2.20E+01	MPAKNLAPVGGVPLVAR + Oxidation (M)
29	1705.9314	1704.9241	1704.9159	0	31	1.80E+02	QASEHTLGLNPVIGIR + Deamidated (NQ)
30	1722.9398	1721.9325	1721.9424	0	120	2.30E-07	K.QAAENTLNLNPVIGIR.G
31	1783.8936	1782.8863	1782.886	0	52	1.80E+00	NVLLGQSELRPGDDDR
32	1922.0448	1921.0375	1921.0493	2	35	7.60E+01	GVSSPVRQPSLLGPGSARR + Deamidated (NQ)
33	1939.9817	1938.9744	1938.9871	1	60	2.70E-01	K.NVLLGQSELRPGDDDRR.F
34	2078.1196	2077.1123	2077.1167	0	82	1.60E-03	K.SNPLNRPGALEVSGTPIDLK.Q
35	2107.0159	2106.0086	2106.013	0	124	1.30E-07	K.ELHSWISHSDLSPQDISR.G
36	2618.3279	2617.3206	2617.3421	0	135	1.00E-08	R.GQFVINLLTEAMSPTNSLSNPAAVK.R + Oxidation (M)

a) The number of missed cleavage sites.

b) The score is the -log10(*P*) value, where P is the probability that the observed match is a random event. Individual ion scores of >56 indicate identity or extensive homology (P ≤ 0.05).

c) Expected score based on BLAST search.

d) The sequence between the peptides was identified by MS. The amino acid before the period at the N terminal and that after the period at the C terminal indicate the cleavage sites.

Supplementary Table 2. (*Continued*)

Band XI

Peptide no.	M_r						
	Observed	Exptl	Calculated	Miss[a)]	Score[b)]	Expected[c)]	Peptide[d)]
1	808.4443	807.437	807.4715	0	32	4.90E+01	KPLHSAR
2	808.4446	807.4373	807.4715	0	38	1.20E+01	KPIHSAR
3	817.4036	816.3963	816.3878	0	34	5.90E+01	SHVGFNR + Deamidated (NQ)
4	828.4076	827.4004	827.4501	0	25	1.90E+02	ANPVSALR + Deamidated (NQ)
5	891.3716	890.3644	890.377	0	21	6.20E+02	NPPDEYR + Deamidated (NQ)
6	902.4819	901.4747	901.4505	0	38	4.20E+01	EGAQAELGK
7	934.47	933.4628	933.4953	1	32	1.60E+02	AAMLEKAGK + Oxidation (M)
8	939.4736	938.4663	938.428	0	38	2.40E+01	GLDGAMFGR + Oxidation (M)
9	945.5576	944.5503	944.5365	0	37	4.00E+01	VEAALAIMK
10	1018.526	1017.5187	1017.509	0	59	3.00E-01	R.LTATENAAAR.A + Deamidated (NQ)
11	1102.561	1101.5537	1101.5526	1	56	6.10E-01	AERAATAAEGR
12	1180.6501	1179.6428	1179.64	0	60	1.60E-01	R.HVAHFSLELK.N
13	1197.5682	1196.5609	1196.5197	0	25	5.10E+02	TGAANYENVQK + 3 Deamidated (NQ)
14	1211.6045	1210.5972	1210.5982	0	78	2.80E-03	R.FNDLFESALR.N
15	1222.649	1221.6417	1221.6506	0	43	1.10E+01	SNAFSLAPLFR
16	1232.5614	1231.5541	1231.5429	0	61	1.50E-01	K.AQQTADEANER.A
17	1247.5892	1246.5819	1246.6452	1	34	9.40E+01	MAAISRTLDNR
18	1262.6835	1261.6762	1261.6666	0	44	8.90E+00	NDVLELIQYR
19	1277.6857	1276.6784	1276.6776	0	86	6.20E-04	K.NLATTEGAVVFR.N
20	1306.6918	1305.6845	1305.683	0	89	3.30E-04	R.NGVQTFIVSWR.N
21	1389.6716	1388.6643	1388.6646	0	65	0.073	R.FMTNPELPAEPK.A + Oxidation (M)
22	1409.7524	1408.7451	1408.829	0	37	4.30E+01	QPVDLSLAQVVLK
23	1539.8851	1538.8778	1538.878	0	114	6.70E-07	K.AASLANGLLNIDLVR.L
24	1552.777	1551.7697	1551.7358	0	41	2.20E+01	FSDPAWSQNPLYK
25	1570.8582	1569.8509	1569.8555	0	86	5.80E-04	R.LPAALHGEFVELFK.S
26	1683.8495	1682.8422	1682.7426	1	34	1.10E+02	AKMLAGTDCTMESPR + Carbamidomethyl (C); Oxidation (M)
27	1702.8575	1701.8502	1701.8434	0	72	2.00E-02	K.STDNVTYLHQGIAQR.A
28	1722.9476	1721.9403	1721.9424	0	117	4.20E-07	K.QAAENTLNLNPVIGIR.G
29	1794.8827	1793.8754	1793.8869	0	31	2.30E+02	FLETDPAMISAETTLR
30	1939.993	1938.9857	1938.9871	1	45	1.00E+01	NVLLGQSELRPGDDDRR
31	2078.1133	2077.106	2077.1167	0	124	1.00E-07	K.SNPLNRPGALEVSGTPIDLK.Q
32	2149.0337	2148.0264	2148.0078	0	105	1.10E-05	K.HADSWWLHWQQWLAER.S
33	2165.0425	2164.0352	2164.1561	1	31	2.70E+02	MANLIYLTLEGKQQGLISR + Deamidated (NQ); Oxidation (M)
34	2181.021	2180.0137	2180.1225	1	21	2.40E+03	DKAIEAWLTHSAAPSLDSIR
35	2181.0264	2180.0191	2179.9341	1	23	1.60E+03	HVSDDDSYNMSTRSWQPR
36	2206.2073	2205.2	2205.1277	2	30	2.40E+02	DNQQALNSYLAGKIDAKNLK + 2 Deamidated (NQ)

a) The number of missed cleavage sites.

b) The score is the -log10(*P*) value, where P is the probability that the observed match is a random event. Individual ion scores of >56 indicate identity or extensive homology (P ≤ 0.05).

c) Expected score based on BLAST search.

d) The sequence between the peptides was identified by MS. The amino acid before the period at the N terminal and that after the period at the C terminal indicate the cleavage sites.

Supplementary Table 3. Amino acid alignment of peptides identified by MALDI-TOF MS in dominant HCPs with respective PHA synthase fusion protein and mapping of anti-PhaC1 antibody epitopes.

Band I (Amino acid coverage: 34%)
Reference sequence Ag-PhaC1$_{Pa}$

**1**	*MHLRRPGEEV*	*NLTTTTVDDR*	*RIATGKQNAT*	*AEGRAINR**RV***	***ENATAEGR**AI*
**51**	*NRRVENATAE*	*GRAINRRVES*	*SHSKETEAR**L***	***TATEDAAAR**A*	*QARADEAYRK*
**101**	*ADEALGAAQK*	*AQQTADEANE*	*RALRMLEKAS*	*RK*PRGSGGGH	MSQKNNNELP
**151**	**KQAAENTLNL**	**NPVIGIR**GKD	LLTSAR**MVLL**	**QAVRQPLHSA**	**RHVAHFSLEL**
**201**	**KNVLLGQSEL**	**RPGDDDRRFS**	**DPAWSQNPLY**	**KRYMQTYLAW**	**RKELHSWISH**
**251**	**SDLSPQDISR**	**GQFVINLLTE**	**AMSPTNSLSN**	**PAAVK**RFFET	GGK**SLLDGLG**
**301**	**HLAKDLVNNG**	**GMPSQVDMDA**	**FEVGKNLATT**	**EGAVVFRNDV**	**LELIQYRPIT**
**351**	**ESVHERPLLV**	**VPPQINKFYV**	**FDLSPDK**SLA	RFCLR**NGVQT**	**FIVSWR**NPTK
**401**	SQREWGLTTY	IEALKEAIEV	VLSITGSKDL	NLLGACSGGI	TTATLVGHYV
**451**	ASGEKKVNAF	TQLVSVLDFE	LNTQVALFAD	EKTLEAAKRR	SYQSGVLEGK
**501**	DMAKVFAWMR	PNDLIWNYWV	NNYLLGNQPP	AFDILYWNND	TTRLPAALHG
**551**	EFVELFKSNP	LNRPGALEVS	GTPIDLKQVT	CDFYCVAGLN	DHITPWESCY
**601**	KSARLLGGKC	EFILSNSGHI	QSILNPPGNP	KARFMTNPEL	PAEPKAWLEQ
**651**	AGKHADSWWL	HWQQWLAERS	GKTRKAPASL	GNKTYPAGEA	APGTYVHER

Band II (Amino acid coverage: 24%)
Reference sequence Ag-PhaC1$_{Pa}$

**1**	*MHLRRPGEEV*	*NLTTTTVDDR*	*RIATGKQNAT*	*AEGRAINRRV*	*ENATAEGRAI*
**51**	*NRRVENATAE*	*GRAINRRVES*	*SHSKETEARL*	*TATEDAAARA*	*QARADEAYRK*
**101**	*ADEALGAAQK*	*AQQTADEANE*	*RALRMLEKAS*	*RK*PRGSGGGH	MSQKNNNELP
**151**	**KQAAENTLNL**	**NPVIGIRGKD**	**LLTSARMVLL**	**QAVR**QPLHSA	R**HVAHFSLEL**
**201**	**KNVLLGQSEL**	**RPGDDDRR**FS	DPAWSQNPLY	KR**YMQTYLAW**	**R**KELHSWISH
**251**	SDLSPQDISR	**GQFVINLLTE**	**AMSPTNSLSN**	**PAAVK**RFFET	GGK**SLLDGLG**
**301**	**HLAK**DLVNNG	GMPSQVDMDA	FEVGK**NLATT**	**EGAVVFRNDV**	**LELIQYRPIT**
**351**	**ESVHERPLLV**	**VPPQINKFYV**	**FDLSPDK**SLA	RFCLR**NGVQT**	**FIVSWR**NPTK
**401**	SQREWGLTTY	IEALKEAIEV	VLSITGSKDL	NLLGACSGGI	TTATLVGHYV
**451**	ASGEKKVNAF	TQLVSVLDFE	LNTQVALFAD	EKTLEAAKRR	SYQSGVLEGK
**501**	DMAKVFAWMR	PNDLIWNYWV	NNYLLGNQPP	AFDILYWNND	TTRLPAALHG
**551**	EFVELFKSNP	LNRPGALEVS	GTPIDLKQVT	CDFYCVAGLN	DHITPWESCY
**601**	KSARLLGGKC	EFILSNSGHI	QSILNPPGNP	KARFMTNPEL	PAEPKAWLEQ
**651**	AGKHADSWWL	HWQQWLAERS	GKTRKAPASL	GNKTYPAGEA	APGTYVHER

Band III (Amino acid coverage: 30%)
Reference sequence Ag-PhaC1$_{Pa}$

**1**	*MHLRRPGEEV*	*NLTTTTVDDR*	*RIATGKQNAT*	*AEGRAINR**RV***	***ENATAEGR**AI*
**51**	*NRRVENATAE*	*GRAINRRVES*	*SHSKETEARL*	*TATEDAAARA*	*QARADEAYRK*
**101**	*ADEALGAAQK*	*AQQTADEANE*	*RALRMLEKAS*	*RK*PRGSGGGH	MSQKNNNELP
**151**	**KQAAENTLNL**	**NPVIGIRGKD**	**LLTSARMVLL**	**QAVRQPLHSA**	**RHVAHFSLEL**
**201**	**KNVLLGQSEL**	**RPGDDDRRFS**	**DPAWSQNPLY**	**KRYMQTYLAW**	**RKELHSWISH**
**251**	**SDLSPQDISR**	**GQFVINLLTE**	**AMSPTNSLSN**	**PAAVK**RFFET	GGK**SLLDGLG**
**301**	**HLAK**DLVNNG	GMPSQVDMDA	FEVGK**NLATT**	**EGAVVFRNDV**	**LELIQYRPIT**
**351**	**ESVHERPLLV**	**VPPQINKFYV**	**FDLSPDK**SLA	RFCLR**NGVQT**	**FIVSWR**NPTK
**401**	SQREWGLTTY	IEALKEAIEV	VLSITGSKDL	NLLGACSGGI	TTATLVGHYV
**451**	ASGEKKVNAF	TQLVSVLDFE	LNTQVALFAD	EKTLEAAKRR	SYQSGVLEGK
**501**	DMAKVFAWMR	PNDLIWNYWV	NNYLLGNQPP	AFDILYWNND	TTRLPAALHG
**551**	EFVELFKSNP	LNRPGALEVS	GTPIDLKQVT	CDFYCVAGLN	DHITPWESCY
**601**	KSARLLGGKC	EFILSNSGHI	QSILNPPGNP	KARFMTNPEL	PAEPKAWLEQ
**651**	AGKHADSWWL	HWQQWLAERS	GKTRKAPASL	GNKTYPAGEA	APGTYVHER

Band IV (Amino acid coverage: 22%)
Reference sequence Ag-PhaC1$_{Pa}$

**1**	*MHLRRPGEEV*	*NLTTTTVDDR*	*RIATGKQNAT*	*AEGRAINRRV*	*ENATAEGRAI*
**51**	*NRRVENATAE*	*GRAINRRVES*	*SHSKETEARL*	*TATEDAAARA*	*QARADEAYRK*
**101**	*ADEALGAAQK*	***AQQTADEANE***	***R**ALRMLEKAS*	*RK*PRGSGGGH	MSQKNNNELP
**151**	KQAAENTLNL	NPVIGIRGKD	LLTSARMVLL	QAVRQPLHSA	R**HVAHFSLEL**
**201**	KNVLLGQSEL	RPGDDDRRFS	DPAWSQNPLY	KRYMQTYLAW	RKELHSWISH
**251**	SDLSPQDISR	GQFVINLLTE	AMSPTNSLSN	PAAVKRFFET	GGKSLLDGLG
**301**	HLAKDLVNNG	GMPSQVDMDA	FEVGK**NLATT**	**EGAVVFR**NDV	LELIQYRPIT
**351**	ESVHERPLLV	VPPQINKFYV	FDLSPDKSLA	RFCLR**NGVQT**	**FIVSWR**NPTK
**401**	SQREWGLTTY	IEALKEAIEV	VLSITGSKDL	NLLGACSGGI	TTATLVGHYV
**451**	ASGEKKVNAF	TQLVSVLDFE	LNTQVALFAD	EKTLEAAKRR	**SYQSGVLEGK**
**501**	**DMAK**VFAWMR	PNDLIWNYWV	NNYLLGNQPP	AFDILYWNND	TTR**LPAALHG**
**551**	**EFVELFKSNP**	**LNRPGALEVS**	**GTPIDLK**QVT	CDFYCVAGLN	DHITPWESCY
**601**	KSARLLGGK**C**	**EFILSNSGHI**	**QSILNPPGNP**	**K**AR**FMTNPEL**	**PAEPK**AWLEQ
**651**	AGK**HADSWWL**	**HWQQWLAERS**	GKTRKAPASL	GN**KTYPAGEA**	**APGTYVHER**

Note:
Letters in 'Bold' represent peptides identified by MALDI-TOF MS
'Underlined' letters indicate linker
Letter in 'italics' indicate antigen fusion partner
'Highlighted' letters represent mapped anti-PhaC1 antibodies epitopes: Yellow, anti-PhaC1_1 (MSQKNNNELPKQAA); Green, anti-PhaC1_67 (QSELRPGDDDRRFS); and Blue, anti-PhaC1_529 (RSGKTRKAPASLGN).

Supplementary Table 3. (*Continued*)

Band V (Amino acid coverage: 1.6%)
Reference sequence Ag-PhaC1$_{Pa}$

**1**	*MHLRRPGEEV*	*NLTTTTVDDR*	*RIATGKQNAT*	*AEGRAINRRV*	*ENATAEGRAI*
**51**	*NRRVENATAE*	*GRAINRRVES*	*SHSKETEARL*	*TATEDAAARA*	*QARADEAYRK*
**101**	*ADEALGAAQK*	*AQQTADEANE*	*RALRMLEKAS*	*RK*PRGSGGGH	MSQKNNNELP
**151**	KQAAENTLNL	NPVIGIRGKD	LLTSARMVLL	QAVRQPLHSA	RHVAHFSLEL
**201**	KNVLLGQSEL	RPGDDDRRFS	DPAWSQNPLY	KRYMQTYLAW	RKELHSWISH
**251**	SDLSPQDISR	GQFVINLLTE	AMSPTNSLSN	PAAVKRFFET	GGKSLLDGLG
**301**	HLAKDLVNNG	GMPSQVDMDA	FEVGKNLATT	EGAVVFRNDV	LELIQYRPIT
**351**	ESVHERPLLV	VPPQINKFYV	FDLSPDKSLA	RFCLR**NGVQT**	**FIVSWR**NPTK
**401**	SQREWGLTTY	IEALKEAIEV	VLSITGSKDL	NLLGACSGGI	TTATLVGHYV
**451**	ASGEKKVNAF	TQLVSVLDFE	LNTQVALFAD	EKTLEAAKRR	SYQSGVLEGK
**501**	DMAKVFAWMR	PNDLIWNYWV	NNYLLGNQPP	AFDILYWNND	TTRLPAALHG
**551**	EFVELFKSNP	LNRPGALEVS	GTPIDLKQVT	CDFYCVAGLN	DHITPWESCY
**601**	KSARLLGGKC	EFILSNSGHI	QSILNPPGNP	KARFMTNPEL	PAEPKAWLEQ
**651**	AGKHADSWWL	HWQQWLAERS	GKTRKAPASL	GNKTYPAGEA	APGTYVHER

Band VI (Amino acid coverage: 9%)
Reference sequence Ag-PhaC1$_{Pa}$

**1**	*MHLRRPGEEV*	*NLTTTTVDDR*	*RIATGKQNAT*	*AEGRAINRRV*	*ENATAEGRAI*
**51**	*NRRVENATAE*	*GRAINRRVES*	*SHSKETEAR**L***	***TATEDAAARA***	*QAR**ADEAYRK***
**101**	***ADEALGAAQK***	***AQQTADEANE***	***R**ALRMLEKAS*	*RK*PRGSGGGH	MSQKNNNELP
**151**	KQAAENTLNL	NPVIGIRGKD	LLTSARMVLL	QAVRQPLHSA	RHVAHFSLEL
**201**	KNVLLGQSEL	RPGDDDRRFS	DPAWSQNPLY	KRYMQTYLAW	RKELHSWISH
**251**	SDLSPQDISR	GQFVINLLTE	AMSPTNSLSN	PAAVKRFFET	GGKSLLDGLG
**301**	HLAKDLVNNG	GMPSQVDMDA	FEVGKNLATT	EGAVVFRNDV	LELIQYRPIT
**351**	ESVHERPLLV	VPPQINKFYV	FDLSPDKSLA	RFCLR**NGVQT**	**FIVSWR**NPTK
**401**	SQREWGLTTY	IEALKEAIEV	VLSITGSKDL	NLLGACSGGI	TTATLVGHYV
**451**	ASGEKKVNAF	TQLVSVLDFE	LNTQVALFAD	EKTLEAAKRR	SYQSGVLEGK
**501**	DMAKVFAWMR	PNDLIWNYWV	NNYLLGNQPP	AFDILYWNND	TTRLPAALHG
**551**	EFVELFK**SNP**	**LNRPGALEVS**	**GTPIDLK**QVT	CDFYCVAGLN	DHITPWESCY
**601**	KSARLLGGKC	EFILSNSGHI	QSILNPPGNP	KARFMTNPEL	PAEPKAWLEQ
**651**	AGKHADSWWL	HWQQWLAERS	GKTRKAPASL	GNKTYPAGEA	APGTYVHER

Band VII (Amino acid coverage: 42%)
Reference sequence PhaC1$_{Pa}$-Ag

**1**	MSQKNNNELP	**KQAAENTLNL**	**NPVIGIRGKD**	**LLTSARMVLL**	**QAVRQPLHSA**
**51**	**RHVAHFSLEL**	KNVLLGQSEL	RPGDDDRR**FS**	**DPAWSQNPLY**	**KRYMQTYLAW**
**101**	R**KELHSWISH**	**SDLSPQDISR**	**GQFVINLLTE**	**AMSPTNSLSN**	**PAAVKR**FFET
**151**	GGKSLLDGLG	HLAK**DLVNNG**	**GMPSQVDMDA**	**FEVGKNLATT**	**EGAVVFRNDV**
**201**	**LELIQYRPIT**	**ESVHERPLLV**	**VPPQINKFYV**	**FDLSPDK**SLA	RFCLR**NGVQT**
**251**	**FIVSWR**NPTK	SQREWGLTTY	IEALKEAIEV	VLSITGSKDL	NLLGACSGGI
**301**	TTATLVGHYV	ASGEKKVNAF	TQLVSVLDFE	LNTQVALFAD	EKTLEAAKRR
**351**	SYQSGVLEGK	DMAKVFAWMR	PNDLIWNYWV	NNYLLGNQPP	AFDILYWNND
**401**	TTR**LPAALHG**	**EFVELFKSNP**	**LNRPGALEVS**	**GTPIDLK**QVT	CDFYCVAGLN
**451**	DHITPWESCY	KSARLLGGKC	EFILSNSGHI	QSILNPPGNP	KAR**FMTNPEL**
**501**	**PAEPK**AWLEQ	AGK**HADSWWL**	**HWQQWLAE**RS	GKTRKAPASL	GNKTYPAGEA
**551**	APGTYVHER**G**	**SVLAVAIDKR**	GGGGGLESGG	GGSGGGGSGG	GGSPG*SSHSK*
**601**	*ETEAR**LTATE***	***DAAAR**AQARA*	*DEAYRKADEA*	*LGAAQKAQQT*	*ADEANERALR*
**651**	*MLEKASRKNA*	*TAEGRAINR**R***	***VENATAEG**RA*	*INRRVENATA*	*EGRAINR**RVE***
**701**	***NLTTTTVDDR***	***R**IATGKQHLR*	*RPGEEV*		

Band VIII (Amino acid coverage: 32%)
Reference sequence PhaC1$_{Pa}$-Ag

**1**	MSQK**NNNELP**	**KQAAENTLNL**	**NPVIGIRGKD**	**LLTSARMVLL**	**QAVRQPLHSA**
**51**	**RHVAHFSLEL**	**KNVLLGQSEL**	**RPGDDDRRFS**	**DPAWSQNPLY**	**KRYMQTYLAW**
**101**	R**KELHSWISH**	**SDLSPQDISR**	**GQFVINLLTE**	**AMSPTNSLSN**	**PAAVKR**FFET
**151**	GGK**SLLDGLG**	**HLAKDLVNNG**	**GMPSQVDMDA**	**FEVGKNLATT**	**EGAVVFRNDV**
**201**	**LELIQYRPIT**	**ESVHERPLLV**	**VPPQINKFYV**	**FDLSPDK**SLA	RFCLR**NGVQT**
**251**	**FIVSWR**NPTK	SQREWGLTTY	IEALKEAIEV	VLSITGSKDL	NLLGACSGGI
**301**	TTATLVGHYV	ASGEKKVNAF	TQLVSVLDFE	LNTQVALFAD	EKTLEAAKRR
**351**	SYQSGVLEGK	DMAKVFAWMR	PNDLIWNYWV	NNYLLGNQPP	AFDILYWNND
**401**	TTRLPAALHG	EFVELFKSNP	LNRPGALEVS	GTPIDLKQVT	CDFYCVAGLN
**451**	DHITPWESCY	KSARLLGGKC	EFILSNSGHI	QSILNPPGNP	KARFMTNPEL
**501**	PAEPKAWLEQ	AGKHADSWWL	HWQQWLAERS	GKTRKAPASL	GNKTYPAGEA
**551**	APGTYVHERG	SVLAVAIDKR	GGGGGLESGG	GGSGGGGSGG	GGSPG*SSHSK*
**601**	*ETEARLTATE*	*DAAARAQARA*	*DEAYRKADEA*	*LGAAQKAQQT*	*ADEANERALR*
**651**	*MLEKASRKNA*	*TAEGRAINRR*	*VENATAEGRA*	*INRRVENATA*	*EGRAINRRVE*
**701**	*NLTTTTVDDR*	*RIATGKQHLR*	*RPGEEV*		

Note:
Letters in 'Bold' represent peptides identified by MALDI-TOF MS
'Underlined' letters indicate linker
Letter in 'italics' indicate antigen fusion partner
'Highlighted' letters represent mapped anti-PhaC1 antibodies epitopes: Yellow, anti-PhaC1_1 (MSQKNNNELPKQAA); Green, anti-PhaC1_67 (QSELRPGDDDRRFS); and Blue, anti-PhaC1_529 (RSGKTRKAPASLGN).

Supplementary Table 3. (*Continued*)

Band IX (Amino acid coverage: 32%)
Reference sequence $PhaC1_{Pa}$-Ag

**1**	MSQKNNNELP	**KQAAENTLNL**	**NPVIGIRGKD**	**LLTSARMVLL**	**QAVRQPLHSA**
**51**	**RHVAHFSLEL**	**KNVLLGQSEL**	**RPGDDDRRFS**	**DPAWSQNPLY**	**K**R**YMQTYLAW**
**101**	**RKELHSWISH**	**SDLSPQDISR**	**GQFVINLLTE**	**AMSPTNSLSN**	**PAAVK**RFFET
**151**	GGKSLLDGLG	HLAK**DLVNNG**	**GMPSQVDMDA**	**FEVGKNLATT**	**EGAVVFRNDV**
**201**	**LELIQYRPIT**	**ESVHERPLLV**	**VPPQINKFYV**	**FDLSPDK**SLA	RFCLR**NGVQT**
**251**	**FIVSWR**NPTK	SQREWGLTTY	IEALKEAIEV	VLSITGSKDL	NLLGACSGGI
**301**	TTATLVGHYV	ASGEKKVNAF	TQLVSVLDFE	LNTQVALFAD	EKTLEAAKRR
**351**	**SYQSGVLEGK**	**DMAK**VFAWMR	PNDLIWNYWV	NNYLLGNQPP	AFDILYWNND
**401**	TTR**LPAALHG**	**EFVELFKSNP**	**LNRPGALEVS**	**GTPIDLK**QVT	CDFYCVAGLN
**451**	DHITPWESCY	KSARLLGGK**C**	**EFILSNSGHI**	**QSILNPPGNP**	**K**AR**FMTNPEL**
**501**	**PAEPK**AWLEQ	AGKHADSWWL	HWQQWLAERS	GKTRKAPASL	GNKTYPAGEA
**551**	APGTYVHERG	SVLAVAIDKR	GGGGGLESGG	GGSGGGGSGG	GGSPG*SSHSK*
**601**	*ETEARLTATE*	*DAAARAQARA*	*DEAYRKADEA*	*LGAAQKAQQT*	*ADEANERALR*
**651**	*MLEKASRKNA*	*TAEGRAINRR*	*VENATAEGRA*	*INRRVENATA*	*EGRAINRRVE*
**701**	*NLTTTTVDDR*	*RIATGKQHLR*	*RPGEEV*		

Band X (Amino acid coverage: 32%)
Reference sequence $PhaC1_{Pa}$-Ag

**1**	MSQKNNNELP	**KQAAENTLNL**	**NPVIGIRGKD**	**LLTSARMVLL**	**QAVRQPLHSA**
**51**	**RHVAHFSLEL**	**KNVLLGQSEL**	**RPGDDDRRFS**	**DPAWSQNPLY**	**K**R**YMQTYLAW**
**101**	**RKELHSWISH**	**SDLSPQDISR**	**GQFVINLLTE**	**AMSPTNSLSN**	**PAAVK**RFFET
**151**	GGK**SLLDGLG**	**HLAK**DLVNNG	GMPSQVDMDA	FEVGKNLATT	EGAVVFRNDV
**201**	LELIQYRPIT	ESVHERPLLV	VPPQINKFYV	FDLSPDKSLA	RFCLR**NGVQT**
**251**	**FIVSWR**NPTK	SQREWGLTTY	IEALKEAIEV	VLSITGSKDL	NLLGACSGGI
**301**	TTATLVGHYV	ASGEKKVNAF	TQLVSVLDFE	LNTQVALFAD	EKTLEAAKRR
**351**	SYQSGVLEGK	DMAKVFAWMR	PNDLIWNYWV	NNYLLGNQPP	AFDILYWNND
**401**	TTR**LPAALHG**	**EFVELFKSNP**	**LNRPGALEVS**	**GTPIDLK**QVT	CDFYCVAGLN
**451**	DHITPWESCY	KSARLLGGKC	EFILSNSGHI	QSILNPPGNP	KARFMTNPEL
**501**	PAEPKAWLEQ	AGKHADSWWL	HWQQWLAERS	GKTRKAPASL	GNKTYPAGEA
**551**	APGTYVHERG	SVLAVAIDKR	GGGGGLESGG	GGSGGGGSGG	GGSPG*SSHSK*
**601**	*ETEAR**LTATE***	***DAAAR**AQARA*	*DEAYRKADEA*	*LGAAQK**AQQT***	***ADEANER**ALR*
**651**	*MLEKASRKNA*	*TAEGRAINR**R***	***VENATAEGR**A*	*INRRVENATA*	*EGRAINR**RVE***
**701**	***NLTTTTVDDR***	***R**IATGKQHLR*	*RPGEEV*		

Band XI (Amino acid coverage: 26%)
Reference sequence $PhaC1_{Pa}$-Ag

**1**	MSQKNNNELP	**KQAAENTLNL**	**NPVIGIR**GKD	LLTSARMVLL	QAVR**QPLHSA**
**51**	**RHVAHFSLEL**	**KNVLLGQSEL**	**RPGDDDRRFS**	**DPAWSQNPLY**	**K**R**YMQTYLAW**
**101**	**R**KELHSWISH	SDLSPQDISR	GQFVINLLTE	AMSPTNSLSN	PAAVKRFFET
**151**	GGKSLLDGLG	HLAKDLVNNG	GMPSQVDMDA	FEVGK**NLATT**	**EGAVVFRNDV**
**201**	**LELIQYR**PIT	ESVHERPLLV	VPPQINKFYV	FDLSPDKSLA	RFCLR**NGVQT**
**251**	**FIVSWR**NPTK	SQREWGLTTY	IEALKEAIEV	VLSITGSKDL	NLLGACSGGI
**301**	TTATLVGHYV	ASGEKKVNAF	TQLVSVLDFE	LNTQVALFAD	EKTLEAAKRR
**351**	SYQSGVLEGK	DMAKVFAWMR	PNDLIWNYWV	NNYLLGNQPP	AFDILYWNND
**401**	TTR**LPAALHG**	**EFVELFKSNP**	**LNRPGALEVS**	**GTPIDLK**QVT	CDFYCVAGLN
**451**	DHITPWESCY	KSARLLGGKC	EFILSNSGHI	QSILNPPGNP	KAR**FMTNPEL**
**501**	**PAEPKAWLEQ**	**AGKHADSWWL**	**HWQQWLAERS**	GKTRKAPASL	GNKTYPAGEA
**551**	APGTYVHERG	SVLAVAIDKR	GGGGGLESGG	GGSGGGGSGG	GGSPG*SSHSK*
**601**	*ETEAR**LTATE***	***DAAAR**AQARA*	*DEAYRKADEA*	*LGAAQK**AQQT***	***ADEANER**ALR*
**651**	*MLEKASRKNA*	*TAEGRAINR**R***	***VENATAEGR**A*	*INRRVENATA*	*EGRAINRRVE*
**701**	*NLTTTTVDDR*	*RIATGKQHLR*	*RPGEEV*		

Note:
Letters in ‘Bold’ represent peptides identified by MALDI-TOF MS
‘Underlined’ letters indicate linker
Letter in ‘italics’ indicate antigen fusion partner
‘Highlighted’ letters represent mapped anti-PhaC1 antibodies epitopes: Yellow, anti-PhaC1_1 (MSQKNNNELPKQAA); Green, anti-PhaC1_67 (QSELRPGDDDRRFS); and Blue, anti-PhaC1_529 (RSGKTRKAPASLGN).

Supplementary Table 4. 33 known and putative PHA synthases from bacterial human pathogens.

Organism	Taxonomy ID (NCBI)	Protein/Gene	Amino acid sequence
1: *A. baumannii*	575584	HMPREF0010_00690	MKRLKSLVSEQSQIKHLSTRLFRPQTLVLSQSTPFEVIGEFNQTRVRYYAATEKSFREPLVFVAPLAINMAIYDLYPYRSLIKYFQNAGFDVYLVDWGRLGFKDRHLNFLSFIEDFIPKAIELVRTHSGSDQISLHGWSMAGIFVTLYTAHNHPNYVKNLIVLGSPIDSYASGYIGKLYRTINNTIARNKKLQERIYSGLPKRLIHTPGILNSLGFKILDPKGWFDGHIQLLKNLNDLQFVQEHATLSSFLNNMIDYPGGINQDMLFNVWLQNPLRQGSIQLKDKKIELKNIDCSLLVGAGRSDQLVTADAAQPLSQLTSSQDVTFTLIPGGHLGLMSSQASAQEFWPKLATWLSERSTKI
2: *A. calcoaceticus*	871585	BDGL_001038	MKRLKSLVSEQSQIKHLSTRLFRPQTLVLSQSTPFEVIGEFNQTRIRYYAATQKQFKEPLVFVAPLAINMAIYDLYPYRSLIKYFQNAGFDVYLVDWGRLKFKDRHLNFLSFIEDFIPKAIQLVRTHSGSEQISLHGWSMAGIFVTLYTAHNHPNYVKNLIVLGSPIDSYASGYIGKLYRTINNAIGRNKKIQERIYSGLPKRLIHSPGILNSLGFKILDPKGWLDGHIQLLKNLNDLQFVQEHATLSSFLNNMIDYPGGINQDMLFNVWLQNPLKRGFIELKDKKIELKNIDCSLLVGAGRSDQLVTADAAQPLSQLTSSQDVTFTLIPGGHLGLMSSQASALEFWPQLAKWLTERSTQI
3: *A. radioresistens*	575589	HMPREF0018_01802	MFTLKARIRQQKTRFFHLSRRVLNPESLVLSQSTPFQVISKYHGSQLRYYAAAHKRYKEPLVFVAPLAVDMAIYDLYPYRSLVQHFQLQGFDVYLVDWGTFTFQDRYRDFLFFIDDCLPHYIKTVCEHSQSEKISLHGWSMGGIFALLYSALAKQSHVKNLIILGSPIDSYASGRIGKLFKTVNQLLTRHAKIRHSIENIPEGLIHTPGFINALGFKIIDPAGWLNSCIQLFKYIDNEKFLREHTTVQTFLNHMNDYPGAINKDMIFKVWLKNPLKTGSIDLKDRLIDLKNIECSLLLGAGTTDQIVTEAAIQPLSQLTNSADVSFTAIPGGHIGLMSSQASANEFWPKLTEWLVQRSSRIKDTL
4: *B. anthracis*	198094	PHAC	MTTFATEWEKQLELYPEEYRKAYRRVKRASEILLREPEPQVGLTPKEVIWTKNKTKLYRYIPKQEKTQRVPILLIYALINKPYIMDLTPGNSLVEYLVDRGFDVYMLDWGTFGLEDSHLKFDDFVFDYIAKAVKKVMRTAKSDEISLLGYCMGGTLTSIYAALHPHMPIRNLIFMTSPFDFSETGLYGPLLDEKYFNLDKAVDTFGNIPPEMIDFGNKMLKPITNFVGPYVALVDRSENERFVESWRLVQKWVGDGIPFPGESYRQWIRDFYQNNKLVKGELVIRGQKVDLANIKANVLNISGKRDHIALPCQVEALLDHISSTDKQYVCLPTGHMSIVYGGTAVKQTYPTIGDWLDERSK
5: *B. pertussis*	257313	PHBC	MNAHLSAAWPVPVSVAPDALAEIQADFSREWLRLCDEAKRGVLGAPADKRFAGAAWLDDRQRLLMAHAYLLSARAMARLVEAAQVSEPMRNRLRFSVMQWVDAMSPANFLAFNPDAQRAIVESAGRTLQEGMANLLNDIQRGRISQTDETQFEIGRNVATTPGHVVFENSLMQLIQYAPQTAKVCERPLVIVPPNINKYYILDLQPENSFVRYAVEQGHTVFIISWRNPLAADTDGVDTATWSEYLDDAVLKALAVASDISGQPQVNALGFCVGGTMLASALALAQVRGERPVASLTLLTSLLDFHDTGILKVFVDEAHALLRDHQYGQRGLMPARDLATTFSFLRPNELVWNYVVSNYLKGKTPPAFDLLFWNADSTNLPGPFFAWYFRNTYLENNLKVPGRARVAGVPLDLTRLDMPTYLYGSREDHIVPWPSAYASTQLLRGPMRFVLGASGHIAGVINPPAKQRRSYWVNESAGAVSHDLPGDPNAWLAGAVEHAGSWWPDWTSWLAGHGGKQVAAPAQAGNKRFRPIEPAPGRYVKVRAV
6: *B. cenocepacia*	216591	PHBC	MTASKNSSTSAHAGTSAGSTGFDPAAQPMQQMFESWLNAWRGFADPARAATASASVNPFATFQFPTSFPFQMPSMPDFGAMASPFAGLKLPVAAIPPERLQALQADYARDCMTLMQQAAAAKLESPELKDRRFSGDAWKASPAHAFAAAWYLLNARYLQELADALQTDPKTRERIRFTVQQWTAAAAPSNFLALNPDAQKSILETQGESLRQGMMNLLGDLQRGKISQTDESQFVVGKNLGCTEGSVVYENDLIQLIQYTPKTDKVFERPLLIVPPCINKFYILDLQPENSLVAHALSNGHQVFLVSWRNADASVAHKTWDDYMNEGLLAAIDAVQQISGREQINTLGFCVGGTMLATALAVLAARGEHPAASMTLLTAMLDFTDTGILDVFVDEAHVQMREQTIGGKNGAQPGLMRGVEFANTFSFLRPNDLVWNYVVDNYLKGRTPAPFDLLYWNSDSTSLPGPMYAWYLRHTYLENKLREPGALTVCGESVDLSLIDVPTFIYGSREDHIVPWQTAYASTSILSGPLKFVLGASGHIAGVINPPAKKKRSYWVNEGDLPESADDWFAAATEQPGSWWTTWVEWLDAYGGRKVAPPAQPGSAQFPVIEPAPGRYVLQRD
7: *B. mallei*	243160	PHBC	MTASKKSSTSSHTDTPQGRSTAGLAAQPMQQLFESWLGAWRSFADPARAAAGDAPSPSPSPSPFAAFQPPQPFAFAMPAMPPMPPMPDWSGAAASFAGLAPVASVPPARLQKLQADYSRDCLALIQQASAATPTVPELKDRRFSADAWKASPAHGFAAAWYLLNARYLQELADALETDPKTRERIRFTVQQWTAAASPSNFLALNPEAQKNLVETQGESLRLGMMNLLADMQRGKISQTDESQFVVGKNLAVTPGAVVYENDLIQLIQYTPTTATVFERPLLIVPPCINKFYILDLQPENSLVAHALSCGHQVFLVSWRNADASVAHKTWDDYIDEGLLAAIDVVQQVSGREQINTLGFCVGGTMLATALAVLAARGEHPAASMTLLTSMLDFSDTGILDVFVDEAHVQMREQTIGGKGGAPAGLMRGVEFANTFSFLRPNDLVWNYVVDNYLKGRTPAPFDLLYWNGDSTSLPGPMYAWYLRNTYLENKLREPDALTVCGEPVDLSRIDVPTFIYGSREDHIVPWQTAYASTSLLTGPLKFVLGASGHIAGVINPPAKRKRSYWSYGASAKELPESANDWLDAAVEHPGSWWPVWIEWLDQYGGKKVKPRAHLGCARFPVIEPAPGRYVLQRD
8: *B. mallei*	243160	PHAC	MDTRHAPESGAPDAPLPAHPPASYAPESPYRIFDLAKEASVAKLTSGLSPASLQLALADWLIHLAAAPGKRAELATLALRHAALLGQYLLEAATGRTPAAPAQPSPGDRRFRAGAWQLEPYRFWHQSFLLAEQWWRAATRDVPGVSPHHEDVVAFSARQMLDTFAPANYVATNPEIAQRTALTGGANLAQGVWNYLDDVRRLITKQPPAGAEQFELGRNLATTPGRVVFRNHLIELLQYSPTTPDVYAQPVLIVPAWIMKYYILDLSAHNSLIRYLVGEGHTVFCISWRNVDASDRDLSLDDYRKLGVMDALDTIGAIVPGEKIHATGYCLGGTLLSIAAAAMANTGDDRLASITLLAAQTDFAEPGELQLFIDDSEIHFLESMMWERGYLGAHQMAGSFQLLMSNDLIWSRVIHDYLLGERTPMIDLMAWNADSTRMPYRMHSEYLRHLFLDNDLATNRYVIDGQTVSVHNIRAPFFVVGTEHDHIAPWRSVYKIHYLSGSDVTFVLTAGGHNAGIVSEPGHAKRHYRMKMTAAAAPSISPDEWLAGATDFEGSWWPAWHAWLARHSSPQRVAPPPLGKPGAHTLGDAPGTYVFQK
9: *B. multivorans*	395019	BMUL_1483	MTASKNSSTSAAAGTSAGNTGFGSAAQPMQQMFEAWLNAWRDFADPARAATASPAVNPFASFQFPKSFPFQMPSMPDFGAMASPFAGLTLPVAAIPPERLQKLQADYARDCVALMQQAAAAKLEAPELKDRRFSGDAWKASPAHAFAAAWYLLNARYLQELADALETDPKTRERIRFAVQQWTAAAAPSNFLALNPDAQKSILETQGESLRQGMMNLLGDLQRGKISQTDESQFVVGKNLGCTEGAVVYENDLIQLIQYTPKTAKVFERPLLIVPPCINKFYILDLQPENSLVAHAVSSGHQVFLVSWRNADASVAHKTWDDYMNEGLLAAIDAVQQVSGREQINTLGFCVGGTMLATALAVLAARGEHPAASMTLLTAMLDFSDTGVLDVFVDEAHVQMREQTIGGKNGTPPGLMRGVEFANTFSFLRPNDLVWNYVVDNYLKGRTPAPFDLLYWNSDSTSLPGPMYAWYLRNTYLENKLREPGALTVCGEPVDLSRIDVPTFIYGSREDHIVPWQTAYASTSILTGPLKFVLGASGHIAGVINPPAKKKRSYWVNDDDLPSAADDWFAGATEHPGSWWTTWIEWLDQYGGRKVAAPAELGSAQFPVIEPAPGRYVLQRD
10: *B. multivorans*	395019	BMULJ_01759	MTASKNSSTSAAAGTSAGNTGFGSAAQPMQQMFEAWLNAWRDFADPARAATASPAVNPFASFQFPKSFPFQMPSMPDFGAMASPFAGLTLPVAAIPPERLQKLQADYARDCVALMQQAAAAKLEAPELKDRRFSGDAWKASPAHAFAAAWYLLNARYLQELADALETDPKTRERIRFAVQQWTAAAAPSNFLALNPDAQKSILETQGESLRQGMMNLLGDLQRGKISQTDESQFVVGKNLGCTEGAVVYENDLIQLIQYTPKTAKVFERPLLIVPPCINKFYILDLQPENSLVAHAVSSGHQVFLVSWRNADASVAHKTWDDYMNEGLLAAIDAVQQVSGREQINTLGFCVGGTMLATALAVLAARGEHPAASMTLLTAMLDFSDTGVLDVFVDEAHVQMREQTIGGKNGTPPGLMRGVEFANTFSFLRPNDLVWNYVVDNYLKGRTPAPFDLLYWNSDSTSLPGPMYAWYLRNTYLENKLREPGALTVCGEPVDLSRIDVPTFIYGSREDHIVPWQTAYASTSILTGPLKFVLGASGHIAGVINPPAKKKRSYWVNDDDLPSAADDWFAGATEHPGSWWTTWIEWLDQYGGRKVAAPAELGSAQFPVIEPAPGRYVLQRD
11: *B. pseudomallei*	272560	PHBC	MQQLFESWLGAWRSFADPARAAAGDAPSPSPSPSPFAAFQPPQPFAFAMPAMPPMPDWSGAAASFAGLAPVASVPPARLQKLQADYSRDCLALIQQASAATPTVPELKDRRFSADAWKASPAHGFAAAWYLLNARYLQELADALETDPKTRERIRFTVQQWTAAASPSNFLALNPEAQKNLVETQGESLRLGMMNLLADMQRGKISQTDESQFVVGKNLAVTPGAVVYENDLIQLIQYTPTTATVFERPLLIVPPCINKFYILDLQPENSLVAHALSCGHQVFLVSWRNADASVAHKTWDDYIDEGLLAAIDVVQQVSGREQINTLGFCVGGTMLATALAVLAARGEHPAASMTLLTSMLDFSDTGILDVFVDEAHVQMREQTIGGKGGAPAGLMRGVEFANTFSFLRPNDLVWNYVVDNYLKGRTPAPFDLLYWNGDSTSLPGPMYAWYLRNTYLENKLREPDALTVCGEPVDLSRIDVPTFIYGSREDHIVPWQTAYASTSLLTGPLKFVLGASGHIAGVINPPAKRKRSYWSYDASAKELPESANDWLDAAVEHPGSWWPVWIEWLDQYGGKKVKPRAHLGCARFPVIEPAPGRYVLQRD
12: *B. pseudomallei*	272560	BPSS1954	MDTRHAPESGAPDAPLPAHPPASYAPESPYRIFDLAKEASVAKLTSGLSPASLQLALADWLIHLAAAPGKRAELATLALRHAALLGQYLLEAATGRTPAAPAQPSPGDRRFRAGAWQLEPYRFWHQSFLLAEQWWRAATRDVPGVSPHHEDVVAFSARQMLDTFAPANYVATNPEIAQRTALTGGANLAQGVWNYLDDVRRLITKQPPAGAEQFELGRNLATTPGRVVFRNHLIELLQYSPTTPDVYAQPVLIVPAWIMKYYILDLSAHNSLIRYLVGEGHTVFCISWRNVDASDRDLSLDDYRKLGVMDALDTIGAIVPGEKIHATGYCLGGTLLSIAAAAMANTGDDRLASITLLAAQTDFAEPGELQLFIDDSEIHFLESMMWERGYLGAHQMAGSFQLLMSNDLIWSRVIHDYLLGERTPMIDLMAWNADSTRMPYRMHSEYLRHLFLDNDLATNRYVIDGQTVSVHNIRAPFFVVGTEHDHIAPWRSVYKIHYLSGSDVTFVLTAGGHNAGIVSEPGHAKRHYRMKMTAAAAPSISPDEWLAGATDFEGSWWPAWHAWLARHSSPQRVAPPPLGKPGARTLGDAPGTYVFQK
13: *L. pneumophila*	272624	lpg0599	MLSGARMTHDTELSELMQAVAKKSLQIMTDFKEKPIPISSLVSQYIDLTEHFQNLIAVILKNPEKVWQMQLNYLEDALSLAQAQFNYWLEGKPLPINDQRFNGEDWINNPFFNLLSQHYLLANEHMNSLLENMEYGDENLAKRVRFFTRQYLDALSPANFIHTNPQLMAETLQSHGKNLLRGLHNLLSDVEAGSSRLIIKMTDTEAFKIGENLATTPGKVIFRNSMMELIQYCPRTTKVKSIPLLIVPPWINKYYILDLSPHNSLIRWLVEQGITVFIISWVNPDETYANKSFYDYLNEGPREAIAVIQKQLRVKQVNTLGFCIGGTLLASLLAYNKATKDHSIRSATFLAAMIDFSDPGDIAVFIDEQQINKLEEEMKSKGYLAGKFMASSFNSLRANDLIWSFFIKNYLRGKSPVPFDILYWNADSTNMPATMHSQYLRWMYLHNNLIKPGKIRLNHIPIDVTNIDIPTFFLSTQKDHIAPWKTTYKGFELMKGPKRFVLGGSGHIAGIINPPTQQKYGYRTNNSMDLSAEQWFEKSKEHSGSWWPEWLNWLKLHSGRLINSPDINHLPFAPIMDAPGSYVLKK

Supplementary Table 4. (*Continued*).

14: *L. pneumophila*	272624	lpg1058	MSAMKGLEKQCCPKKRILTQSQELQEGVDFSCCAPADRGTSDNFFPFFTRLVQANLAKWTAGISPAAIGSSYSTWLWQLAQSPGVLWELAFYPVFHAKDCINNIVCVERAADGKDVRFKKDSWQPMPWRLFAEGFLQMEDWWRRATTDVPGLPNQVERTVSFWARQCLDALSPSNFVWSNPDLFHEAMRTGGLNLIQGGQIALEDWLEKLTGAPPTGSEHFIPGKQVAITPGRVVFQNHLIELIQYEAQTKTVYKEPILILPAWIMKYYILDLSPHNSLVKWLVSQGHTVFIISWRNPDKEDQDLGMDDYYRQGAMAAIDAVSTLFPETKINLMGYCLGGTLAMITAAAMGRDKDERLNSLTLLAAQGDFTEAGELMLFVTESQVDFLKSMMREQGYLDTKQMAGSFQMLRAYDLIWSKMVQDYMHGMRRGMIDLTAWNADATRMPYKMHSEYLEKLFLRNDFAEGRYTVEGKPVAAENIKLPVFAVSTEKDHVAPWQSVYKIHLMTEGDVTFVLTGGGHNAGIISEPGHPGRSYRVHEQKQGEAYLNPESWLAMAERREGSWWREWNEWLVQQNTKKRIASSVMNPSLPEAPGTYVLQK
15: *L. pneumophila*	272624	lpg1097	MNGLEKQCCPKDVILTQQEKASESLSVCCAPSILGASDSFFSFFHKLFQANLAKMTLGISPAALGSSYSTWLWQLAQSPEVLWELALYPFLHANDCINNIICVERAADGKDVRFKKDSWQPMPWRLLAEGFLQVEDWWRRATNIPGLPRQVERTVSFWVRQCLDSVSPSNFVWSNPDLFHEAIRTNGLNLLKGSQIAMEDWLEKLTGAPPTGSENFIPGKDVAVTAGRVVFQNHLIELIQYEAQTKTVYKEPILILPAWIMKYYILDLSPHNSLVKWLVKQGHTVFIISWRNPDKEDCDLGMDDYYRLGAMAAIDMVSTILPETKINLMGYCLGGTLAMITASMMGRYEDERLNCLTLLAAQGDFSEAGELMLFVNESQVDFLKSMMWEQGYLDTKQMAGSFQMLRAYDLIWSKMVQDYMHGMRRGMIDLTAWNADATRMPYKMHSEYLEKLFLNNDFAEGRYMVEGKSVAAENIQCPIFAVSTKKDHVAPWQSVYKIHLMTEENVTFVLTGGGHNAGIISEPGHSGHAYHVHERKKGDAYLNPTNWLEIAEKREGSWWREWHDWLVQQSTKKRILPPVLNTSLPSAPGTYVLQK
16: *L. pneumophila*	272624	lpg2260	MSFMECCYTIFYKSNTYRMQGMKKTNEKPINKTNEMPASSKKEIVSPLEQTPASEQSDPIFRFIDKLYQANLGKLTAGISPAALGTAYYSWFAQLLQSPGSMLRLASYPLLHANDYLSNLFKYDKPRDGKDVRFHTDNWSYYPWRLWAEQFLQFEDWCLQASSKVPGIPLHVKRTVTFSTRQILDALSPSNFVLTNPDLLQETIRSNGQNLIRGTELAFQDFVEKITGSPPAGVENFIPGKQVAITKGKVVYSNHLIELIQYTPQTEKVYKEPILILPAWIMKYYILDLLPENSLVNWLVRQGHTVFIVSWRNPTKEDRNLGLDDYYKLGAMDAINAVSNAIPHTKIHLMGYCLGGTLALLTAAAMAHDHDNRLKTLSLLAAQGDFIDAGELLLFITKSEVSFLKSMMWEQGYLDTKQMSGTFQMLRSYDLIWSKMVQDYMHGTQRGMIPLLAWNADATRMPYKMHSEYLEKLFLNNDFAEGRFILDGKPVVGENIRIPAFVVSTEKDHVAPWKSVYKTHLLINSDITFVLTNGGHNAGIVSEPGHEGRYYRIRERKMDSTYLDPTTWVKRAELREGSWWIAWHDWLVNHSSQKQVSAPKLDKKLPNAPGKYVLQK
17: *L. borgpetersenii*	355276	LBL_2592	MIAILKNRRSPFYLATTFFILLFFALFASTLAFLFTGILILILIIHPLLLNWIGKLYGQEDIADEVHFAKTKDGWNIALHRHIPPQQNPQLAPVLVVHGIATNKFMVDLDRRHSLPYYLKLRGYDVFAVSLRGCGRSYHESPTRYEDFTFDDIVKYDIPAMFEKVKKITGSERVSYVGHSMGAMILYSHFCMSERKKDTEDIAAFVSLGGPGNLNHIGITLIGLLSRFPRARKMLDLKFGASILAPLAGELYTPIDEILYNPKVTSSKTVKKIMKNAIENIADGVTEQFMHWIETKRMHSLNGFYDYIRLQKNISVPALFIAGEKDVIATPEAVHSVYENASSKKKEFRVISKANGSSDDYGHACLVMGDRAEDDVFQYVESFLKKHGLRSQPGIMTKIKEGILSAFRR
18: *L. interrogans*	189518	LA_2034	MATTFLILLLFALFASTLAFIFTGVLILILLIHPLLLNWIGKLYGQEDIADEVHFAKTKDGWNLALHRHVPIQPNPQLAPVLVVHGIATNKFVMDLDRRHSLPYYLKLRGYDVFAVSLRGCGRSYHESPTRYEDFTFDDIVKYDVPAMIEKVKKITGSDRISYVGHSMGAMILYSHFCISEHKKDVEDIAAFVSLGGPGNLNHIGITLIGMLSRFPRARKMLDLKFGASILAPLAGELYTPIDEVLYNPNTTSSRTVKKIMKNAIENVSDGVTEQFMHWIETKRMHSLNGFYDYVQLQKKISVPSLFIAGEKDVIATPESVRSVYEKASSRKKEFRVISKANGASDDYGHACLVMGDRAEDDVFQHVESFLKKHGLRDQPGIGTKIKEGILSALFRFRG
19: *M. abscessus*	561007	MAB_2348	MAFNISSLTKPVARLAATAQNGLEVLRLGGLETGSTASSHQIVESVPMYRLRRYFAPGAGTEDAGPVVLMVHPMMMAADMWDVTQDGGAVGILHRAGIDPWVIDFGSPDRMAGGMERTLSDHVVAVSDAIETVHRITGRQIHLAGYSQGGMFCYQTAALRKSRTIASIITFGSPVDANAAMPMGMPAGLSADIAEFMADHVFSRFSIPAWSARIGFQMLDPVKTIKGRLDFLRQLHDRDALLPREQQRKFLANEGWIAWSGPAIAELLRQFVVHNRMTTGGFTVNDRVVTLSDITCPVLAVVGEVDDIGQPASVRGILRAAPKADVYEYLIRAGHFGLVVGSTAVAQTWPTVSQWVQWREGEAAKPPSVDLMYEHEAGQLDRGGVPLASRVAHGLSTTTEVAITAARTAGAAAAAANKSVKSIAVEAVRTLPRLTRLGQIHDHTRISMGRLMTEQARRTPHGECFLFDGRVHTYEAVDRRINNVVKGLIEVGVRQGVRVGILMETRPSALVAIAALSRLGAVAVLLPPDADLEVAVKLGEISELLTDPPNLPAAQDLPVHVLVLGGGESRDLSIPDDGSTIDMEKIDPDAVELPGWYRPDPGQARDLAFVMFSGAGSKLLPKQITNHRWALSAFGTASAAALSSNDTVYCLTPMHHQSGLLVSIGGSVAGGARIALSRGLDPDRFVQEIHQYGVSVVSYTWAMMHEVIDDPALALGAHHPVRLFIGSGMPAGLWRRVTEKFDPAHVVEFFATTDGEAVLANVSGTKVGSKGRPLPGGGKVRLAAYDPVEDVIIEGEDGFVQIAEPGEVGLLLAKPPGDVDPTAAVRRGVFAPGDTWVSSEFLFRRDEDGDFWMLDGRGTAIRTAHGVVYAEATSNALGALGAIDLVATYPVETGETTVAVTAVVLRPGEALSPADLAEAFAAVAISERPDIIKVVPNLPLSASYRPSTTHLRASGLPKPGRQTWHLDPESGAYHRLTAATYEALRGAVL
20: *M. avium*	262316	MAP1389	METGTVASPSQIVESVRMYKLRRYFPPDSRPGQPPVGPPVLMVHPMMMSADMWDVTRDEGAVGILHAHGLDPWVIDFGEPDKVEGGMRRTLTDHIVALSQAIDTVKDVTGADIHLVGYSQGGMWCYQVAAYRRSKSLASIVTFGSPVDTLAALPMGIPANFAAPAANFMADHVFSRLAIPSWMARTGFQMLDPLKTAKARVDFLRQLHDREALLPREQQRRFLEREGWIAWSGPAISELLKQFIAHNRMMTGGFAVNGQMVTLTDITCPVLAFVGEVDDIGQPASVRGIRRAAPDAEVYECTIRTGHFGLVVGSKAAQHSWPTVAAWVKWLSTGGDKPTGIDPMADQPAEHTDSGVALSSRIAHELGEASEAAIGLVRGAANAVVTANKSVRTLAVETARTLPRLVRLGQINDHTRISLGRIIEEQAHDAPQGEFLLFDGRVHTYEAVNRRINNVVRGLIEVGVRQGDRVGVLMETRPSALVAIAALSRLGAIAVVMRPDADLAASVRLGGATEILTDPTNLESVLASDRQLLRQVLVLGGGEARDLHLPEDSAEQPYVIDMEKIDPDAVELPGWYRPNPGLARDLAFIAFSAAGGELVAKQITNYRWAVSAFGTASTAALDRRDTVYCLTPLHHESALLVSLGGAVVGGTRIALSRGLDRDRFVQEVRQYGVTVVSYTWAMLREIVDDPAFVLHGNHPVRLFIGSGMPTGLWGRVVEAFAPAHVVEFFATTDGQAVLANVSGAKVGSKGRPLPGAGRIELGAYDTEHDLILENDRGFVQIAEPHQVGVLLAASNGPIDPSASVKRGVFAAGDTWISTEYLFYRDDDGDYWLAGRRGSVVHTPRGVVYAEPVTDALGCINGVDLAVTYNVPVGGHEVAVSAVTLLPGASITAADLTEACAKIPIGLGPDIVCVVPEMNLSATYRPTVSALRAAGIPKAGRQVWYFDAESGQYRRLTPAARAELSGGRS
21: *M. kansasii*	557599	MKANA1_01010 0013815	MAESPKPAAAPDELAAPLDLLLTSATRPFASRMMPDATWARLGANLAQRPGAVAGRTATLARELGSIAAGKSHRAPGRADKRFGDVAWQQNPLLHRVMQAYLAGAETAEGLLADAELDWRDQEKMQFVVDNLVEGLAPSNNPLISPLGWKALIDTGGLSAVRGLRAFVRDMLSKPRVPSMVEPDAFVVGETVAITKGAVVLQTSMFELIQYTPQTAKVRSIPLLMVPPVINKFYIMDIAPGRSMIEYFLQQGQQVFAISWRNPQARHRDWGFDAYGGAIVEAMDAVQNIAGTDSVHLMASCSGGIIAAMTAAHLAHIGEADRVAGLTLAVTVLDETRAGFAAAAMSDRAAQTAIRVSARKGYLDGRDMAEMFAWLRPTDLVWRYWVNNYVQGRKPAAFDVLFWNADTTRMAAALHRDMVLMGLRNALVTPGAVTMLGSPVDLADITSDAYVIGGVADHISPWQATYRSARLLGSKDNRYVLSTSGHIAALVNPPGNPKASFRTGLVGAEKPEEWLESAQQSAGSWWPDYVSWLAERSGPEVDAPKALGGQGLPPLGPAPGTYVKEQ
22: *M.leprae*	272631	ML1346	MDLNFSIVTRPVERLVATAQNGLEVLRLGGLETGSFPSPSQIVESVPMYKLRRYFPPGNRPGQPLLGAPVLMVHPMMMSADMWDVTREEGAVGILHVRGLDPWVIDFGSPDKVEGGMRRNLADHIVALSEAVDTVKEVTGNDVHLVGYSQGGMFCYQAAAYRRSENIASIVAFGSPVDTLAALPMGIPPNFGVVLANFMADHVFNRLDIPSWLARAGFQMLDPLKTVKARVDFVRQLHNREALLSREQQRRFLESEGWIAWSGPAISELLKQFIAHNRMMTGGFAVNGQMVTLTDITCPVLAFVGEVDDIGQPASVRGIRRAAPNAEVYESLIRTGHFGLVVGSRAAQQSWPTVAEWVCWLAANANKPANIHIMPDQPVEHTASGVAISSRLAHGLGEVSETALALARGVADAIVAANRSVHTLAVETVRTLPRLARLGQLNDHTRISLGRIIGEQAHDAPRGEFLLFDGRVHTYEAVDRRVNNVVRGLIAVGVRQGDRVGVLMETRPSALVAIAALSRLGAVAVMMRPDADLAASVRIGGATKILTDPANLGVVLAYGRQLTGQVLVLGGGESRDLHLPEDALQQNQVIDMEKIDPNAVDLPAWYRPNSGLARDLAFIACSTVGGELVAKQITNYRWAVSAFGTASTAALDRRDTVYCLTPLHHESALLVSLGGAVVGGARIALSRGLCSNRFVHEVRQYGVTVVSYTWAMLRELVDDPAFVLHGNHPVRLFMGSGMPTGLWERVVEAFAPAHVVEFFATVDGQAVLANVSGAKIGSKGRPLPGAGHVELGAYDAEQDLILENDRGFVQVADVNQIGVLLAASRGPIDPTASVKRGVFAPADTWIATEYLLRRDYDGDYWLAGRRSSVVRTARGLVYTEPVTDALGFITGVDLAATYSVAVDDRELAVSAVTLLPGAAITAADLTEAVASMPVGLGPDIVHVVPELTLSATYRPIVGALRTAGIPKTGRQVWYFDSASNQFRRMTPGVRAELAGKHTHTHA
23: *M. tuberculosis*	83332	MT1723	MVDLNFSMVTRPIERLVATAQNGLEVLRLGGLETGSVPSPSQIVESVPMYKLRRYFPPDNRPGQPPVGPPVLMVHPMMMSADMWDVTREDGAVGILHASGLDPWVIDFGSPDEVEGGMRRNLADHIVALSEAVDTVKDATGHDVHFVGYSQGGMFCYQAAAYRRSKDIASVVAFGSPVDTLAALPMGIPANMGAAVADFMADHVFNRLDIPSWMARMGFQMMDPLKTAKARVDFVRQLHDREALLPREQQRRFLESEGWIAWSGPAISELLKQFIAHNRMMTGGFAISGQMVTLTDITCPILAFVGEVDDIGQPASVRGIRRAAPNSEVYECLIRAGHFGLVVGSRAAQQSWPTVADWVRWISGDGTKPENIHLMADQPAEHTDSGVAFSSRVAHGIGEVSEAALALARGAADAVVAANRSVRTLAVETVRTLPRLARLGQLNDHTRISLGRIIDEQAHDAPKGEFLLFDGRVHTYEAVNRRINNVVRGLIAVGVRQGDRVGVLMETRPSALVAIAALSRLGAVAVVMRPDTDLSASVRLGRVTEILTDPTNLDAARQLPGQVLVLGGGESRDLDLPADALEQGQVIDMEKIDPDAVELPAWYRPNPGLARDLAFIAFSSADGDLVAKQITNYRWAVSAFGTASTAALGRRDTVYCLTPLHHESALLVSLGGAVVGGTRIALSRGLRPDRFVAEVRQYGVTVVSYTWAMLRDVVDDPAFVLHGNHPVRLFIGSGMPTGLWERVVEAFAPAHVVEFFATTDGQAVLANVAGAKIGSKGRPLPGAGRVELGAYDAEHDLILENDRGFVQVAGVNQVGVLLAQSRGPIDPTASVKRGVFAPADTWISTDYLFWRDDDGDYWLAGGRGSVVRTARGMVYTEPVTNALGLITGVDLAVTYGVLVRGRHVAVSAVTLLPGATITAADLTEAVASMPVGLGPDIVHVVPQLTLSGTYRPTVSALRANGIPKAGRQAWYFNSGGNEYRRLTPAVRTELTGQHRRGNA

Supplementary Table 4. (*Continued*).

24: *N. farcinica*	247156	NFA_45720	MSLADTLTLAARNAWALTFGPGVEAPEPTRSTVLWDAAHRELRRFERDEARDGAAAEGADPVLLVPPLAAPASCFDLRPDQSLARFLLGTGRTPYVVDYGEITFADRRMGFEDWINDILPEAVLRTSADRDG<br>AAVDLVGWSLGGTLALLTAAAHPQLPIGSITAVGSPLDYDRMTGMPQVRAVAKLDGGLAVSTAVRAAGGIPAPLTRAAYRVTAWNRELTRPLFVASNIARTEALAKMESIDRFMAQMPGYPGRFYGQLWGR<br>LILNNDIGRGVLRLGGREIALAAVTAPVLLVGGPADVITPAPAVEAGTRTLTGAAFVRYETAPGSHLGILTGETARETTWTYLDEFLTEAAAVRESVS
25: *P. aeruginosa*	208964	PhaC1	MSQKNNNELPKQAAENTLNLNPVIGIRGKDLLTSARMVLLQAVRQPLHSARHVAHFSLELKNVLLGQSELRPGDDDRRFSDPAWSQNPLYKRYMQTYLAWRKELHSWISHSDLSPQDISRGQFVINLLTEAM<br>SPTNSLSNPAAVKRFFETGGKSLLDGLGHLAKDLVNNGGMPSQVDMDAFEVGKNLATTEGAVVFRNDVLELIQYRPITESVHERPLLVVPPQINKFYVFDLSPDKSLARFCLRNGVQTFIVSWRNPTKSQRE<br>WGLTTYIEALKEAIEVVLSITGSKDLNLLGACSGGITTATLVGHYVASGEKKVNAFTQLVSVLDFELNTQVALFADEKTLEAAKRRSYQSGVLEGKDMAKVFAWMRPNDLIWNYWVNNYLLGNQPPAFDILYW<br>NNDTTRLPAALHGEFVELFKSNPLNRPGALEVSGTPIDLKQVTCDFYCVAGLNDHITPWESCYKSARLLGGKCEFILSNSGHIQSILNPPGNPKARFMTNPELPAEPKAWLEQAGKHADSWWLHWQQWLAE<br>RSGKTRKAPASLGNKTYPAGEAAPGTYVHER
26: *P. aeruginosa*	208964	PhaC2	MREKQESGSVPVPAEFMSAQSAIVGLRGKDLLTTVRSLAVHGLRQPLHSARHLVAFGGQLGKVLLGDTLHQPNPQDARFQDPSWRLNPFYRRTLQAYLAWQKQLLAWIDESNLDCDDRARARFLVALLSD<br>AVAPSNSLINPLALKELFNTGGISLLNGVRHLLEDLVHNGGMPSQVNKTAFEIGRNLATTQGAVVFRNEVLELIQYKPLGERQYAKPLLIVPPQINKYYIFDLSPEKSFVQYALKNNLQVFVISWRNPDAQHREW<br>GLSTYVEALDQAIEVSREITGSRSVNLAGACAGGLTVAALLGHLQVRRQLRKVSSVTYLVSLLDSQMESPAMLFADEQTLESSKRRSYQHGVLDGRDMAKVFAWMRPNDLIWNYWVNNYLLGRQPPAFDIL<br>YWNNDNTRLPAAFHGELLDLFKHNPLTRPGALEVSGTAVDLGKVAIDSFHVAGITDHITPWDAVYRSALLLGGQRRFILSNSGHIQSILNPPGNPKACYFENDKLSSDPRAWYYDAKREEGSWWPVWLGWL<br>QERSGELGNPDFNLGSAAHPPLEAAPGTYVHIR
27: *R. equi*	685727	REQ_24810	MMQVTVSCWGSALVSLGSEYVVGSLRRAVATAQNGLEVVRLGGLETGATPSPFQIVERAPMYRLRRYFADTEPAEAGPPIVLVPPMMMSADVYDVTRDQGAVGILHEMGLDPWVVDFGSPDAEEGGWNR<br>TLADHIVAISEIVDRVHEHTGRDVHLSGYSQGGMFCYQAAAYRRCRNIASLITFGAPVDTLAALPFNIPAGLATKGADLLADHVFNRLSIAGWMARTGFQLLDPVKTAKSRFDFLRQLHDRDALLPREQQRRFL<br>AQDGWVAWSGPAVAELLKQFIVHNRMMTGGFVIKDTAVSLAELSCPILAFVGEVDDIGQPLAVRGIKRAAPRAEVFESTLRAGHFGLVVGSAAASRTWPTTGEWVRWREDMGPRPEAVDVMIHDEPSGQD<br>SGVSLTNRITHTVASIAEVGVGVGKGIADFATDTVRGTREISIEAARALPRLARLGQLQPHSRISLGLLLAEQGRQAPNGECFLFDDRVHTNAAVNTRIDNVVRGLVHAGVRPAAHVGVLMATRPSALVAIAALS<br>RLGAVAVLLPPGGDLDEAVRLGRVDRIVVDPDHLEDAVATGQKVLVLGGGEFRGLAVELGPDVIDLEQVDPDAVTLPGWYRPDPGLARELAFVIFSGSCGNLEAKYITNFRWALSAFGTASAASLSRSDTVY<br>CLAPLHHSSGLMATLGGAIAGGARIALSRGLDPDRFAEEVRRYGVTVVSYTWTMMREILDAKSLPLEEGHPIRLFIGSGMPPGLWRRISSRFAPARVLEFYASTEGDVVLVNVSGAKVGSKGRRLPGSAEVR<br>VGAYDPIEGRFLEDARGFVRECEDDEVGLLLGRPGARTEAVPGVMRGVFAAGDAWVPTENLFRRDADGDFWLVDHKRTVVDTVRGPVFTQPIVDLLGEMPQVDLAVAYGVPTGEHQVPVAAITVHDGRVP<br>SAAEVTAALGQLPIEQCPDIVHVVDRIPLGPSYRPQATELQAAGLPKPSARSWCLDAETGRYKRFTKAAAARYGGSPEGPGGVPA
28: *R. prowazekii*	272947	E.RP820	MYNITHETIINNFKEIANKYQELILHFMQGKGSMIPRSLIDSDKNIIIVSCIIEQFCKNPQKFCQLNIEYIEKLRELTTNSFAKFVGNTSKDVFSTDNRDKRFKDALWEDNVYFHFVKQYYLLSAEWIKKNIEQYELSH<br>DLKQHLEFTTKHFIDAFAPSNFAFCNPKVLRETLESGGHNLVQGLENFLRDIKSSGDILNINTTDKSAFKLGQNIAATKGKIIFQNDLMQLICYEPKEKVHKIPIFIIPPCINKYYILDLSSHNSLVSFLVENNFQVFLI<br>SWVNPDTSLSKKGFEDYLKEGILAPFEYVKNLGFAKIDFVGYCMGGMFLAIIIAYFKVKRIDSVHSSTFFTTLLDYTNPGELGIFLNKNTINYIKEEIKLKGYFDGKYLSNSFSLLRANDLIWTFFVNNYLLGKKPMP<br>FDLLYWNADSTNLPAKMYEEYLHNTYCNNLLKESNALEVLGTKIDLGNVDCNSFFLAAKEDHITPWRSIYDGVKLLNGRKIFCLTDSGHVAGVVNHPDNAKYNYRLNYDLSLSSNEWFMQATEYKGSWWNY<br>WIDWLIKNNDTKMLVDSLDYQNLDVIESAPGSYVRR
29: *R. typhi*	257363	PHBC	MYNITHETIINNFKEIANKYQELILHFMQGKGSMIPKSLIDSDKNIIIAYLIIEQFCKNPQKFCQLNIEYIDKLRELTTNSFAKFVGSTSKDIFYTDNRDKRFKDSLWEDNVYFHFVKQYYLLSAAWIKKNIEQYELSH<br>DLKQHLEFTTKHFIDAFSPSNFAFCNPKVLRETLESGGHNLVQGLENFLRDIKSSGDILNINTTDKSAFKLGQNIAATKGKIIFQNDLMQLICYEPKKKVHKIPIFIIPPCINKYYILDLSSHNSLVSFLVENHFQVFLI<br>SWVNPDTSLSEKGFEDYLKEGILAPFEYVKNLGFAKIDFVGYCMGGMFLAIIIAYFKVKSIDSVHSSTFFTTLLDYTNPGELGIFLNENTINYIKEEIKLKGYFDGKYLSNSFSLLRANDLIWTFFVNNYLLGKKPMP<br>FDLLYWNADSTNLPAKMYEEYLHNTYCNNLLKEANALEVLGTKIDLGNIDCNSFFLAAKEDHIAPWRSIYDGVKLLNGDKIFCLTDSGHVAGVVNHPASAKYNYRINYDLSLSSNEWFMQATEYKGSWWNYW<br>IDWLIKNNDTKILVDSLDYQNLDVIEIAPGSYVRR
30: *S. rugosus*	679197	HMPREF9336_0<br>1483	MRLAQEFGNVAARLINTAQNGLEVLRFGGFDIGHYESDHQIVDTKPMAQLRRYFPPGQRDAPRTAPVLLVPPLGVSADVYDISEHGAVTQLREAGLDPWVLDFGSPNRSEREAERSLSDHVLAVVQAIDLIN<br>KHTKQAPHLAGYSQGGMFCYQAAAYRRSEGIASVITFGSPVDVQAGLPLGFGGDTGSDLAEFLADQVLRRLAVPKWMVRTGFQMMDPVKSVRARVDFLRHLHDRELLLPRERQRRFLMSDGWLATSGPA<br>LADFIKAFVVHNRMMTGGLVINGVPVTLAEVTCPVLAFVGSYDAIARPPTVRALPKVASRAAVWEKEIPAGHFGLVVGSLSEREVWPTVAEWIRWQAGERAGRPEDIEKMAVVAKKAAKKPTQASPGLLGLV<br>QQGAGVLVEAGVRAGQDALELGVQVGRTATAFAGEGTRAIPLLIRLGQLRPQTRVSLGLIMAEQARRAPREECFLFADRVHTHEAVGKRVDNVVRGLISVGIRHGDRVGVLMRTRPSALVTIAALNRLGAVA<br>VLIPPWADLRQAVEATGAGAVITDITNLEAARAAGVTVLVLGVAESRDTPDLGPGVVDLEKIDPDKVSLPGWYTPNPGLAKDLAFILVTNSAGHLVATPMTNQRWAVSAFGTATAASLSASDTVYCTSPLHHS<br>AGLVVGWGGAVASGARIALSEYNDVSHNDPERFFEEVHRYGVTVVSYTWTQLRPVLAEMEKRRSAEQRLPIRLFVGSGIPAGQWERVQEQFAPARVVEFFASVQGGAVLANVRGVKPGSKGRPLPGAAR<br>LELGAYDASADQLVLGQRGFVRRAKPGEPGVLIVKPTLEQESFLSRRGIFEQCDEWVITDHIFRRDDDGDFWLLDNRSALIRAQDGPIWSQPILDALDRIPAVDLAVVYRTQTAGQELAVAAVTLRPGARLRAT<br>DLQLGLGPVPASERPHLVRVVPEIPLSPTYRPIGYKLQADGTPRPGRGVWCRDEDGEYAPFTAAKARSLGW
31: *S. maltophilia*	391008	SMAL_2415	MKGPLGFNADDLMQETLAMQRKLMEGLKLLPQVEDVDYGVTAREEVWRDGKVVLYRFVGEQAPTRRTPLLIVYALVNRPYMVDLQADRSLVQKLLALGQDVYVLDWGYPDRSERFQTLEDYLLRYIDGAV<br>DALRARSGGPVDMLGICQGGVFALCYAALRRQKLGKLITMVTPVDFHTADNMLSHWARQVDVDLLVDTLGNIPAELMNASYLMLKPFRLNVQKYVGLLDILDDKAALEDFLRMEKWIFDSPDLAGEAFRDFIK<br>QFYQGNGLMYGTVRIGEEAVDLSKVTLPVLNIYAEQDHLVPPDASRAMRGRLGTEDYTESSFRGGHIGIYVSGRAQREVPATIDGWLKARDA
32: *V. cholera*	243277	VC_A0688	MFQHAFTDYLVQLQQVNQRWWKEVEQSKAAVNSPLNKAMQEVNLEDSLKFFEQAANQPAALLKVQTQWWEQQLQIWQKVVLESKIQSIMEAEKGDKRFSHEAWQQDPFFNFIKQSYLLFSKTYLDTINAIE<br>GLDEKAKERILFFSRQMINALSPSNFIATNPELLRLTLEKNGENLIAGLEQLKEDVASSADILKIRMTNNNAFRLGEDVANTPGEVVFKNEVFELIQYKPLTEQVAVTPLLIVPPFINKYYILDLREKNSMVRWLVE<br>QGHSVFMISWRNPGAAQAQLNFEDYVLEGVVKAVNAIESITGQEQINAAGYCIGGTVLATTIAYYAAKRMKKRIKTASFFTTLLDFSQPGEVGAYINDTIIRAIELQNNAKGYMDGRSLSVTFSLLRENSLYWNY<br>YVDNYLKGQSPVDFDLLYWNSDSTNVAGACHNFLLRELYLENKLVQDKGVKVGGVWIDLDKIKVPSYFISTKEDHIALWQGTYRGALRTGGNKTFVLGESGHIAGIVNHPDKRKYGYWVNDTLDDSAEDWL<br>ETAQHREGSWWVHWNEWLNGFADGSKVEPYPLGNADYPVLYSAPGEYVKQVLPIQEA
33: *V. vulnificus*	216895	VV2_0739	MLQHFFSDYLVKLQETNQQWWQDFEVGKAAVNSPLNKAMQEVNFEDTAKFFEKAANQPQAILQLQTQWWEQQLQIWQNVALSGNTQSIIEADKGDKRFSNEAWQSEAMYSFIKQSYLLFSKTYMDTIDAIE<br>GLDEKTKERISFFSRQAINALSPSNFIATNPELLKLTIEKNGENLLAGMELLREDVESSADILKIRMTNNNAFRIGEDIATTAGDIVFQNDLFELIQYRPLTEQVNATPLLIVPPFINKYYILDLTAKNSMVRWLLEQG<br>HSVFMMSWKNPGKAQANVEFGDYVTEGVVKAVTAIEEITGQEQINAAGYCIGGTVLACTVGYYAAKRMKKRIKSATFFTTLLDFSQPGEVGAYINDTIISAIELQNNAKGYMDGRSLSVTFSLLRENSLYWNYY<br>VDNYLKGSSPVDFDLLYWNSDSTNVAAATHNFMLRELYLNNKLVQDKGVKIGGVWIDLDKIRIPSYFISTKEDHIALWQGTYRGALNMGGNKTFVLGESGHIAGIVNPPAKNKYGFWVNDNLDESADEWLSN<br>AQHKEGSWWTHWDQWLTQFNPAEKVLPYRQGSEANPVIDIAPGQYVKQVLPITE

Supplementary Table 5. Percent Identity Matrix from multiple sequence alignment of 33 known and putative PHA synthases from bacterial human pathogens.

	1: *A. baumannii* (HMPREF0010_00690)	2: *A. calcoaceticus* (BDGL_001038)	3: *A. radioresistens* (HMPREF0018_01802)	4: *B. anthracis* (PHAC)	5: *B. pertussis* (PHBC)	6: *B. cenocepacia* (PHBC)	7: *B. mallei* (PHBC)	8: *B. mallei* (PHAC)	9: *B. multivorans* (BMUL_1483)	10: *B. multivorans* (BMULJ_01759)	11: *B. pseudomallei* (PHBC)	12: *B. pseudomallei* (BPSS1954)	13: *L. pneumophila* (lpg0599)	14: *L. pneumophila* (lpg1058)	15: *L. pneumophila* (lpg1097)	16: *L. pneumophila* (lpg2260)	17: *L. borgpetersenii* (LBL_2592)	18: *L. interrogans* (LA_2034)	19: *M. abscessus* (MAB_2348)	20: *M. avium* (MAP1389)	21: *M. kansasii* (MKANA1_010100013815)	22: *M. leprae* (ML1346)	23: *M. tuberculosis* (MT1723)	24: *N. farcinica* (NFA_45720)	25: *P. aeruginosa* (PhaC1)	26: *P. aeruginosa* (PhaC2)	27: *R. equi* (REQ_24810)	28: *R. prowazekii* (E.RP820)	29: *R. typhi* (PHBC)	30: *S. rugosus* (HMPREF9336_01483)	31: *S. maltophilia* (SMAL_2415)	32: *V. cholera* (VC_A0688)	
1: *A. baumannii* (HMPREF0010_00690)	100.00	94.18	60.00	29.23	21.30	20.69	21.84	19.23	20.98	20.98	22.06	19.23	18.71	20.06	19.29	21.28	18.43	18.82	21.56	18.67	20.23	18.75	18.07	21.02	19.29	18.93	20.62	18.58	18.88	20.38	20.87	18.71	16.37
2: *A. calcoaceticus* (BDGL_001038)	94.18	100.00	60.56	27.69	22.19	21.26	22.41	19.82	21.55	21.55	22.65	19.82	18.71	20.65	19.88	21.57	18.09	17.42	21.56	18.99	20.53	19.06	18.38	21.97	19.29	19.53	20.62	19.47	20.06	21.00	20.56	18.13	16.08
3: *A. radioresistens* (HMPREF0018_01802)	60.00	60.56	100.00	27.30	21.05	18.98	20.68	18.77	19.55	19.55	20.93	18.77	18.79	21.35	19.71	21.90	17.45	16.84	21.30	20.00	20.00	20.99	18.77	23.49	19.65	19.01	20.91	17.78	18.37	21.05	20.19	16.14	15.56
4: *B. anthracis* (PHAC)	29.23	27.69	27.30	100.00	24.78	25.36	25.36	23.68	25.65	25.65	25.37	23.68	23.70	24.56	24.12	24.57	19.93	20.21	21.81	20.45	22.97	21.81	20.19	23.53	17.89	19.30	20.43	24.13	25.00	22.50	31.92	25.72	23.70
5: *B. pertussis* (PHBC)	21.30	22.19	21.05	24.78	100.00	56.55	57.54	35.71	57.30	57.30	57.79	35.90	41.24	32.64	31.62	29.41	16.76	16.72	17.86	17.15	35.89	18.48	18.16	19.20	37.38	38.46	17.65	35.42	35.80	19.06	23.67	38.58	37.08
6: *B. cenocepacia* (PHBC)	20.69	21.26	18.98	25.36	56.55	100.00	83.23	33.63	93.40	93.40	83.78	33.63	38.47	32.76	30.37	28.28	20.22	21.37	17.55	17.87	34.13	17.89	17.59	16.67	36.92	36.55	14.90	35.90	36.08	16.84	24.12	38.96	39.48
7: *B. mallei* (PHBC)	21.84	22.41	20.68	25.36	57.54	83.23	100.00	34.55	84.35	84.35	99.00	34.55	38.93	32.47	30.28	28.09	18.89	19.43	18.04	18.73	35.05	18.64	18.32	17.88	38.10	36.80	14.86	34.97	35.66	16.84	23.24	39.76	38.89
8: *B. mallei* (PHAC)	19.23	19.82	18.77	23.68	35.71	33.63	34.55	100.00	34.33	34.33	34.74	99.83	37.48	49.57	48.19	50.09	20.06	20.90	19.51	21.11	30.92	21.18	20.05	19.81	29.93	32.06	19.26	31.83	32.19	19.95	21.36	33.75	33.57
9: *B. multivorans* (BMUL_1483)	20.98	21.55	19.55	25.65	57.30	93.40	84.35	34.33	100.00	100.00	85.14	34.33	39.51	33.28	30.72	28.79	19.11	20.23	18.09	18.73	34.68	18.68	18.37	16.97	37.48	36.73	15.66	36.08	36.43	17.38	23.82	39.83	39.83
10: *B. multivorans* (BMULJ_01759)	20.98	21.55	19.55	25.65	57.30	93.40	84.35	34.33	100.00	100.00	85.14	34.33	39.51	33.28	30.72	28.79	19.11	20.23	18.09	18.73	34.68	18.68	18.37	16.97	37.48	36.73	15.66	36.08	36.43	17.38	23.82	39.83	39.83
11: *B. pseudomallei* (PHBC)	22.06	22.65	20.93	25.37	57.79	83.78	99.00	34.74	85.14	85.14	100.00	34.74	39.68	32.46	30.42	28.95	18.29	19.83	17.60	18.73	35.01	18.47	18.42	17.99	38.79	37.48	14.81	35.71	36.43	16.94	23.35	40.60	39.89
12: *B. pseudomallei* (BPSS1954)	19.23	19.82	18.77	23.68	35.90	33.63	34.55	99.83	34.33	34.33	34.74	100.00	37.48	49.57	48.19	50.09	20.06	20.90	19.51	21.11	30.92	21.18	20.05	19.81	29.93	32.06	19.26	31.83	32.19	19.95	21.36	33.75	33.57
13: *L. pneumophila* (lpg0599)	18.71	18.71	18.79	23.70	41.24	38.47	38.93	37.48	39.51	39.51	39.68	37.48	100.00	35.00	34.65	34.33	19.77	20.00	15.95	15.59	30.95	15.78	16.27	17.59	32.65	33.77	14.62	39.93	40.45	16.62	19.76	40.00	39.83
14: *L. pneumophila* (lpg1058)	20.06	20.65	21.35	24.56	32.64	32.76	32.47	49.57	33.28	33.28	32.46	49.57	35.00	100.00	83.36	63.59	17.51	17.43	17.98	18.05	30.15	17.79	15.86	18.52	29.13	29.07	17.68	31.16	32.07	15.11	20.77	32.50	30.70
15: *L. pneumophila* (lpg1097)	19.29	19.88	19.71	24.12	31.62	30.37	30.28	48.19	30.72	30.72	30.42	48.19	34.65	83.36	100.00	63.18	17.61	17.43	16.39	16.86	28.65	15.95	15.09	16.98	29.18	29.18	16.49	30.60	31.15	14.33	20.00	31.77	31.23
16: *L. pneumophila* (lpg2260)	21.28	21.57	21.90	24.57	29.41	28.28	28.09	50.09	28.79	28.79	28.95	50.09	34.33	63.59	63.18	100.00	16.71	17.43	18.31	17.16	29.07	17.84	16.71	18.52	27.44	28.36	16.88	30.65	31.36	15.15	20.94	31.73	30.84
17: *L. borgpetersenii* (LBL_2592)	18.43	18.09	17.45	19.93	16.76	20.22	18.89	20.06	19.11	19.11	18.29	20.06	19.77	17.51	17.61	16.71	100.00	88.13	17.43	19.72	16.24	19.02	18.95	16.38	19.25	15.47	16.19	19.36	20.23	19.80	15.46	16.81	15.67
18: *L. interrogans* (LA_2034)	18.82	17.42	16.84	20.21	16.72	21.37	19.43	20.90	20.23	20.23	19.83	20.90	20.00	17.43	17.43	17.43	88.13	100.00	19.93	20.76	16.18	19.54	19.47	15.38	20.35	16.52	16.34	18.99	19.58	21.67	14.29	18.37	17.49
19: *M. abscessus* (MAB_2348)	21.56	21.56	21.30	21.81	17.86	17.55	18.04	19.51	18.09	18.09	17.60	19.51	15.95	17.98	16.39	18.31	17.43	19.93	100.00	66.14	17.34	63.36	64.98	22.70	15.19	19.28	57.68	15.26	16.35	50.92	24.29	17.16	17.43
20: *M. avium* (MAP1389)	18.67	18.99	20.00	20.45	17.15	17.87	18.73	21.11	18.73	18.73	18.73	21.11	15.59	18.05	16.86	17.16	19.72	20.76	66.14	100.00	17.30	82.11	84.02	23.64	17.11	17.06	58.53	16.86	17.75	51.31	25.33	16.28	15.99
21: *M. kansasii* (MKANA1_010100013815)	20.23	20.53	20.00	22.97	35.89	34.13	35.05	30.92	34.68	34.68	35.01	30.92	30.95	30.15	28.65	29.07	16.24	16.18	17.34	17.30	100.00	17.69	17.11	18.27	40.75	39.43	15.97	29.54	29.36	16.94	23.01	33.70	33.52
22: *M. leprae* (ML1346)	18.75	19.06	20.99	21.81	18.48	17.89	18.64	21.18	18.68	18.68	18.47	21.18	15.78	17.79	15.95	17.84	19.02	19.54	63.36	82.11	17.69	100.00	85.67	24.86	16.94	18.80	55.77	15.90	16.71	51.28	25.87	18.04	17.24
23: *M. tuberculosis* (MT1723)	18.07	18.38	18.77	20.19	18.16	17.59	18.32	20.05	18.37	18.37	18.42	20.05	16.27	15.86	15.09	16.71	18.95	19.47	64.98	84.02	17.11	85.67	100.00	22.25	17.17	18.48	57.79	15.86	16.67	51.73	24.84	17.72	17.46
24: *N. farcinica* (NFA_45720)	21.02	21.97	23.49	23.53	19.20	16.67	17.88	19.81	16.97	16.97	17.99	19.81	17.59	18.52	16.98	18.52	16.38	15.38	22.70	23.64	18.27	24.86	22.25	100.00	21.25	19.31	24.29	14.91	14.29	22.99	22.88	16.26	15.95
25: *P. aeruginosa* (PhaC1)	19.29	19.29	19.65	17.89	37.38	36.92	38.10	29.93	37.48	37.48	38.79	29.93	32.65	29.13	29.18	27.44	19.25	20.35	15.19	17.11	40.75	16.94	17.17	21.25	100.00	58.78	15.24	32.16	32.16	16.39	19.64	33.64	33.83
26: *P. aeruginosa* (PhaC2)	18.93	19.53	19.01	19.30	38.46	36.55	36.80	32.06	36.73	36.73	37.48	32.06	33.77	29.07	29.18	28.36	15.47	16.52	19.28	17.06	39.43	18.80	18.48	19.31	58.78	100.00	16.53	34.20	33.09	19.67	21.66	33.77	34.14
27: *R. equi* (REQ_24810)	20.62	20.62	20.91	20.43	17.65	14.90	14.86	19.26	15.66	15.66	14.81	19.26	14.62	17.68	16.49	16.88	16.19	16.34	57.68	58.53	15.97	55.77	57.79	24.29	15.24	16.53	100.00	15.76	15.50	50.87	25.16	15.90	15.13
28: *R. prowazekii* (E.RP820)	18.58	19.47	17.78	24.13	35.42	35.90	34.97	31.83	36.08	36.08	35.71	31.83	39.93	31.16	30.60	30.65	19.36	18.99	15.26	16.86	29.54	15.90	15.86	14.91	32.16	34.20	15.76	100.00	95.72	15.93	18.99	37.80	37.80
29: *R. typhi* (PHBC)	18.88	20.06	18.37	25.00	35.80	36.08	35.66	32.19	36.43	36.43	36.43	32.19	40.45	32.07	31.15	31.36	20.23	19.58	16.35	17.75	29.36	16.71	16.67	14.29	32.16	33.09	15.50	95.72	100.00	16.48	18.99	37.80	38.33
30: *S. rugosus* (HMPREF9336_01483)	20.38	21.00	21.05	22.50	19.06	16.84	16.84	19.95	17.38	17.38	16.94	19.95	16.62	15.11	14.33	15.15	19.80	21.67	50.92	51.31	16.94	51.28	51.73	22.99	16.39	19.67	50.87	15.93	16.48	100.00	26.90	17.30	17.57
31: *S. maltophilia* (SMAL_2415)	20.87	20.56	20.19	31.92	23.67	24.12	23.24	21.36	23.82	23.82	23.35	21.36	19.76	20.77	20.00	20.94	15.46	14.29	24.29	25.33	23.01	25.87	24.84	22.88	19.64	21.66	25.16	18.99	18.99	26.90	100.00	22.87	22.29
32: *V. cholera* (VC_A0688)	18.71	18.13	16.14	25.72	38.58	38.96	39.76	33.75	39.83	39.83	40.60	33.75	40.00	32.50	31.77	31.73	16.81	18.37	17.16	16.28	33.70	18.04	17.72	16.26	33.64	33.77	15.90	37.80	37.80	17.30	22.87	100.00	79.15
33: *V. vulnificus* (VV2_0739)	16.37	16.08	15.56	23.70	37.08	39.48	38.89	33.57	39.83	39.83	39.89	33.57	39.83	30.70	31.23	30.84	15.67	17.49	17.43	15.99	33.52	17.24	17.46	15.95	33.83	34.14	15.13	37.80	38.33	17.57	22.29	79.15	100.00

Supplementary Table 6. Strains, plasmids and oligonucleotides used in this study

**Strain or plasmid or oligo**	**Description**	**Reference**
E. coli		
XL1-Blue	*recA1 endA1 gyrA96 thi-1 hsdR17 supE44 relA1 lac* [F' *proAB lacI*^{q} *lacZ* Δ*M15* Tn*10* (Tet^r)]	Stratagene
ClearColi BL21(DE3)	F– *ompT hsdSB (rB- mB-) gal dcm lon* λ(DE3 [*lacI lac*UV5-T7 gene 1 *ind1 sam7 nin5*]) *msbA148* Δ*gut*QΔ*kds*D Δ*lpxL*Δ*lpxM*Δ*pagP*Δ*lpxP*Δ*eptA*	Lucigen
P. aeruginosa		
P. aeruginosa PAO1	Prototroph, non-mucoid	ATCC 15692
PAO1 Δ*phaC1ZC2*	PHA-negative mutant of PAO1 lacking functional *phaC1*$_{Pa}$ and *phaZ*	[1]
PAO1 Δ*phaC1ZC2* Δ*alg8*	PAO1 Δ*phaC1ZC2* derivative with markerless, isogenic *alg8* deletion, double mutant	This study
PAO1 Δ*phaC1ZC2* Δ*alg8* Δ*pelF*	PAO1 Δ*phaC1ZC2* Δ*alg8* derivative with markerless, isogenic *pelF* deletion, triple mutant	This study
**Plasmids**		
pEX100T::Δ*pelF* ΩGm	Amp^r Cb^r Gm^r, vector pEX100T with SmaI-inserted *pelF* deletion construct	[2]
pEX100T::Δ*alg8* ΩGm	Amp^r Cb^r Gm^r; vector pEX100T with SmaI-inserted *alg8* deletion construct	[3]
pFLP2	Amp^r Cb^r; broad-host-range vector encoding Flp recombinase	[4]
pGEM-T easy	Cloning vector, f1 origin, Amp^r	Promega
pUC57	Cloning vector, ColE1 origin, Amp^r	Fermentas
pUC57_Ag	pUC57 derivative containing *E. coli* codon optimized OprI/F-AlgE fusion antigen fragment (OprI-OprF(x3)-L6-L5) flanked by NdeI/BamHI sites	This study
pET16b	Amp^r, T7 promoter, His$_{10}$-tag	Novagen
pET16b-HisAg	pET16b derivative containing His$_{10}$-tagged *E. coli* codon optimized OprI/F-AlgE fusion antigen fragment (OprI-OprF(x3)-L6-L5) in NdeI/BamHI sites of pET16b	This study
pBHR71	pBluescript SK- derivative; Amp^r; P$_{lac}$; containing *phaC1* gene coding for PHA$_{MCL}$ synthase of *P. aeruginosa* PAO1	[5]
pBBR1MCS-5	Gm^r; broad-host-range vector; P$_{lac}$	[6]
pBBR1JO-5	Gm^r, P$_{lac}$, pBBR1MCS-5 with MCS from pBluescript SK□-	[7]
pBBR1JO-5_C1	pBBR1JO-5 derivative containing Shine-Dalgarno-*phaC1* fragment from pBHR71 in XbaI/BamHI sites of pBBR1JO-5	This study
pGEM-T_C1(-)	PHA$_{MCL}$ synthase gene fragment amplified from pBHR71 with stop codon removed flanked by *XbaI/Bam*HI	This study
pBBR1JO-5_C1(-)	pBBR1JO-5 derivative containing XbaI/BamHI inserted PHA$_{MCL}$ synthase gene fragment from pGEM-T_C1(-)	This study
pET-14b PhaC-linker-SG- linker-GFP	pET-14b PhaC-linker-GFP derivative containing the SG linker sequence upstream of *gfp*	[8]
pGEM-T_LSGL*gfp*	LSGL*gfp* fragment with flanking BglII/BamHI sites amplified from pET-14b PhaC-linker-SG- linker-GFP	This study
pBBR1JO-5_C1*gfp*	BglII/BamHI LSGL*gfp* fragment from pGEM-T_LSGL*gfp* inserted into BamHI site of pBBR1JO-5_C1(-) downstream of 3' end of *phaC1*$_{Pa}$	This study

pHERD20T	Amp^r Cb^r, pUCP20T P_{lac} replaced with fragment of *araC*-P_{BAD} cassette	[9]
pHERD20T-2	pHERD20T derivative were a 13 bp fragment of 5' end of LacZα is removed	This study
pHERD20T-2_C1	Shine-Dalgarno-*phaC1*$_{Pa}$ fragment from pBBR1JO-5_C1 inserted in to XbaI/HindIII site of pHERD20T-2	This study
pUC57_Ag(N)	pUC57 derivative containing *P. aeruginosa* codon optimized OprI/F-AlgE fusion antigen fragment (L5-L6-OprF(x3)-OprI-Linker (GSGGG) flanked by XbaI/NdeI sites	This study
pHERD20T-2_AgC1	Codon optimized Ag fragment from pUC57_Ag(N) inserted in to XbaI/NdeI sites of pHERD20T-2_C1 upstream of *phaC1*$_{Pa}$	This study
pUC57_Ag(C)	pUC57 derivative containing *P. aeruginosa* codon optimized OprI/F-AlgE fusion antigen fragment (OprI-OprF(x3)-L6-L5) flanked by SmaI/EcoRI sites	This study
pBBR1JO-5_C1Ag	Condon optimized OprI/F-AlgE fusion antigen fragment from pUC57_Ag(C) inserted in to SmaI/EcoRI site of pBBR1JO-5_C1gfp replacing GFP	This study
pHERD20T-2_C1Ag	*phaC1*$_{Pa}$-LSGL-Ag fragment from pBBR1JO-5_C1Ag inserted in to XbaI/HindIII sites of pHERD20T-2	This study
**Oligonucleotides**		
Alg8_XUP	5' GCGTCGAGGCCAAGGTCCC	[2]
Alg8_XDN	5' CCTGGCGTTGTCCGTAGTCG	[2]
PelF_XUP	5' ACATGCTGCAACGGCCGCCCT	[2]
PelF_XDN	5' TAGGCGCGCAGGGTCGCCGTA	[2]
F_phaC1	5' AAATCTAGAAATAAGGAGATATACATATGAGTCAGAAGAACAATAACGAG	This study
R_phaC1_(-)stop_BamHI	5' ATTGGATCCTCGTTCATGCACGTAGGTTCCG	This study
F_BglII_LSGLgfp	5' AATAGATCTGTGCTGGCGGTGGCGATTGATAAACGCGG	This study
R_LSGLgfp_BamHI	5' GCCGGATCCTCATTTGTATAGTTCATCCATGCCATGTG	This study

1. Pham TH, Webb JS, Rehm BHA. The role of polyhydroxyalkanoate biosynthesis by *Pseudomonas aeruginosa* in rhamnolipid and alginate production as well as stress tolerance and biofilm formation. *Microbiology* **150**, 3405-3413 (2004).

2. Ghafoor A, Hay ID, Rehm BHA. Role of exopolysaccharides in *Pseudomonas aeruginosa* biofilm formation and architecture. *Applied and environmental microbiology* **77**, 5238-5246 (2011).

3. Remminghorst U, Rehm BHA. In vitro alginate polymerization and the functional role of Alg8 in alginate production by *Pseudomonas aeruginosa*. *Applied and environmental microbiology* **72**, 298-305 (2006).

4. Hoang TT, Karkhoff-Schweizer RR, Kutchma AJ, Schweizer HP. A broad-host-range Flp-FRT recombination system for site-specific excision of chromosomally-located DNA sequences: application for isolation of unmarked Pseudomonas aeruginosa mutants. *Gene* **212**, 77-86 (1998).

5. Langenbach S, Rehm BHA, Steinbuchel A. Functional expression of the PHA synthase gene PhaC1 from *Pseudomonas aeruginosa* in *Escherichia coli* results in poly(3-hydroxyalkanoate) synthesis. *Fems Microbiology Letters* **150**, 303-309 (1997).

6. Kovach ME, *et al.* Four new derivatives of the broad-host-range cloning vector pBBR1MCS, carrying different antibiotic-resistance cassettes. *Gene* **166**, 175-176 (1995).

7. Peters V, Rehm BHA. In vivo monitoring of PHA granule formation using GFP-labeled PHA synthases. *FEMS microbiology letters* **248**, 93-100 (2005).

8. Jahns AC, Rehm BHA. Tolerance of the *Ralstonia eutropha* class I polyhydroxyalkanoate synthase for translational fusions to its C terminus reveals a new mode of functional display. *Applied and environmental microbiology* **75**, 5461-5466 (2009).

9. Qiu D, Damron FH, Mima T, Schweizer HP, Hongwei DY. PBAD-based shuttle vectors for functional analysis of toxic and highly regulated genes in *Pseudomonas* and *Burkholderia* spp. and other bacteria. *Applied and environmental microbiology* **74**, 7422-7426 (2008).
